# Protective effects of dietary nutrients on hearing loss: a systematic review and meta-analysis

**DOI:** 10.3389/fnut.2025.1528771

**Published:** 2025-05-09

**Authors:** Wang Lu, Rui Tang, Xiong Jiahui, Zhang Shipeng, Guo Tao, Wang Hanyua, Xiong Feng, Xie Hui

**Affiliations:** ^1^Clinical Medical College, Chengdu University of Traditional Chinese Medicine, Chengdu, China; ^2^Chengdu Institute of Biology, Chinese Academy of Sciences, Chengdu, China; ^3^Department of Psychiatry and Psychotherapy, TUM School of Medicine and Health, Klinikum Rechts der Isar, Technical University of Munich, Munich, Germany; ^4^Department of Orthopedics, Jiang'an County Traditional Chinese Medicine Hospital, Yibin, Sichuan, China; ^5^Otolaryngology, Hospital of Chengdu University of Traditional Chinese Medicine, Chengdu, China

**Keywords:** hearing loss, dietary nutrients, systematic review, meta-analysis, antioxidants

## Abstract

**Background:**

Hearing loss ranks as the third most prevalent disability globally, significantly impacting individuals and society, and imposing a substantial healthcare burden. The World Health Organization reports that over 1.5 billion people worldwide experience hearing loss, with one-third of these cases attributed to preventable factors. Recently, the influence of diet and nutrition on auditory health has garnered increasing attention.

**Objective:**

This study systematically reviews and meta-analyzes the protective effects of dietary nutrients on hearing, examining specific nutrients' impact on hearing loss and their potential biological mechanisms.

**Methods:**

A comprehensive search of PubMed, Embase, Web of Science, and the Cochrane Library was conducted for relevant studies up to August 2024. Following PRISMA guidelines, the systematic review was registered in PROSPERO. Included were observational studies assessing the relationship between dietary intake and hearing loss.

**Results:**

Thirty-three studies met inclusion criteria: 21 cross-sectional, 10 cohort, and 2 case-control studies. Meta-analysis revealed significant inverse associations between the intake of vitamin B2, β-carotene, carotenoids, β-cryptoxanthin, fat, protein, fiber, and fish, and the risk of hearing loss.

**Conclusion:**

Certain dietary nutrients may protect hearing health. Increasing intake of antioxidants, fiber, protein, and fish rich in unsaturated fatty acids may help preserve auditory function.Keywords: Hearing loss, dietary nutrients, systematic review, meta-analysis, antioxidants.

**Systematic review registration:**

https://www.crd.york.ac.uk/PROSPERO/view/CRD42024572118, identifier: CRD42024572118.

## Graphical Abstract

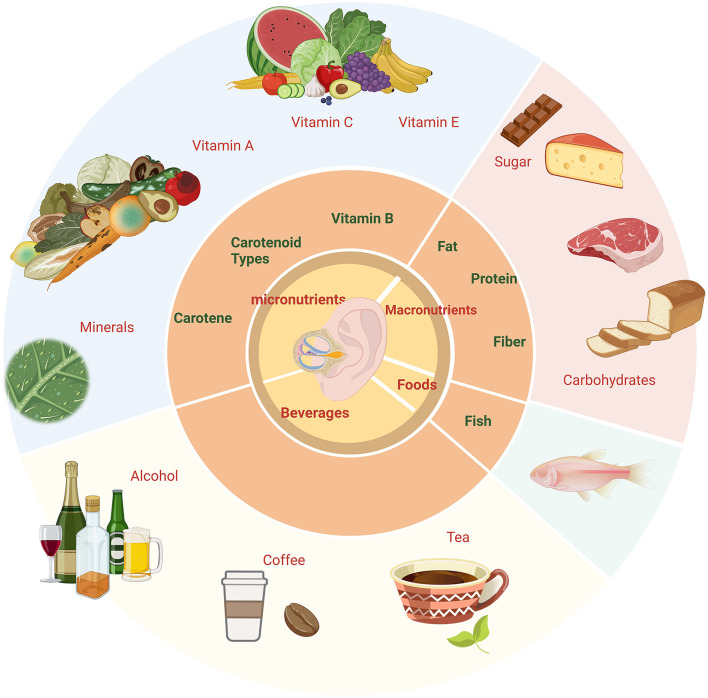

## 1 Introduction

Hearing loss has become one of the major global health challenges (1), not only impacting an individual's communication abilities and quality of life, but also being strongly associated with cognitive dysfunction (2, 3), depression, and social isolation (2). It profoundly affects education and employment as well. As a condition that affects individuals across their lifespans, hearing loss is characterized by high prevalence rates, a continuous upward trend, and a global reach. According to the World Health Organization (WHO), over 1.5 billion people worldwide currently experience some degree of hearing loss, a number that could rise to 2.5 billion by 2050, with over 700 million people (or 1 in 10) projected to suffer from disabling hearing loss (4). In the coming decades, the number of people with hearing loss is expected to increase significantly, presenting a more urgent challenge than ever before. WHO data indicates that the global annual cost of unaddressed hearing loss exceeds $980 billion (4). As the third leading cause of disability worldwide, hearing loss imposes severe consequences on both individuals and society, creating an substantial burden on healthcare systems. It is therefore essential to acknowledge that nearly one-third of hearing loss cases stem from preventable factors (5, 6), making preventive health measures significantly more meaningful and cost-effective than treatment. In recent years, the potential impact of diet and nutrition on hearing health has gained increasing attention. Growing evidence suggests that dietary habits and nutritional intake not only affect an individual's general health, but may also influence hearing function through complex biological mechanisms (7).

The auditory system, particularly inner ear structures, depends on general health and good metabolic status. Studies have shown that healthy diets can help delay or prevent age-related hearing deterioration (8). The relationship between diet, beverage and hearing health involves multiple biological mechanisms, including antioxidant defense, inflammatory regulation and metabolic support. Dietary antioxidants, vitamins and minerals may protect the auditory system by reducing free radical production and maintaining cochlear health. Specific dietary factors and nutrients, such as antioxidants, anti-inflammatory components, vitamins and minerals, may play key roles in protecting the inner ear and maintaining auditory function. For example, antioxidant nutrients (such as vitamins A, C, and E) are believed to protect inner ear cells by neutralizing free radicals and reducing oxidative stress, thereby slowing the progression of age-related hearing loss. In contrast, diets high in sugar and fat may increase the risk of hearing loss by promoting inflammatory responses and oxidative stress (6). Although these underlying mechanisms have been initially confirmed through basic experimental studies (7), research findings at the population level are still inconclusive, highlighting the need for more systematic and comprehensive evidence to establish a clear link between dietary and beverage intaking and hearing health.

How dietary habits affect hearing health is a complex and multilayered issue. Although some studies have explored the relationship between certain nutrients (such as vitamins A, C, and E, zinc, and magnesium) and hearing loss (9), there is still a lack of systematic reviews and Meta-analyses of the effects of dietary factors and nutritional types on hearing. Some cross-sectional studies and longitudinal cohort studies have provided preliminary evidence suggesting that healthy dietary patterns may contribute to hearing preservation, while others have failed to find significant associations. To address these issues, our study conducts a systematic review and meta-analysis to synthesize and evaluate existing research on the relationship between dietary and nutritional factors and hearing loss. The dietary nutrition types of the review are shown in Graphical Abstract as follows. The objectives are to identify and validate specific dietary patterns and nutrients that are significantly associated with hearing health, thereby providing data support. Furthermore, the study investigates the potential protective effects of certain dietary components or nutrients on hearing, exploring the underlying mechanisms involved in inner ear protection and auditory nerve function maintenance.

## 2 Methods

This meta-analysis strictly adheres to the Preferred Reporting Items for Systematic Reviews and Meta-Analyses (PRISMA) guidelines (10) and has been registered in the PROSPERO database (Registration number: CRD42024572118).

### 2.1 Search strategy

We performed a comprehensive literature search in PubMed, Embase, Web of Science, and Cochrane Library (detailed in Supplementary material 1) up to August 2024, including only English-language studies on human subjects. Additionally, the reference lists of included studies were cross-checked to ensure maximum coverage of relevant literature.

### 2.2 Inclusion and exclusion criteria (based on the PICO framework)

#### 2.2.1 Inclusion criteria

Following recommendations (11), we used the PICO(S) framework (12) to define the review question:

P—Population: Studies that included the general population and individuals diagnosed with various types of hearing loss were considered. The studies needed to clearly report demographic characteristics such as age, gender, ethnicity, and hearing status of the participants.

I—Intervention: Included studies had to examine any form of dietary or nutritional intake and provide detailed descriptions of dietary patterns, specific food types, or nutrient intake.

C—Comparison: Control groups could consist of individuals not exposed to specific diets or nutrients or those following different dietary patterns.

O—Outcomes: Hearing loss had to be the primary outcome, including sensorineural, conductive, and mixed hearing loss. The diagnosis of hearing loss must be based on standardized diagnostic tools, such as pure-tone audiometry.

S—Study Design: Only observational studies were included, comprising prospective studies, cross-sectional studies, and case-control studies. Articles had to provide odds ratios (OR), relative risks (RR), or prevalence ratios with 95% confidence intervals (CI), or data sufficient to calculate these measures (Table 1).

**Table 1:** Use of the PICOTS format, as applied to this study.

**PICOTS format**	**Description**
Population	General population and individuals with hearing loss.
Intervention	Studies on dietary or nutritional intake, detailing patterns, food types, or nutrients.
Comparison	Control groups without specific diets or with different dietary patterns.
Outcomes	Primary outcome: hearing loss (sensorineural, conductive, mixed), assessed by standard tools like pure-tone audiometry.
Time	Not specified.
Setting	Not specified.
Study d esign	Observational studies (prospective, cross-sectional, case-control) with odds ratios, relative risks, or prevalence ratios, or data for calculation.

#### 2.2.2 Exclusion criteria

(1) Studies that did not include participants with undiagnosed hearing loss, or studies that failed to clearly report demographic characteristics (such as age, gender, ethnicity and hearing status). (2) Studies that did not involve any form of dietary or nutritional intake, or those that did not clearly describe or specify the dietary patterns, food types, or nutrient intake, or where the exposure variables were unrelated to diet and nutrition (such as lifestyle, environmental factors and medication use). (3) Studies where hearing loss was not an outcome, or where hearing loss assessment did not use standardized diagnostic tools (e.g., lack of audiometric testing or professional questionnaires). (4) Randomized controlled trials, experimental studies, reviews, commentaries, case reports, brief reports, or studies with incomplete data or insufficient detail to conduct a meta-analysis. (5) Studies with serious flaws in the study design or methodology that affect the validity and reliability of the results. (6) ARHL and Non-ARHL studies were coded separately. Mixed-population studies were excluded unless subgroup data were provided.

### 2.3 Study selection and data extraction

The studies were selected by two independent authors (LW and RT). These authors used a pre-specified form to extract data, including the author, publication date, study region, study type, ethnicity, total sample size, sample size of the diseased group, sample size of the control group, gender, age, exposure assessment methods, hearing loss assessment/diagnosis methods, type of hearing loss, and the effect sizes (e.g., OR, RR) with their 95% confidence intervals for all categories of dietary nutrient intake in each study, as well as covariates used for adjustment. Data extraction followed the methods recommended in the Cochrane Handbook for Systematic Reviews of Interventions (13). Definition of hearing loss subtypes, for each study, only the OR comparing the highest intake category with the reference group is extracted. When there is suspicion of population overlap, we adopted the strategy of prioritizing studies with the largest sample size or the longest follow-up time to ensure data independence and avoid duplicate calculations.

When extracting data, it should be done by age group. Age-Related Hearing Loss (ARHL): Defined as bilateral, progressive sensorineural hearing loss in individuals aged ≥60 years, excluding known causes (e.g., noise exposure, ototoxic drugs, genetic mutations, or infections). Diagnosed by pure-tone audiometry with elevated thresholds at high frequencies (≥4 kHz). Non-Age-Related Hearing Loss (Non-ARHL): included studies on hearing loss with identifiable etiologies (e.g., noise-induced, ototoxic, infectious, or congenital).

### 2.4 Dietary exposure assessment and measurement of hearing loss

The diversity in dietary exposure and hearing loss measurement methods across the included studies reflects the varying research designs and objectives. Dietary Exposure Assessment Methods: (1) Multiple 24-h Dietary Records: Several studies employed multiple 24-h dietary records, requiring participants to document all food and beverage intake over multiple days. This method provides detailed short-term dietary intake data (6, 9, 14–24). (2) Food Frequency Questionnaire (FFQ): A portion of the studies used the FFQ to assess participants' food intake frequency over a specified period (typically 1 month or 1 year), making it a common tool for evaluating long-term dietary habits (25–33). (3) Short Food Frequency Questionnaire (SFFQ): in some studies, the SFFQ was used as a simplified tool to quickly assess dietary patterns and nutrient intake (34–36). (4) Questions and Answers: a few studies used a question-and-answer format to assess dietary exposure, relying on standardized questions to gather information about participants' dietary habits and preferences. This method is dependent on self-reported data (37–44). All dietary and nutritional intake data were included in the meta-analysis considering extreme values.

Hearing Loss Measurement Methods: (1) Self-Reported Subjective Hearing Loss: Some studies employed self-reported methods, where participants subjectively assessed and reported their hearing status. This approach is simple and suitable for preliminary screening in large populations but relies on participants' self-perception. (2) Audiometry Examination: Audiometric testing was used in several studies to provide an objective assessment of hearing loss. This method involves various auditory tests to evaluate hearing function and the degree of hearing impairment. (3) Pure-Tone Audiometry: Pure-tone audiometry is one of the most commonly used methods for hearing assessment, measuring hearing thresholds at multiple frequencies (e.g., 0.5, 1, 2, and 4 kHz). It provides precise data for the diagnosis and classification of hearing loss. (4) Digits Triplet Test (DTT): The DTT is used to measure speech hearing, particularly the ability to recognize speech in noise. Although this method is suitable for more detailed hearing loss assessment, it was used less frequently in the included studies.

### 2.5 Literature quality assessment/risk of bias

The risk of bias for the included studies was assessed by two independent authors (RT and JX) using the Newcastle-Ottawa Scale (NOS), a tool widely applied for assessing the risk of bias in observational studies. Initially developed for cohort studies, we used an adapted version of NOS for cross-sectional studies (45). This adapted version has been used by several other studies that have recognized the need to adapt the NOS scale to properly assess the quality of cross-sectional studies. The scale consists of three domains: selection, comparability, and outcome. Each study was independently evaluated by two reviewers, and any disagreements were resolved through consensus or with the help of a third reviewer.

### 2.6 Sensitivity and publication bias analysis

We used Egger's regression and funnel plots to explore publication bias (46). If significant publication bias was present (i.e., *p* ≥ 0.05), the trim-and-fill method was used to assess the impact of potentially missing studies on the combined effect size. We also conducted sensitivity analyses to evaluate the robustness of the results by omitting one study at a time (47). The overall effect size was recalculated after excluding each study, and significant changes in effect size were noted. If no significant change was observed after excluding a study, the results were considered robust (48).

### 2.7 Data analysis

All data were systematically reviewed based on the types of dietary nutrient intake and were meta-analyzed where appropriate. A minimum of three studies was required for quantitative synthesis in the meta-analysis (49). Meta-analytic statistics were performed by combining OR and their 95% CI. The heterogeneity among the included studies was assessed using the I^2^ statistic (50). When low heterogeneity was observed (I^2^ < 50%), a fixed-effect model was used. In cases of high heterogeneity (I^2^ ≥ 50%), a random-effects model was employed. We tested the robustness of the results through sensitivity analysis (reference), and, when necessary, conducted subgroup analyses to explore the sources of heterogeneity (50). The results of the main effect estimates were reported in forest plots. Subtypes were analyzed separately if sufficient data were available. After a comprehensive analysis, pooled odds ratios (ORs) were calculated separately for ARHL and Non-ARHL subgroups. Sensitivity analyses tested robustness after excluding studies with overlapping etiologies. Strive to minimize the confounding effect of age on the results. All statistical analyses were conducted using STATA software (Version 17, StataCorp LP, College Station, Texas, United states).

## 3 Result

**Figure 1:**
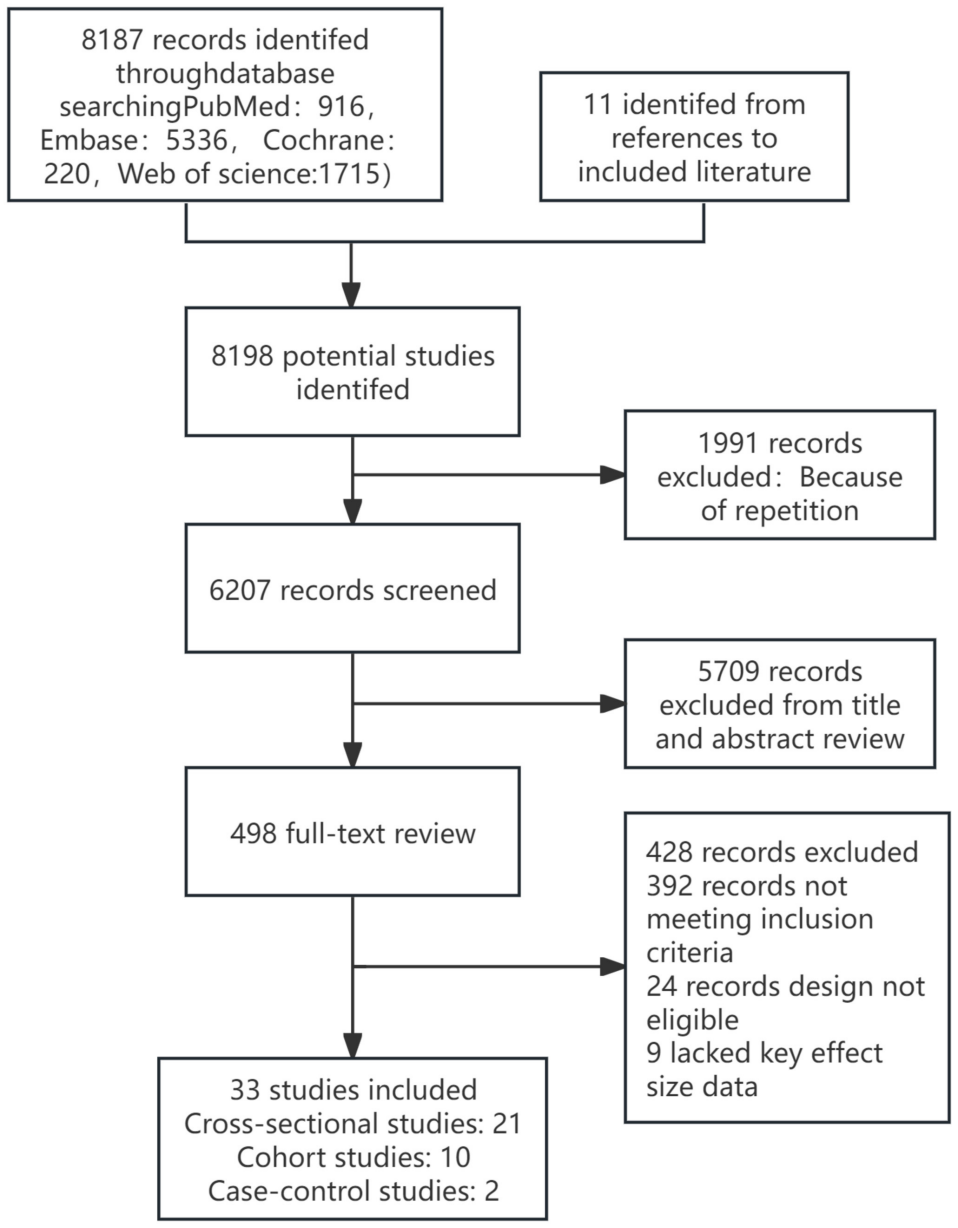
Flow chart.

The electronic database search identified and screened a total of 8,187 abstracts (PubMed: 916, Embase: 5,336, Cochrane: 220, Web of Science: 1,715). After excluding 1,991 duplicate studies, 6,207 studies were excluded based on abstract review due to irrelevance to the topic. A total of 498 articles proceeded to full-text eligibility assessment, where 24 articles were excluded as they were non-observational studies, 9 were excluded for incomplete or unavailable data, and 392 were excluded for not meeting the inclusion criteria. Ultimately, 33 studies met the inclusion criteria, including 11 studies related to hearing loss (unspecified type), 20 studies on age-related hearing loss, 1 study on sudden sensorineural hearing loss, and 1 study on noise-induced hearing loss. Among these, 21 were cross-sectional studies, 10 were cohort studies, and 2 were case-control studies. Specifically, it can be found in Figure 1. The key characteristics and detailed information of all included studies are provided in the Supplementary Figure 2. The overall risk of bias assessment for the observational studies (n = 33) was deemed acceptable, primarily due to bias arising from confounding and exposure classification. The risk of bias assessment for non-randomized studies, conducted using the NOS, ranged from 5 to 9 points, with an average score of 7.9 for cross-sectional studies, 7.1 for cohort studies, and 6.3 for case-control studies. The Newcastle-Ottawa Scale is available in the Supplementary Table 2.

### 3.1 Micronutrients

#### 3.1.1 Vitamin A

In the meta-analysis of 12 studies on vitamin A, the pooled OR was 0.979 (95% CI: 0.902–1.062, p = 0.023, I^2^ = 50.3%) (Table 2). Despite the significant *p*-value, the CI includes 1, making a significant inverse association between vitamin A intake and hearing loss inconclusive. Egger's test did not indicate significant small-study bias (*p* = 0.146), and sensitivity analysis confirmed the robustness of the results (Supplementary material 1).

**Table 2:** Main results of meta-analysis of dietary nutrition and hearing loss.

**Food/ nutrient type**	**Meta-analysis results**	**Subgroup**	**Number of studies**	**Pooled OR**	**95% Confidence interval**	**I^2^(%)**	***p* value**	**Egger's test**	**Sensitivity analyses**
Micronutrients	Vitamin A	Overall	12	0.979	0.902, 1.062	50.3	0.023	0.146	Robust
		Vitamin A	7	1.05	0.980, 1.127	0	0.713		
		Retinol	5	0.839	0.697, 1.008	76.9	0.002		
	Vitamin B	Overall	21	0.99	0.957–1.024	44.8	0.549	0.257	Robust
		Vitamin B1	4	0.915	0.812–1.031	30.1	0.144		Robust
		Vitamin B2	4	0.835	0.731–0.954	21.6	0.008		Robust
		Vitamin B3	6	0.992	0.921–1.069	58.4	0.834		Robust
		Vitamin B6	2	1	0.928–1.078	0	0.993		Robust
		Vitamin B9	3	0.985	0.923–1.052	28.9	0.651		Robust
		Vitamin B12	2	1.087	1.001–1.180	0	0.047		Robust
	Vitamin C	NA	9	0.988	0.896–1.088	58.7	0.804	0.964	Robust
	Vitamin E	NA	5	1.019	0.909–1.142	58.3	0.747	0.529	Robust
	Carotenes	Overall	13	0.968	0.934–1.002	5.6	0.067	0.432	Robust
		Renieratene	6	1	0.941–1.062	0	0.99		Robust
		β-renieratene	6	0.932	0.887–0.980	19.2	0.006		Robust
		α-renieratene	1	1.01	0.929–1.098	NA	0.816		Robust
	Carotenoid types	Overall	7	0.928	0.885–0.972	63.1	0.002	0.7	Robust
		β-cryptoxanthin	2	0.926	0.867–0.990	35.1	0.024		Robust
		Lycopene	2	0.951	0.871–1.039	65	0.267		Robust
		Xanthophyll	2	0.958	0.900–1.019	0	0.174		Robust
		Renieratene	1	0.84	0.787–0.897	NA	0		Robust
	Minerals	Overall	37	0.994	0.957–1.033	15.9	0.769	0.717	Robust
		Magnesium	2	1.044	0.916–1.189	58.3	0.521		Robust
		Calcium	6	1.021	0.950–1.097	0	0.576		Robust
		Iron	6	0.977	0.902–1.059	0	0.572		Robust
		Potassium	7	1.027	0.932–1.131	49.1	0.592		Robust
		Zinc	1	1.16	0.620–2.170	NA	0.642		Robust
		Phosphorus	5	0.953	0.851–1.068	18.3	0.407		Robust
		Ash	5	0.975	0.834–1.140	28.5	0.75		Robust
		Sodium	5	0.899	0.780–1.037	15.5	0.144		Robust
Macronutrients	Fat	Overall	26	0.961	0.908–1.018	56.8	0.174	0.213	Robust
		Non-fatty acids	9	0.92	0.855–0.991	64.9	0.028		Robust
		Saturated fat	5	1.084	0.957–1.229	14.8	0.205		Robust
		Trans fat	3	1.209	0.788–1.855	81.5	0.385		Robust
		Fat (unspecified)	9	0.918	0.850–0.991	0	0.028		Robust
	Protein	NA	9	0.87	0.806–0.939	0	0	0.49	Robust
	Fiber	NA	10	0.923	0.854–0.998	0	0.044	0.479	Robust
	Carbohydrates	NA	10	0.901	0.781–1.040	57.2	0.153	0.632	Robust
	Carbohydrates (sugar)	NA	9	1.087	0.996–1.187	55.9	0.02	0.015	Robust
	Sugar (trim and fill)^*^	NA	11	1.065	0.974–1.164	45.3	0.168	NA	
Beverages	Alcohol	NA	9	0.938	0.762–1.155	93.7	0.549	0.685	Robust
	Coffee	NA	10	0.886	0.764–1.026	53	0.024	0.791	Robust
	Tea	NA	6	1.065	0.895–1.268	49.1	0.08	0.756	Robust
Foods	Fish	NA	4	0.868	0.759–0.994	0	0.47	0.088	Robust

^*^The result after trim and fill.

**Figure 2:**
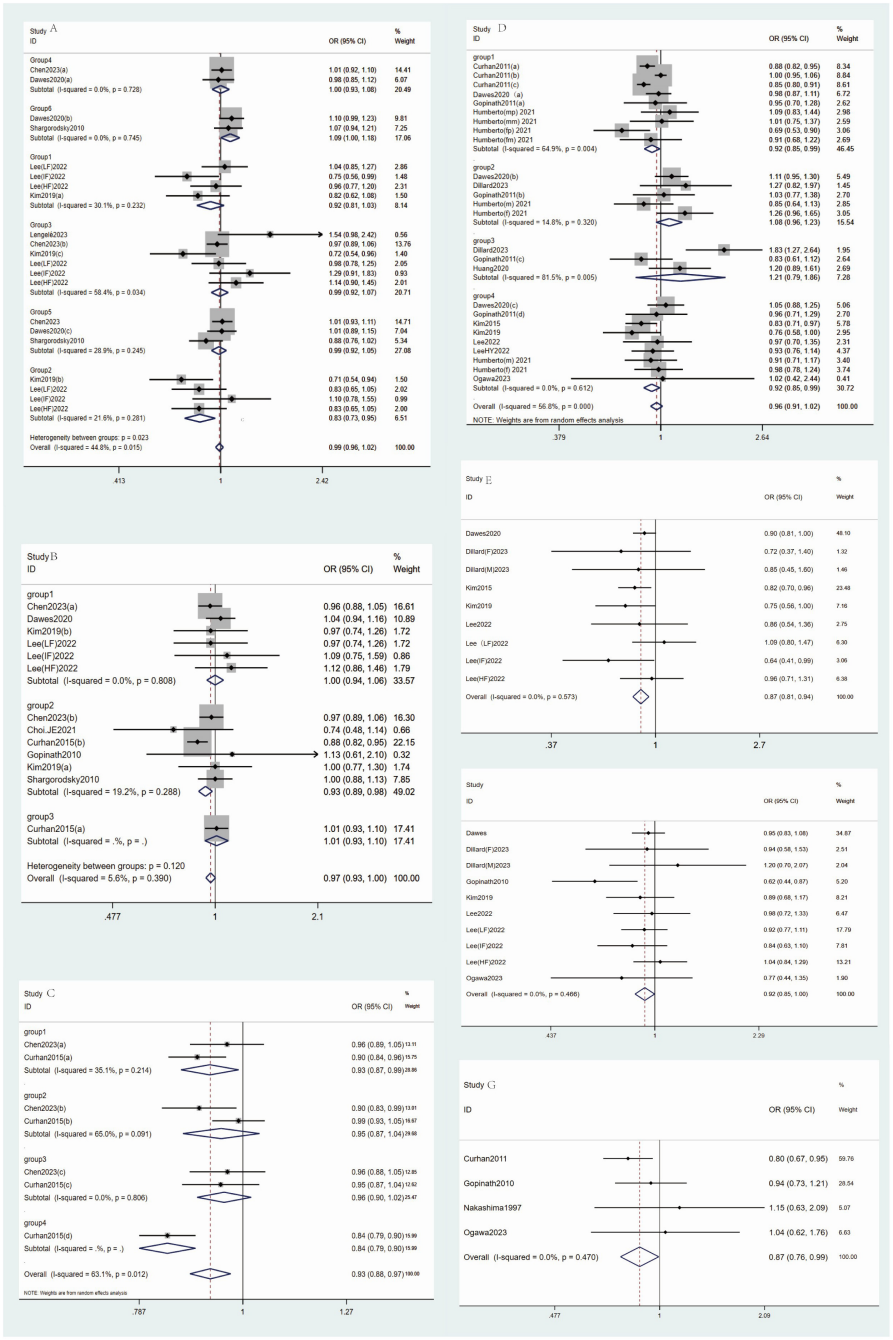
The main forest plot of the meta-analysis. **(A)** Forest maps for Vitamin B intake and incidence of hearing loss (group1: vitamin B1, group2: vitamin B2, group3: vitamin B3, group 4: vitamin B6, group5: vitamin B9, group6: vitamin B12). **(B)** Forest maps for Carotene intake and incidence of hearing loss. **(C)** Forest maps for Carotenoid Types intake and incidence of hearing loss (group1: β-cryptoxanthin; group2: lycopene; group3: xanthophyll; group4: renieratene); **(D)** Forest maps for Fat intake and incidence of hearing loss [group1: Non-fatty acids; group2: Saturated fat; group3: Trans fat; group4: Fat (unspecified)]; **(E)** Forest maps for Protein intake and incidence of hearing loss; **(F)** Forest maps for Fiber intake and incidence of hearing loss; **(G)** Forest maps for Fish intake and incidence of hearing loss.

In the subgroup analysis of seven studies, no significant association was found between vitamin A and hearing loss (OR = 1.05, 95% CI: 0.980–1.127, *p* = 0.713), with I^2^ of 0%. For retinol (*n* = 5), the OR was 0.839 (95% CI: 0.697–1.008). Despite a significant *p*-value of 0.002, the confidence interval includes 1, indicating no significant inverse association, and high heterogeneity (I^2^ = 76.9%) (Table 2), warranting cautious interpretation (Figure 2).

#### 3.1.2 Vitamin B

The meta-analysis of vitamin B (21 studies) showed the pooled OR of 0.99 (95% CI: 0.957–1.024, *p* = 0.549) with I^2^ of 44.8%, indicating no significant association with hearing loss (Table 2). Egger's test showed no significant small-study bias (p = 0.257), and sensitivity analysis confirmed the robustness of the results (Supplementary material 1).

Subgroup analysis for vitamin B2 (*n* = 4) showed a significant inverse association (OR 0.835, 95% CI: 0.731–0.954, *p* = 0.008, I^2^ = 21.6%). For vitamin B1 (*n* = 4), OR was 0.915 (95% CI: 0.812–1.031, *p* = 0.144, I^2^ = 30.1%), indicating no significant association. Vitamin B3 (*n* = 6) had an OR of 0.992 (95% CI: 0.921–1.069, *p* = 0.834, I^2^ = 58.4%), showing no significant association. For vitamin B6 (*n* = 2), OR was 1.000 (95% CI: 0.928–1.078, *p* = 0.993, I^2^ = 0%), indicating no association. Vitamin B9 (*n* = 3) showed an OR of 0.985 (95% CI: 0.923–1.052, *p* = 0.651, I^2^ = 28.9%), with no significant association. Vitamin B12 (*n* = 2) had an OR of 1.087 (95% CI: 1.001–1.180, *p* = 0.047, I^2^ = 0%), with the CI close to 1, suggesting a weak positive association with hearing loss (Figure 2).

#### 3.1.3 Vitamin C

The meta-analysis of vitamin C (nine studies) showed the pooled OR of 0.988 (95% CI: 0.896–1.088, *p* = 0.804) with I^2^ of 58.7%, indicating no significant association with hearing loss (Table 2, Figure 2). Egger's test did not show significant small-study bias (*p* = 0.964), and sensitivity analysis confirmed the robustness of the results (Supplementary material 1).

#### 3.1.4 Vitamin E

The analysis of vitamin E included five studies, with a pooled OR of 1.019 (95% CI: 0.909–1.142; *p* = 0.747) an I^2^ of 58.3%, indicating no significant association between vitamin E and hearing loss (Table 2, Figure 2). Egger's test did not show significant small-study bias (*p* = 0.529) (Supplementary funnel diagram in Supplementary Figure 7), and sensitivity analysis confirmed the robustness of the results (Supplementary material 1).

#### 3.1.5 Carotenoids

The meta-analysis of carotenoids (13 studies) showed the pooled OR of 0.968 (95% CI: 0.934–1.002, *p* = 0.067) with I^2^ of 5.6%, indicating no significant association between carotenoid intake and hearing loss (Table 2, Figure 2). Egger's test did not show significant small-study bias (*p* = 0.432) (Supplementary funnel diagram in Supplementary Figure 9), and sensitivity analysis confirmed the robustness of the results (Supplementary material 1).

In the subgroup analysis, for unspecified carotenoids (*n* = 6), OR was 1.000 (95% CI: 0.941–1.062, *p* = 0.99), with no heterogeneity (I^2^ = 0%), indicating no significant association. β-carotene (*n* = 6) showed an effect size of 0.932 (95% CI: 0.887–0.980, *p* = 0.006), with an I^2^ of 19.2%, indicating a significant inverse association. α-carotene (*n* = 1) showed an OR of 1.01 (95% CI: 0.929–1.098 *p* = 0.816), indicating no significant association (Figure 2).

Meanwhile, in the analysis of ARHL and dietary nutrients,β-Cryptoxanthin (OR = 0.926) and renieratene (OR = 0.84) exhibited strong protective effects, likely due to their antioxidant capacity in mitigating cochlear oxidative stress. The overall carotenoid pool (OR = 0.928) further supports their role in auditory preservation (Supplementary Table 5).

#### 3.1.6 Carotenoid types

The meta-analysis for carotenoids types included seven studies, with a pooled OR of 0.928 (95% CI: 0.885–0.972, *p* = 0.002) and I^2^ of 63.1%. The CI was below 1, indicating a significant inverse association between carotenoids and hearing loss (Table 2, Figure 2). Egger's test did not show significant small-study bias (*p* = 0.7) (Supplementary funnel diagram in Supplementary Figure 11), and sensitivity analysis confirmed the robustness of the results (Supplementary Figure 12). β-cryptoxanthin (*n* = 2) showed an OR of 0.926 (95% CI: 0.867–0.990, *p* = 0.024) with an I^2^ of 35.1%, indicating a significant inverse association. Lycopene (*n* = 2) had an OR of 0.951 (95% CI: 0.871–1.039 *p* = 0.267), showing no significant association. Lutein (*n* = 2) showed an OR of 0.958 (95% CI: 0.900–1.019, *p* = 0.174), with no heterogeneity (I^2^ = 0%), indicating no significant association (Figure 2).

#### 3.1.7 Minerals

For iron (*n* = 6), the OR was 0.977 (95% CI: 0.902–1.059, *p* = 0.572), indicating no significant association. Potassium (*n* = 7) showed an OR of 1.027 (95% CI: 0.932–1.131, *p* = 0.592) with I^2^ of 49.1%, showing no significant association. Zinc (*n* = 1) had an effect size of 1.16 (95% CI: 0.620–2.170, *p* = 0.642), indicating no significant association. Phosphorus (*n* = 5) showed an OR of 0.953 (95% CI: 0.851–1.068, *p* = 0.407) with an I^2^ of 18.3%, indicating no significant association. Ash (*n* = 5) showed an OR of 0.975 (95% CI: 0.834–1.140, *p* = 0.750) with I^2^ of 28.5%, indicating no significant correlation. Sodium (*n* = 5) showed an OR of 0.899 (95% CI: 0.780–1.037, *p* = 0.144) with I^2^ of 15.5%, showing no significant correlation (Table 2). Egger's test did not show significant small-study bias (*p* = 0.717), and sensitivity analysis confirmed the robustness of the results (Supplementary material 1).

### 3.2 Macronutrients

#### 3.2.1 Fat

In the overall analysis of fat intake, 26 studies were included, yielding a pooled OR of 0.961 (95% CI: 0.908–1.018, *p* = 0.174) and I^2^ of 56.8%. This indicates that there is no significant association between fat intake and hearing loss (Table 2, Figure 2). Egger's test did not reveal significant small-study bias (*p* = 0.213), and the results of the sensitivity analysis were robust (Supplementary material 1).

In subgroup analyses, when excluding fatty acids (*n* = 9), the pooled OR was 0.92 (95% CI: 0.85–0.991, *p* = 0.028), with I^2^ of 64.9%. The CI was < 1, indicating a significant negative association. Saturated fat (*n* = 5) yielded a pooled OR of 1.084 (95% CI: 0.957–1.229, *p* = 0.205), showing no significant association. Trans fat (*n* = 3) had a pooled OR of 1.209 (95% CI: 0.788–1.855, *p* = 0.385) with I^2^ of 81.5%, also showing no significant association. For studies where fat was not further classified (*n* = 9), the pooled OR was 0.918 (95% CI: 0.850–0.991, *p* = 0.028), with no heterogeneity (I^2^ = 0%). The CI being < 1 indicates a significant negative association (Figure 2).

#### 3.2.2 Protein

Meta-analysis of protein intake (9 studies) showed a pooled OR of 0.87 (95% CI: 0.806–0.939, *p* = 0.000) with no heterogeneity (I^2^ = 0%). The CI being < 1 suggests a significant negative association between protein intake and hearing loss (Table 2, Figure 2). Egger's test did not show significant small-study bias (*p* = 0.49), and the sensitivity analysis indicated robust results (Supplementary material 1).

While in the analysis of ARHL and dietary nutrients, High protein intake correlated with a 14% lower ARHL risk (OR = 0.858), potentially via anti-inflammatory pathways and endothelial health (Supplementary Table 5).

#### 3.2.3 Fiber

Analysis of fiber intake (10 studies) showed a pooled OR of 0.923 (95% CI: 0.854–0.998, *p* = 0.044) with no heterogeneity (I^2^ = 0%). The CI being < 1 indicates a significant negative association between fiber intake and hearing loss (Table 2, Figure 2). Egger's test did not show significant small-study bias (*p* = 0.479), and the sensitivity analysis indicated robust results (Supplementary material 1).

#### 3.2.4 Carbohydrates

The meta-analysis of carbohydrate intake included 10 studies, with a pooled OR of 0.901 (95% CI: 0.781–1.040, *p* = 0.153) and I^2^ of 57.2%. This indicates no significant association between carbohydrate intake and hearing loss (Table 2). Egger's test did not show significant small-study bias (*p* = 0.632), and the sensitivity analysis indicated robust results (Supplementary material 1). Analysis of sugar (carbohydrates) intake (nine studies) yielded a pooled OR of 1.087 (95% CI: 0.996–1.187, *p* = 0.020) with moderate heterogeneity (I^2^ = 55.9%). Despite a significant *p*-value, the confidence interval includes 1, indicating no association between sugar intake and hearing loss (Table 2). Egger's test revealed significant small-study bias (*p* = 0.015), and the sensitivity analysis indicated robust results. After adjustment using the Trim and Fill method (*n* = 11), the pooled OR was 1.065 (95% CI: 0.974–1.164, *p* = 0.168), with I^2^ of 45.3%, indicating no significant association between sugar intake and hearing loss (Supplementary material 1).

### 3.3 Beverages

#### 3.3.1 Coffee

Analysis of coffee intake (10 studies) yielded a pooled OR of 0.886 (95% CI: 0.764–1.026, *p* = 0.024) with moderate heterogeneity (I^2^ = 53%), indicating no significant association with hearing loss (Table 2). Egger's test did not reveal significant small-study bias (*p* = 0.791), and the results of the sensitivity analysis were robust (Supplementary material 1).

#### 3.3.2 Tea

Meta-analysis of tea intake (6 studies) showed a pooled OR of 1.065 (95% CI: 0.895–1.268, *p* = 0.080, I^2^ = 49.1%), indicating no significant association with hearing loss (Table 2). Egger's test did not show significant small-study bias (*p* = 0.756), and the sensitivity analysis indicated robust results (Supplementary material 1).

#### 3.3.3 Alcohol

Meta-analysis of alcohol consumption (nine studies) showed a pooled OR of 0.938 (95% CI: 0.762–1.155, *p* = 0.549, I^2^ = 93.7%), indicating no association with hearing loss (Table 2). Egger's test did not show significant small-study bias (*p* = 0.685), and the sensitivity analysis indicated robust results (Supplementary material 1).

### 3.4 Specific foods

#### 3.4.1 Fish

Meta-analysis of fish consumption (four studies) showed a pooled OR of 0.868 (95% CI: 0.759–0.994, *p* = 0.047, I^2^ = 0%), indicating a significant negative association with hearing loss (Table 2). Egger's test did not reveal significant small-study bias (*p* = 0.088), and the sensitivity analysis indicated robust results (Supplementary material 1).

### 3.5 Key findings in ARHL

#### 3.5.1 Carotenoids and antioxidants

The significant protective effect of β-cryptoxanthin (OR = 0.926) and overall carotenoids (OR = 0.928) in ARHL aligns with their role in mitigating age-related oxidative stress in the cochlea. This mechanism is less prominent in Non-ARHL subtypes (e.g., noise-induced loss), where acute oxidative damage dominates (Supplementary Table 5).

#### 3.5.2 Protein and tea

Protein's strong association with ARHL reduction (OR = 0.858) may reflect its support for cochlear mitochondrial function and vascular health, critical in aging auditory systems. Tea's protective effect (OR = 0.887) likely stems from polyphenols targeting chronic inflammation, a hallmark of ARHL (Supplementary Table 5).

#### 3.5.3 Contrasting results in non-ARHL

##### 3.5.3.1 Carbohydrates' paradoxical association

The inverse link between Carbohydrates (sugar) and ARHL (OR = 0.858) was not observed in Non-ARHL subgroups, suggesting this finding may be specific to age-related metabolic contexts (e.g., glucose utilization in elderly populations) (Supplementary Table 6).

##### 3.5.3.2 Limited protective signals

No nutrients showed consistent protection in Non-ARHL, emphasizing the unique pathophysiology of ARHL. For example, magnesium (OR = 1.07 in ARHL) had neutral effects in noise-induced loss studies (Supplementary Table 6).

## 4 Discussion

This meta-analysis reveals the potential impact of specific dietary and nutritional factors on hearing loss. Our findings indicate significant negative associations between the intake of vitamin B2, β-carotene, carotenoids, β-cryptoxanthin, fat, fiber, and fish, and the risk of hearing loss. In contrast, the intake of minerals, carbohydrates, vitamins A, C, and E, carotene, tea, coffee, alcohol, and sugar did not show significant associations. These results suggest that, while dietary habits may be linked to hearing health, the effects of different nutrients may vary depending on their biological functions and intake levels.

The mechanisms by which dietary and nutritional factors influence hearing health are complex and multifaceted. Previous studies have identified cochlear microvascular disease, dyslipidemia, oxidative stress imbalance, and alterations in insulin signaling as potential mechanisms through which diet, obesity, and metabolic diseases contribute to cochlear damage and hearing impairment (6). Overall, the protective dietary and nutritional patterns we identified may involve multiple aspects such as antioxidant defense, inflammation regulation, metabolic support, vascular health maintenance, and gene-environment interactions.

### 4.1 Vitamin B2 (riboflavin) and hearing loss

Oxidative stress is a key factor contributing to hearing loss. The cochlear cells of the inner ear are highly metabolically active and are prone to generating large amounts of reactive oxygen species (ROS). If these ROS are not effectively neutralized, they can lead to lipid peroxidation of cell membranes, DNA damage, protein denaturation, and ultimately apoptosis and necrosis of cochlear hair cells. Vitamin B2 (riboflavin) acts as a crucial coenzyme in the body, participating in energy metabolism and the antioxidant defense system. Its role in the inner ear may be associated with hearing loss through two pathways. First, riboflavin supports antioxidant defense. It serves as a coenzyme for glutathione reductase, helping to maintain the activity of glutathione, which neutralizes free radicals and reduces oxidative damage to cochlear hair cells. Oxidative stress is closely related to the pathological processes of hearing loss, and riboflavin deficiency may increase lipid peroxidation in cell membranes, accelerating the degeneration of cochlear cells (51). Second, riboflavin supports mitochondrial function. As an essential vitamin for mitochondrial function, its deficiency may lead to disruptions in cellular energy metabolism, impairing the normal function and survival of cochlear hair cells and increasing the risk of noise-induced hearing loss (52).

### 4.2 Carotenoids and hearing loss

Carotenoids are plant pigments with antioxidant activity, widely present in plant-based foods, including β-carotene, β-cryptoxanthin, lutein, and zeaxanthin. Prolonged exposure to noise and other environmental stressors leads to the generation of excessive free radicals within the cochlea. Carotenoids, particularly β-carotene, can directly scavenge ROS in the hair cells of the inner ear, thereby inhibiting oxidative stress-induced DNA damage and lipid peroxidation. Additionally, carotenoids reduce inner ear inflammation by suppressing the release of pro-inflammatory cytokines such as TNF-α and IL-1β. Chronic inflammation is a potential pathogenic factor in both age-related and noise-induced hearing loss, and the anti-inflammatory properties of carotenoids may slow this degenerative process (52). Carotenoids also improve microcirculation, enhancing endothelial function and increasing nutrient supply to the inner ear hair cells, which supports the maintenance of cochlear function (53). From a metabolic perspective, β-carotene and β-cryptoxanthin can increase intracellular glutathione levels, modulate cytokine production, and alter lipid metabolism (54). Moreover, autophagy and apoptosis of hair cells in the inner ear are crucial for maintaining cochlear health. These dietary antioxidants or anti-inflammatory nutrients may influence hearing health by regulating the expression of autophagy-related genes (e.g., LC3-II, Beclin-1) and inhibiting key proteins in apoptotic signaling pathways (e.g., caspase-3, Bax), thus preventing cell apoptosis (55). Research indicates that antioxidants can protect inner ear cells by reducing autophagic cell death (56).

### 4.3 Protein and hearing loss

Hair cells in the inner ear are essential for auditory perception, as they convert mechanical vibrations of sound into signals transmitted to the auditory nerve (57). Proteins play a critical role in cellular structure, signal transduction, and the regulation of ion channels (58). As integral components of cell membranes, cytoskeletons, and enzymes, proteins are crucial for maintaining cochlear fluid balance and ion exchange, both of which are vital for auditory function (59). Inner ear cells require proteins for the repair of damaged structures, the synthesis of essential enzymes, and the transmission of signaling molecules. Protein deficiency may increase cellular vulnerability, reducing the ability to resist environmental stress. Additionally, proteins are involved in the body's antioxidant mechanisms, helping to combat oxidative stress in the auditory system. Oxidative stress can damage hair cells, leading to hearing loss (60). Certain amino acids (the building blocks of proteins), such as glutamate and cysteine, are precursors for the synthesis of antioxidants, which help protect the inner ear from free radical damage.

### 4.4 Fiber and hearing loss

Dietary fiber, an important component of a healthy diet, may protect hearing by improving metabolism and reducing inflammation. Dietary fiber promotes the production of beneficial short-chain fatty acids, such as butyrate, regulates the gut microbiota, and enhances systemic immune function (61). A healthy immune system is crucial for protecting the cochlea from chronic inflammation and oxidative damage (62). Additionally, a high-fiber diet helps maintain metabolic health, reducing the risk of hyperlipidemia and hyperglycemia, thereby indirectly decreasing the likelihood of hearing loss associated with these metabolic issues (63).

### 4.5 Fat and hearing loss

The relationship between fat intake and hearing loss is complex, depending on the type of fat consumed. Monounsaturated fatty acids and polyunsaturated fatty acids (PUFA) are considered beneficial, while saturated fats and trans fats are deemed unhealthy. However, the current lack of extensive research on trans fats and saturated fats has resulted in insufficiently convincing data to confirm the impact of these fats on the risk of hearing loss. Inadequate blood supply to the cochlea can impair the maintenance of the cochlear electrical potential, ion transport, endolymph balance, and the integrity of the stria vascularis-blood labyrinth barrier, potentially leading to hypoxic-ischemic injury to hair cells and subsequent hearing loss (64). Omega-3 fatty acids, particularly long-chain omega-3 PUFA, have been shown to improve vascular reactivity and endothelial function; positively influence plasma lipids, triglycerides, and blood pressure; and prevent thrombosis and inflammation. Long-chain omega-3 PUFA may affect membrane structure, regulate ion channels and electrophysiological responses to ischemic stress, modulate gene expression, reduce pro-inflammatory or pro-thrombotic eicosanoids derived from arachidonic acid, and increase eicosanoids derived from omega-3 PUFA (65). A study found that consumption of fish rich in omega-3 fatty acids was associated with a 42% reduction in the incidence of age-related hearing loss after a 5-year follow-up (30). Our analysis also found that fish, particularly deep-sea fish rich in omega-3 fatty acids, had a protective effect on hearing, likely due to the anti-inflammatory and antioxidant properties of omega-3 fatty acids. Eicosapentaenoic acid and docosahexaenoic acid in fish may reduce the generation of inflammatory mediators, improve cochlear microcirculation, and promote the repair of cochlear hair cells, potentially lowering the risk of noise-induced and age-related hearing loss (66). Furthermore, omega-3 fatty acids are known for their cardiovascular protective effects, as they can improve cochlear blood supply, increasing oxygen and nutrient delivery to inner ear tissues, thereby maintaining stable auditory function (65). In contrast, excessive intake of saturated and trans fats may exacerbate systemic inflammation and vascular damage, potentially reducing blood supply to the inner ear and increasing the risk of hearing loss (67). These findings are consistent with our analysis. Our findings suggest that specific dietary adjustments may contribute to hearing protection. In particular, increasing the intake of protein, carotenoids, fiber, and deep-sea fish rich in unsaturated fatty acids may play a positive role in hearing protection strategies. Therefore, simple dietary interventions could be an economical and feasible approach to preventing hearing loss. Moreover, the characteristics of the studies we included suggest that hearing loss has a more significant impact on the quality of life in the elderly population. However, it is important to note that the intake of a single nutrient may not be sufficient to significantly reduce the risk of hearing loss. Comprehensive and healthy dietary patterns, such as the Mediterranean diet or a diet high in antioxidants, may be more effective. This was reflected in our systematic review, but due to insufficient sample sizes, no meta-analysis for dietary pattern was conducted. Given the broader impacts of diet on health, dietary recommendations for hearing protection should be incorporated into broader nutritional and health guidelines.

We only included observational studies, and while these studies provide valuable correlational data, causality has not been established. Observational studies are inherently limited by confounding and measurement biases. Additionally, the heterogeneity observed in some included studies, likely stemming from variations in dietary assessment methods, hearing measurement tools, age, and types of hearing loss, poses another challenge. The impact of dietary factors on hearing health is influenced by both the quantity and duration of intake. Although our analysis utilized the extreme intake values (highest consumption) from each study, which lends credibility to the findings, it does not provide a comprehensive view. A dose-response analysis is essential when sufficient studies are available, which will be a key focus for future research. This study highlights the need to further explore the relationship between diet and hearing health. Future research should prioritize large-scale, prospective randomized controlled trials to confirm the effects of specific diets on hearing protection. Accurate and standardized tools for assessing food intake and more reliable diagnostic methods for hearing loss, beyond simple measurements, are needed. More rigorous control of covariates and confounding factors is also essential. Furthermore, studies should examine the relationship between diet and hearing loss across different age groups, ethnicities, and genetic backgrounds to determine whether individualized dietary recommendations are warranted. Prioritize diets rich in carotenoids (citrus, carrots), protein (lean meats, legumes), and tea. Public health guidelines should tailor recommendations to older adults. On-ARHL Management: Dietary interventions may require etiology-specific approaches (e.g., omega-3 for noise-induced loss), warranting targeted studies. Furthermore, we stress that the individual differences in nutritional metabolism influenced by genetic polymorphisms and environmental factors will affect the hearing outcomes.

## 5 Conclusion

To the best of our knowledge, this study is the first to conduct a comprehensive meta-analysis on the impact of dietary factors and nutrition types on hearing, following a thorough review of the existing literature. By synthesizing available observational studies, this meta-analysis provides a comprehensive perspective on the relationship between dietary nutritional factors and hearing loss. We were able to identify and quantify the associations between dietary factors and hearing loss, revealing the combined effects of various dietary factors. Several nutrients with potential protective effects were identified, while no strong evidence was found to support dietary habits posing a risk for hearing loss. The heterogeneity of the findings and the limitations inherent to observational study designs highlight the need for further research to confirm these results. Based on the published literature methods (68), we combined studies of different designs. However, the sensitivity analysis indicated that the core conclusions remained unchanged after excluding cross-sectional data. This robustness might stem from the following two points: Firstly, hearing loss is a chronic degenerative disease, and dietary exposures in cross-sectional studies mostly reflect long-term patterns; Secondly, the average follow-up period of longitudinal studies is relatively short, which may underestimate the impact of lifelong diet. In the future, it is necessary to combine Mendelian randomization studies to verify causal hypotheses and avoid the confounding factors and potential causal inversion that observational studies cannot avoid. Future studies and clinical practice should consider the potential benefits and risks of diet on hearing health and incorporate them into comprehensive hearing loss prevention strategies.

## Data Availability

The original contributions presented in the study are included in the article/Supplementary material, further inquiries can be directed to the corresponding author.
