# Supplementary material for: Protective effects of dietary nutrients on hearing loss: a systematic review and meta-analysis
Source: Front Nutr. 2025 May 9;12:1528771. doi: 10.3389/fnut.2025.1528771 (PMC12100664; doi:10.3389/fnut.2025.1528771)
Supplement: Supplementary file 1 [file Data_Sheet_1.zip › 补充文件/Supplement Material 1 all funnel plot and sensitivity analysis.docx]

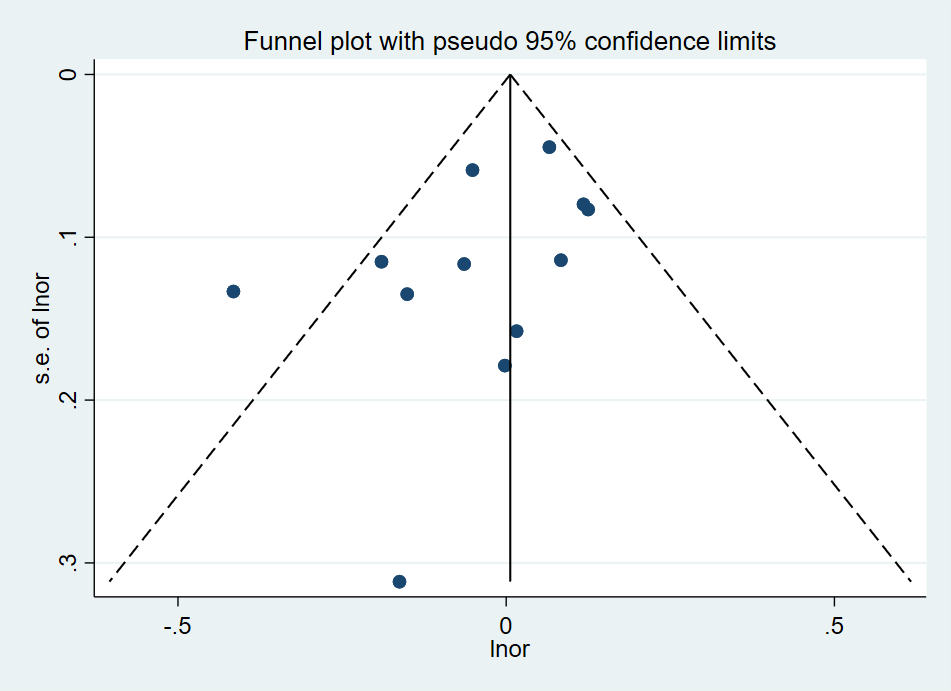


Figure S1: Funnel plot for the association between Vitamin A intake and the incidence of hearing loss.


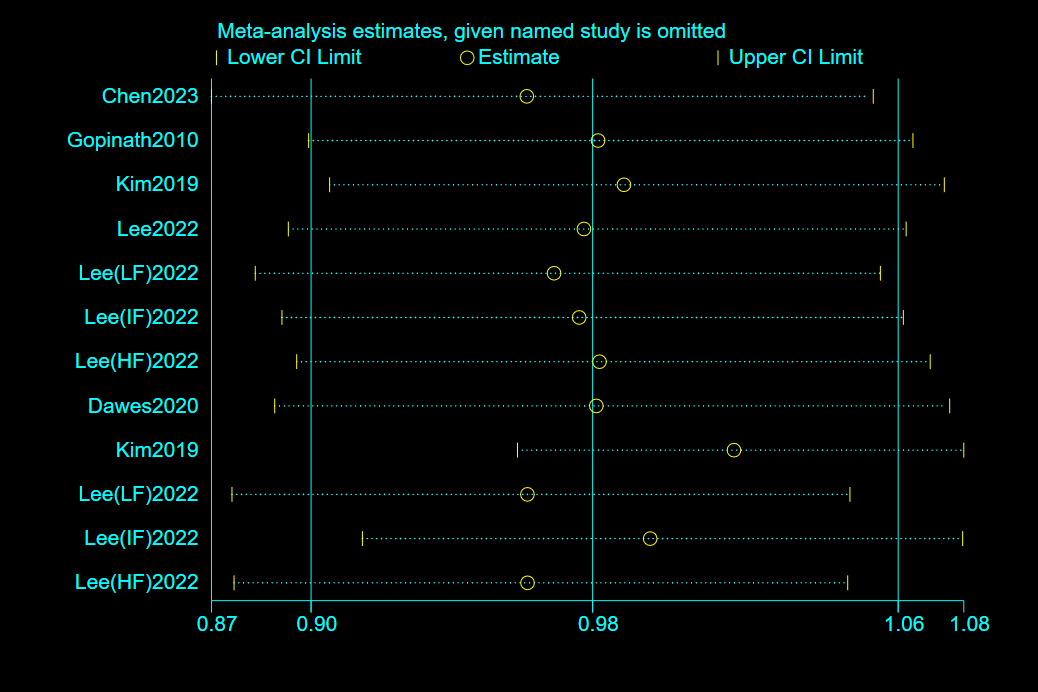


Figure S2: Sensitivity analysis for the effect of Vitamin A intake on the incidence of hearing loss.


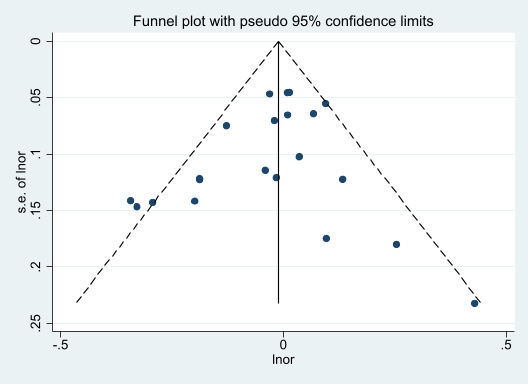


Figure S3: Funnel plot for the association between Vitamin B intake and the incidence of hearing loss.


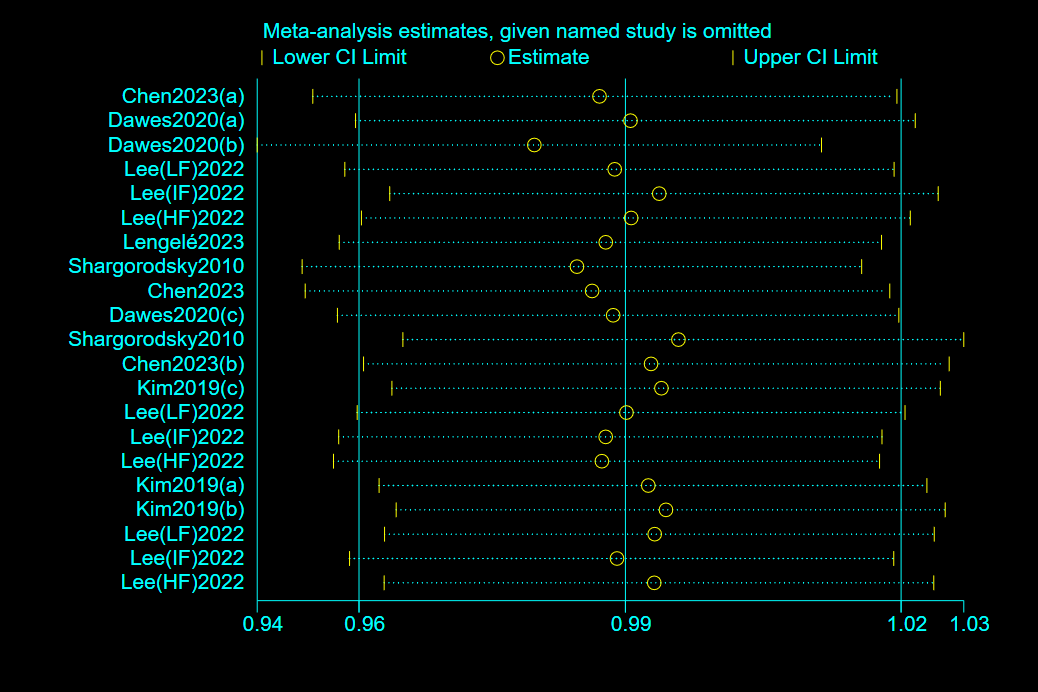


Figure S4: Sensitivity analysis for the effect of Vitamin B intake on the incidence of hearing loss.


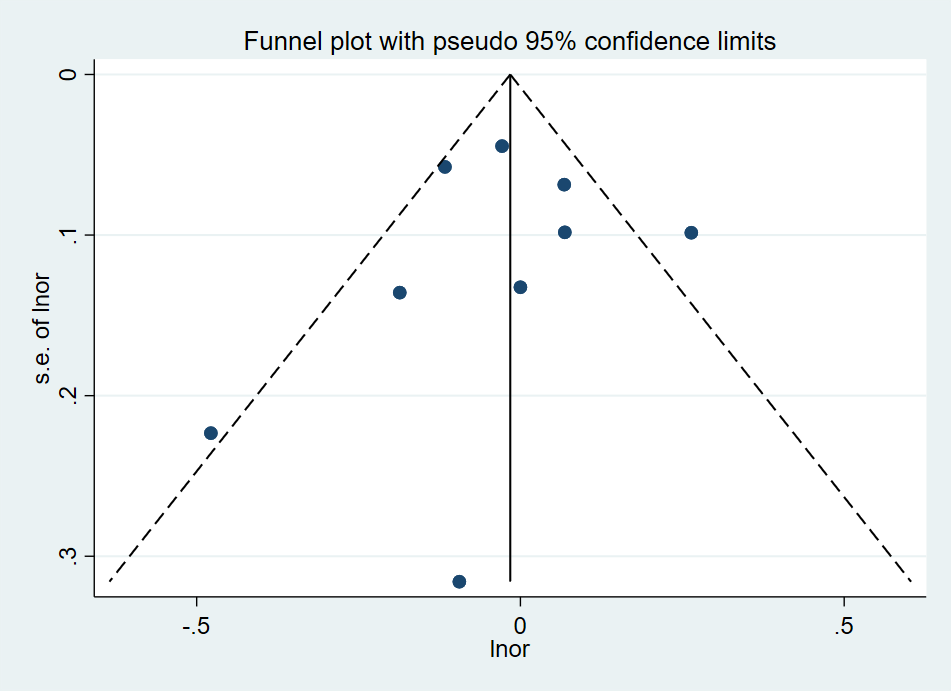


Figure S5: Funnel plot for the association between Vitamin C intake and the incidence of hearing loss.


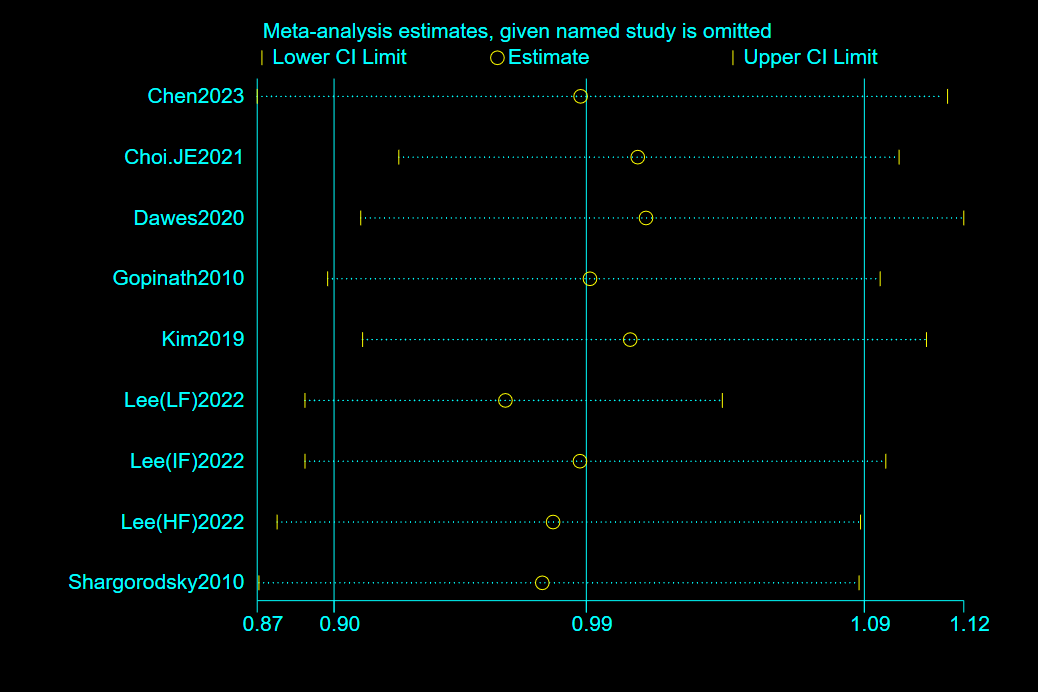


Figure S6: Sensitivity analysis for the effect of Vitamin C intake on the incidence of hearing loss.


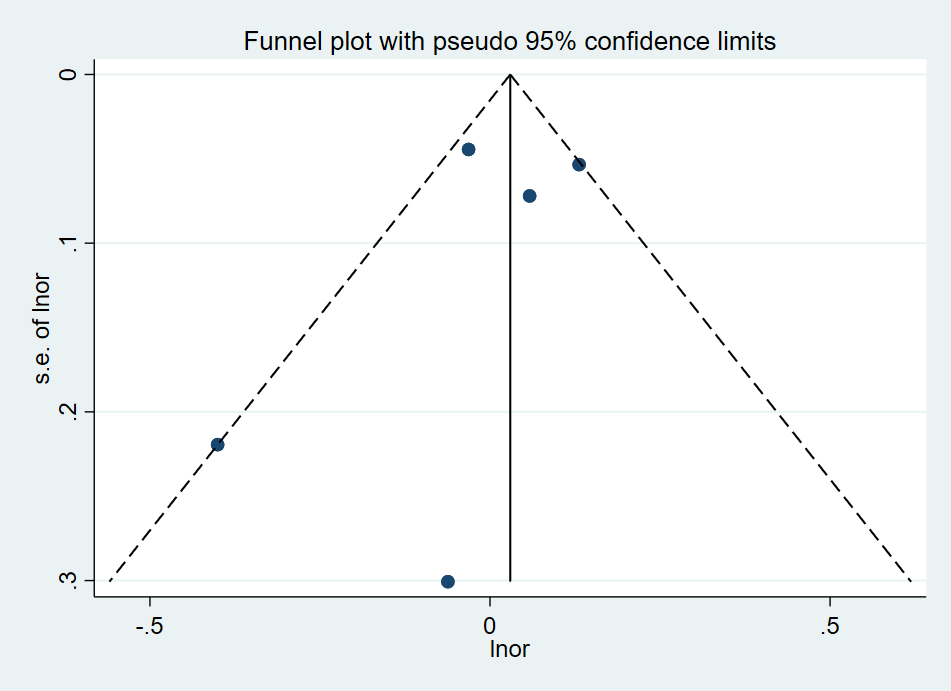


Figure S7: Funnel plot for the association between Vitamin E intake and the incidence of hearing loss.


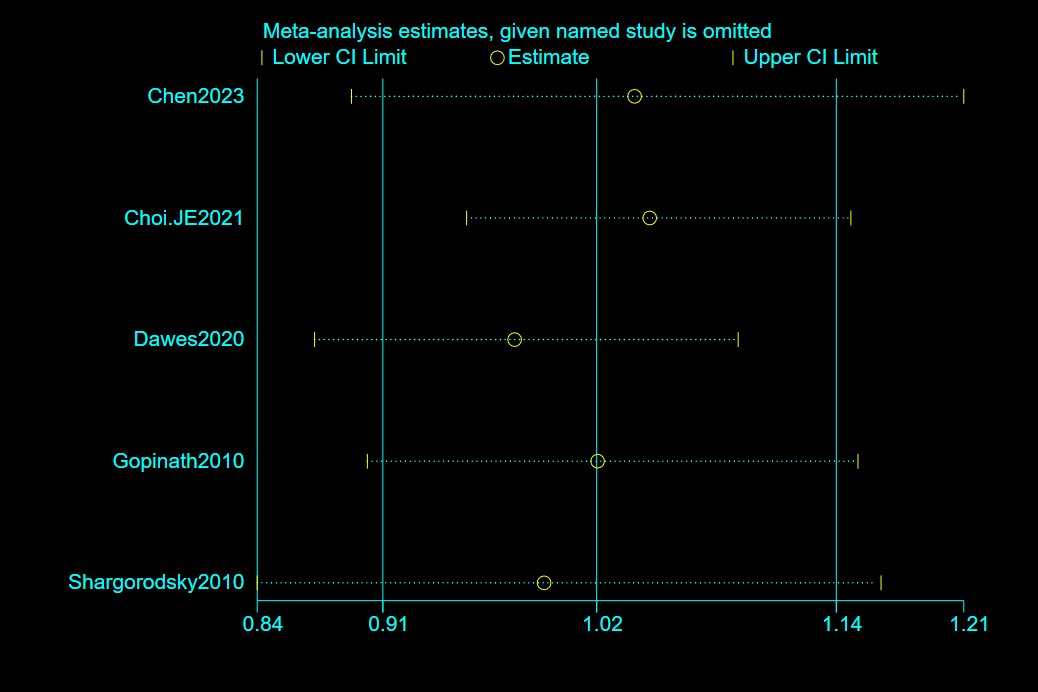


Figure S8: Sensitivity analysis for the effect of Vitamin E intake on the incidence of hearing loss.
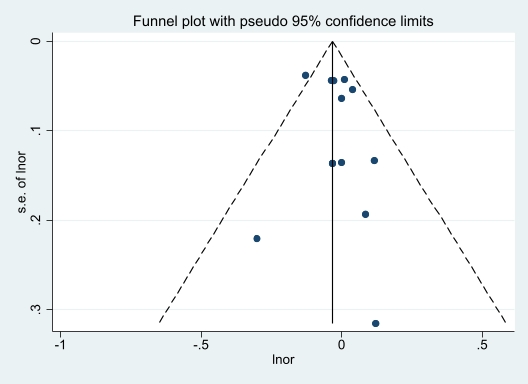


Figure S9: Funnel plot for the association between Carotenoids intake and the incidence of hearing loss.


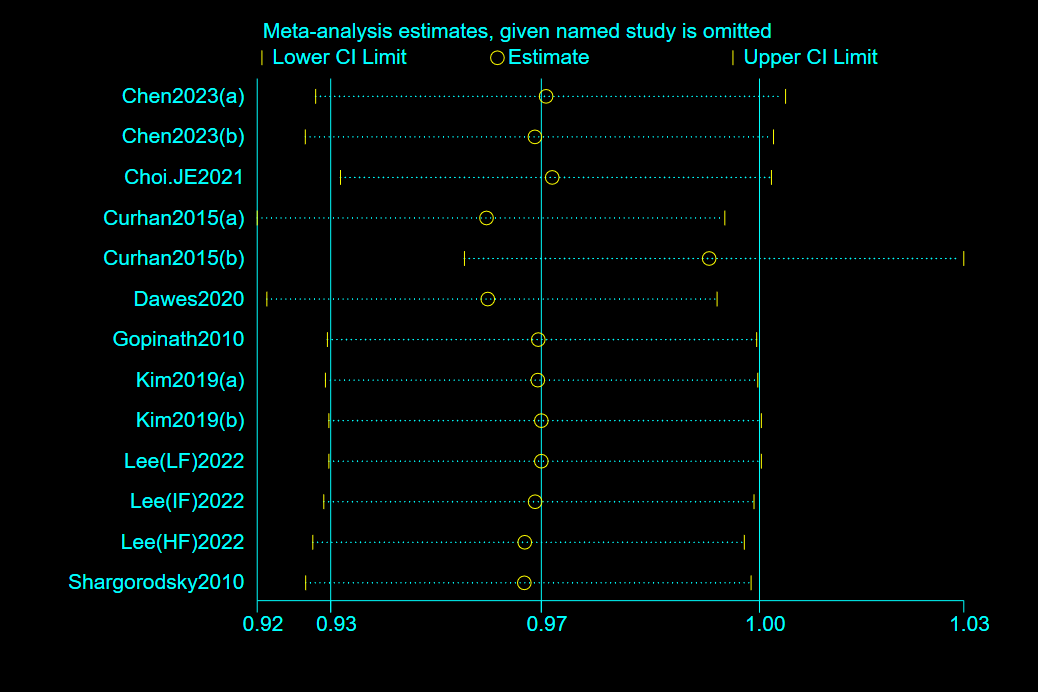


Figure S10: Sensitivity analysis for the effect of Carotenoids intake on the incidence of hearing loss.


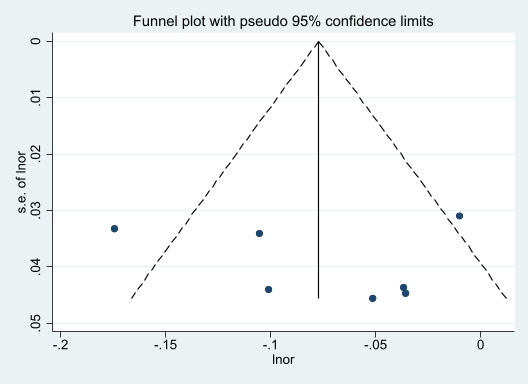


Figure S11: Funnel plot for the association between different types of Carotenoids intake and the incidence of hearingloss
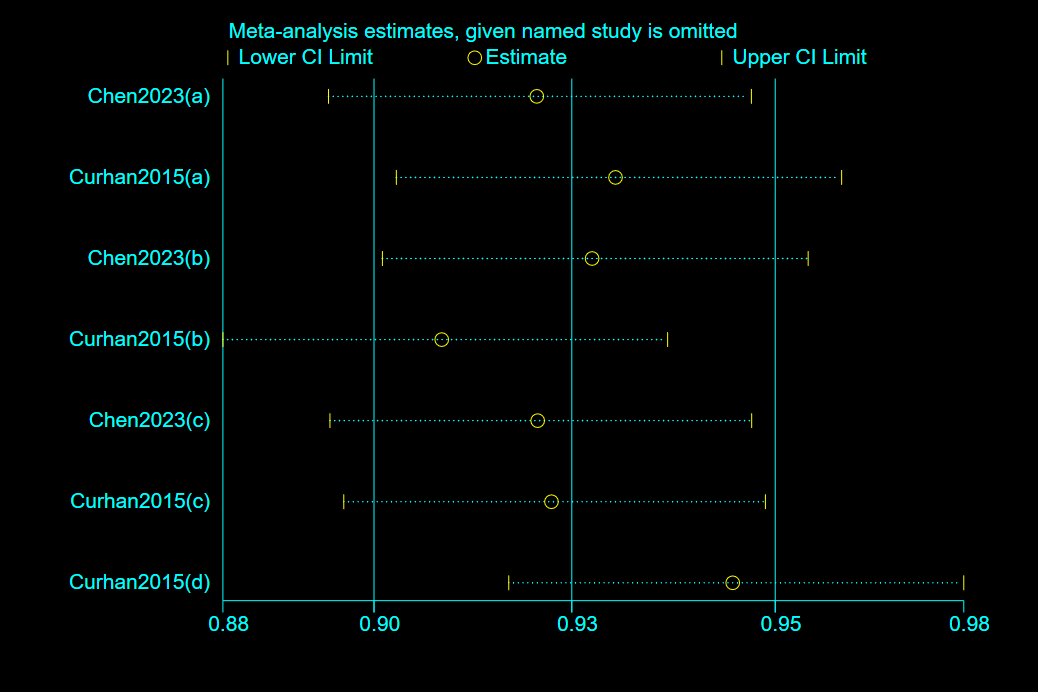


Figure S12: Sensitivity analysis for the effect of different types of Carotenoids intake on the incidence of hearing loss.


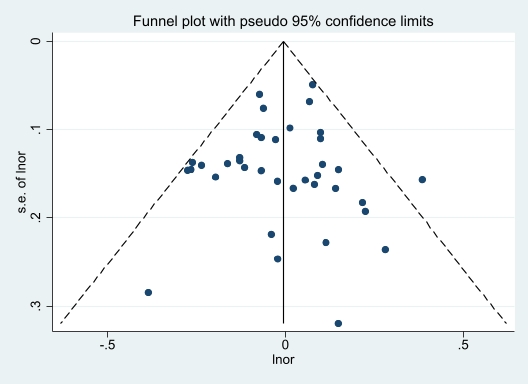


Figure S13: Funnel plot for the association between Minerals intake and the incidence of hearing loss.


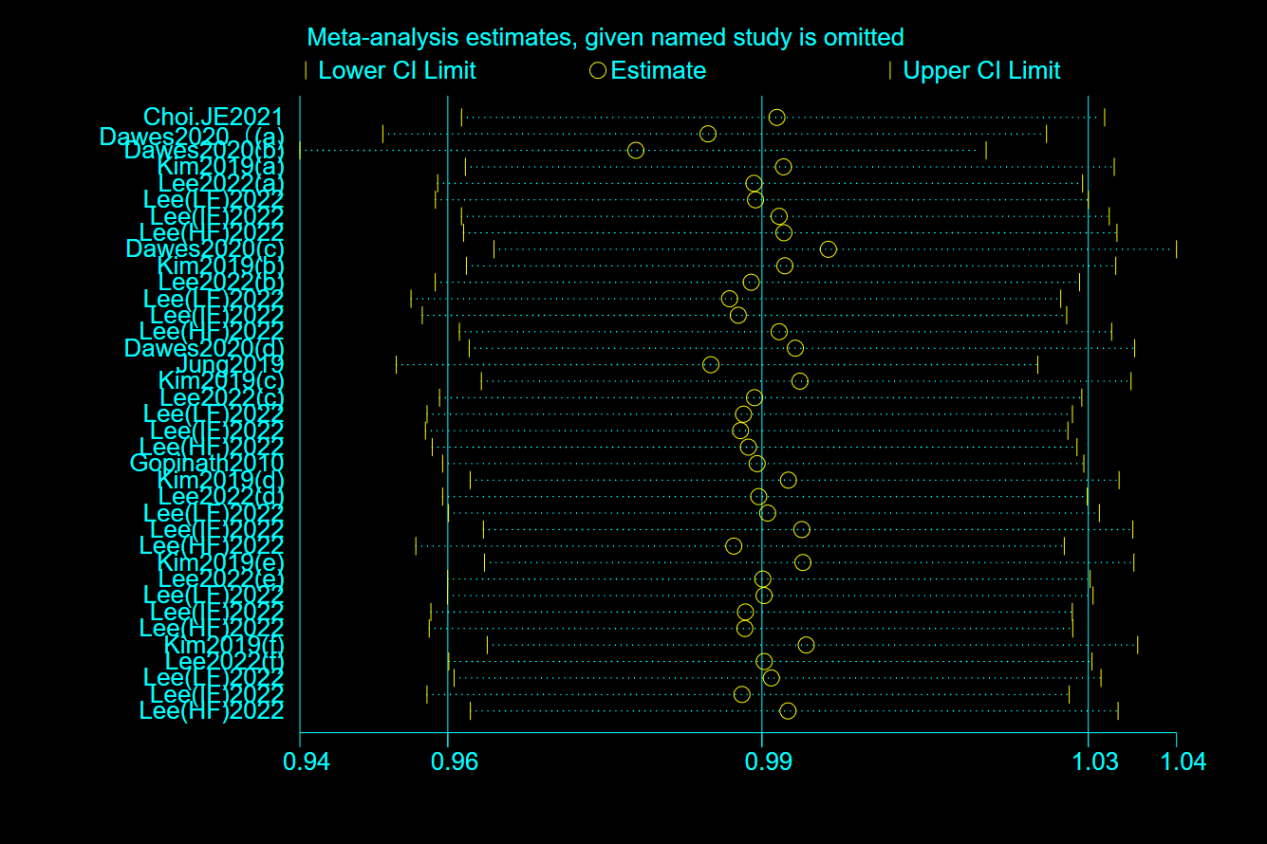


Figure S14: Sensitivity analysis for the effect of Minerals intake on the incidence of hearing loss.


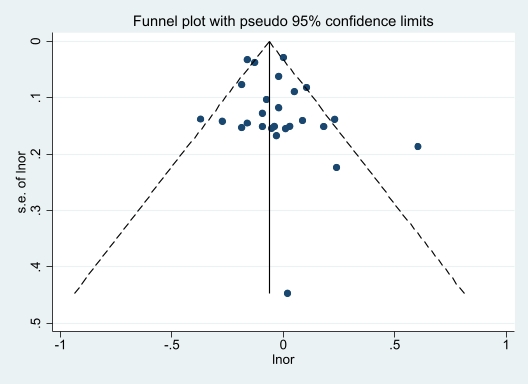


Figure S15: Funnel plot for the association between Fat intake and the incidence of hearing loss.


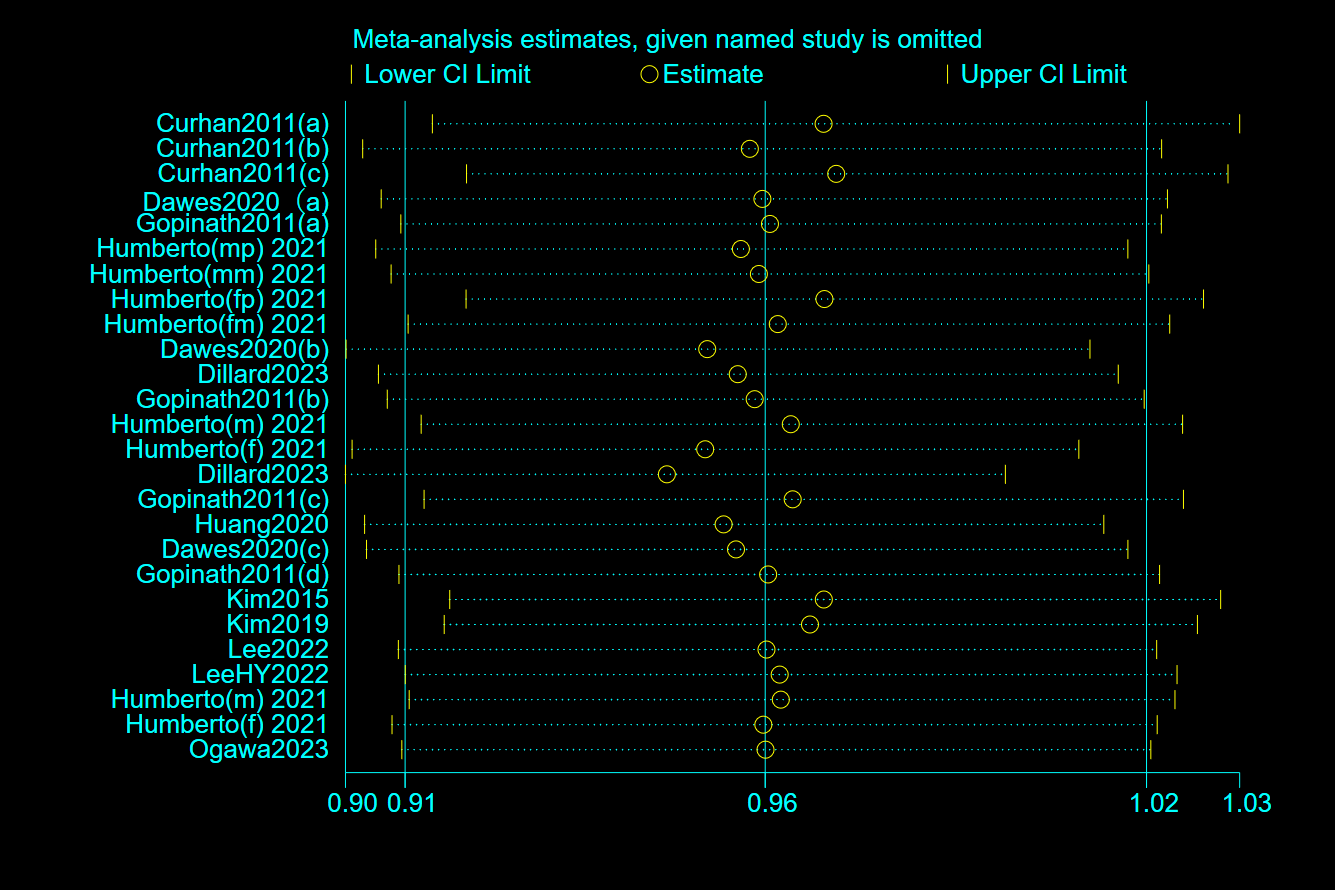


Figure S16: Sensitivity analysis for the effect of Fat intake on the incidence of hearing loss.


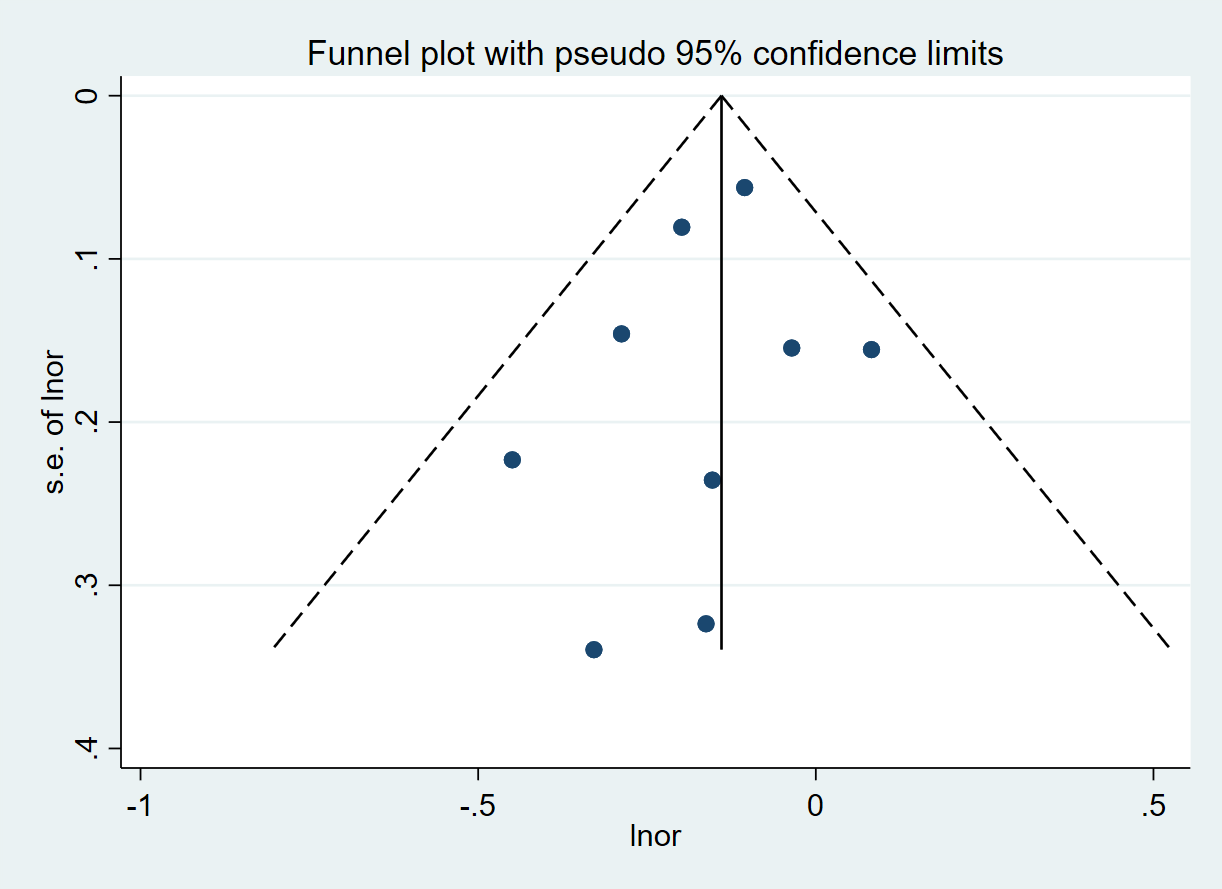


Figure S17: Funnel plot for the association between Protein intake and the incidence of hearing loss.
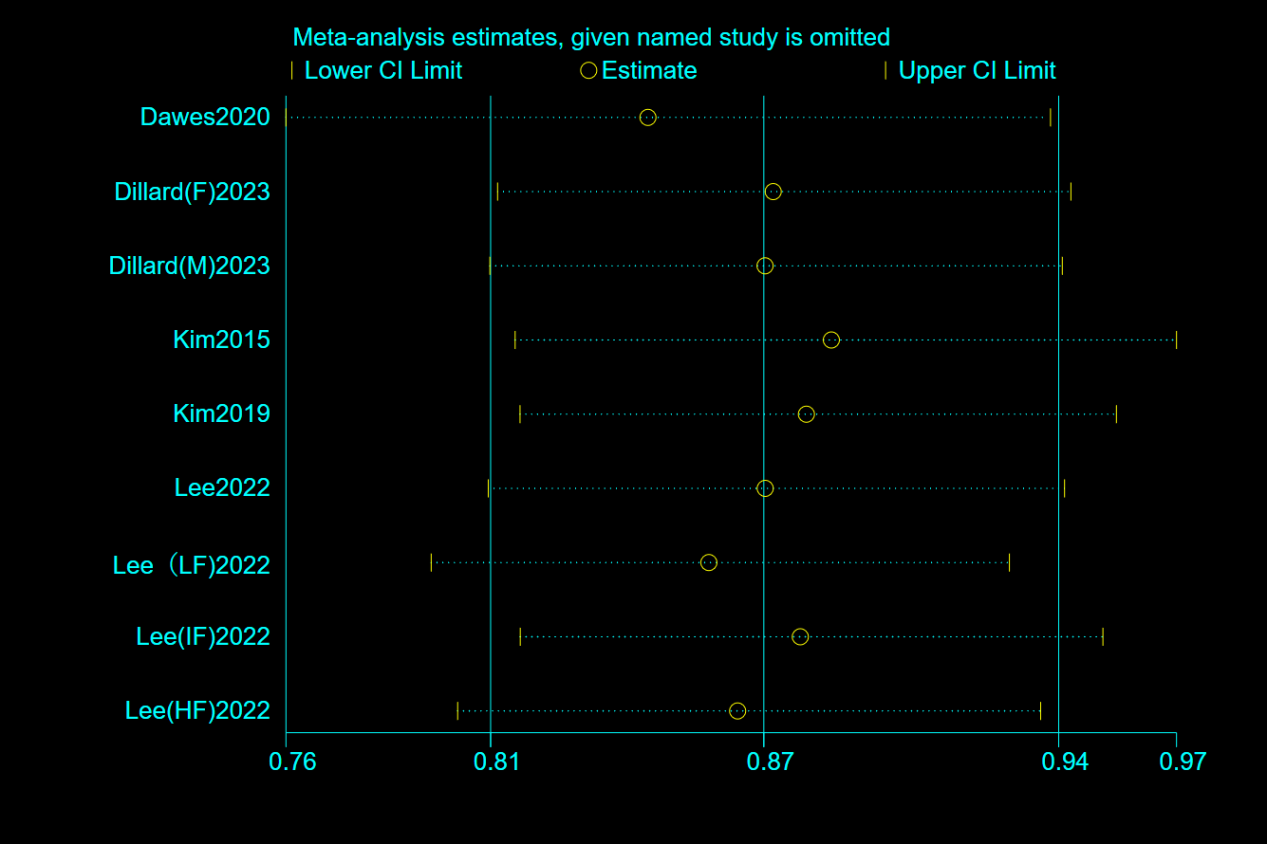


Figure S18: Sensitivity analysis for the effect of Protein intake on the incidence of hearing loss.


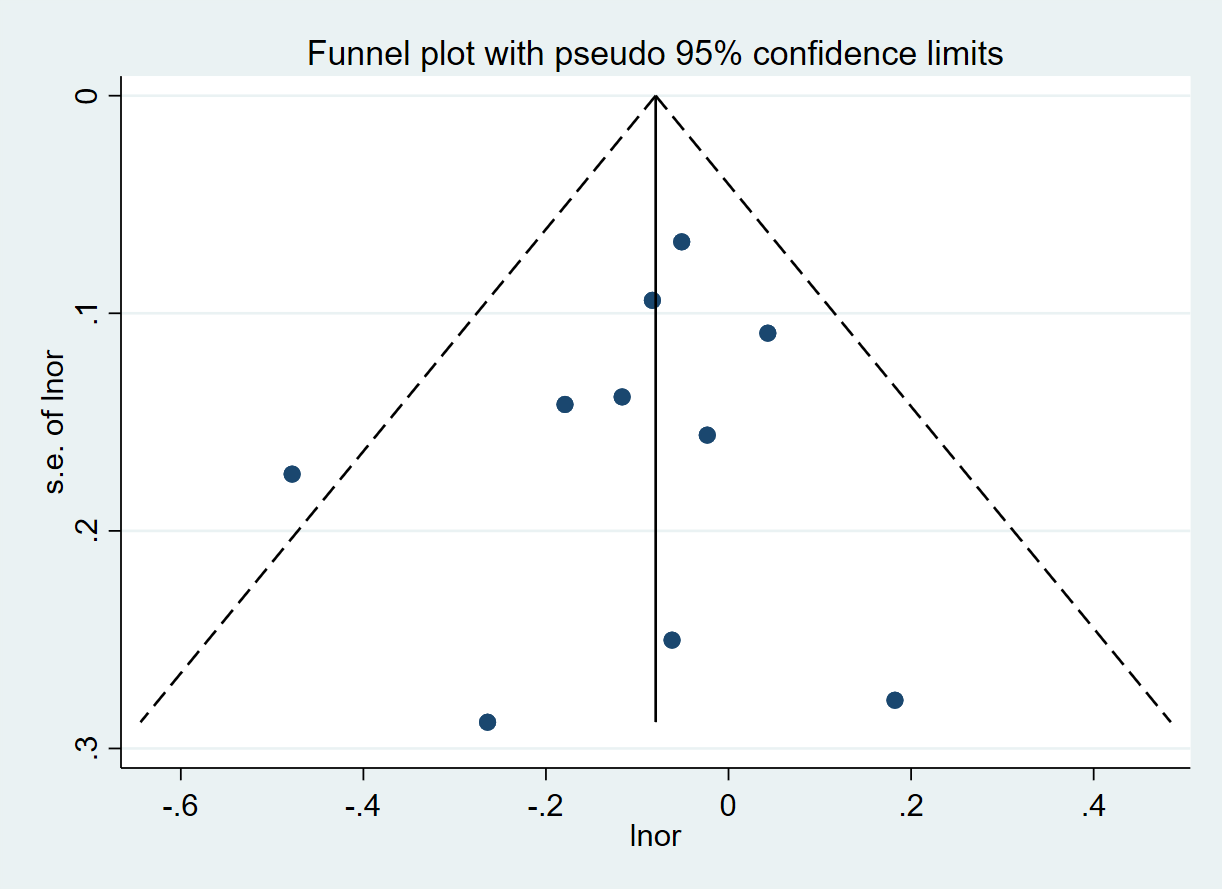


Figure S19: Funnel plot for the association between Fiber intake and the incidence of hearing loss.
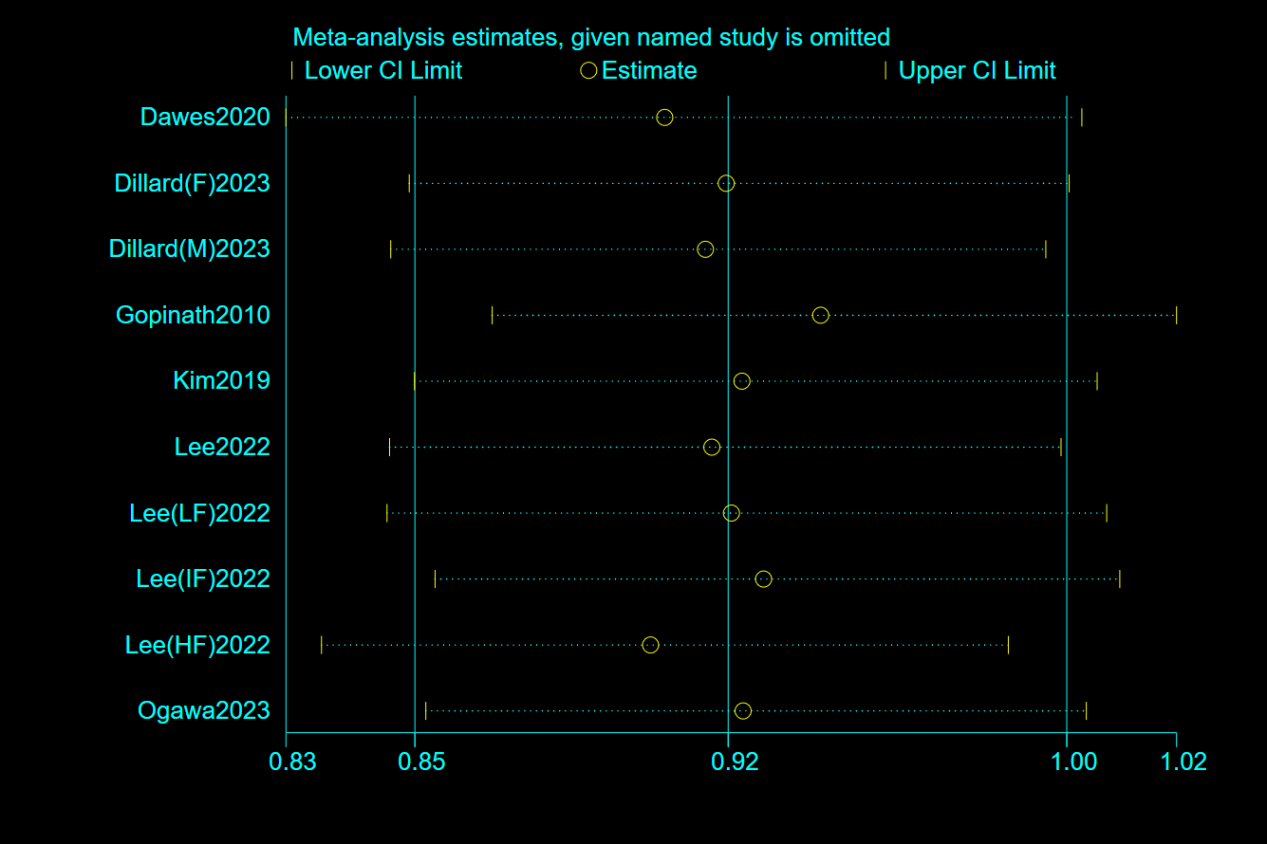


Figure S20: Sensitivity analysis for the effect of Fiber intake on the incidence of hearing loss.
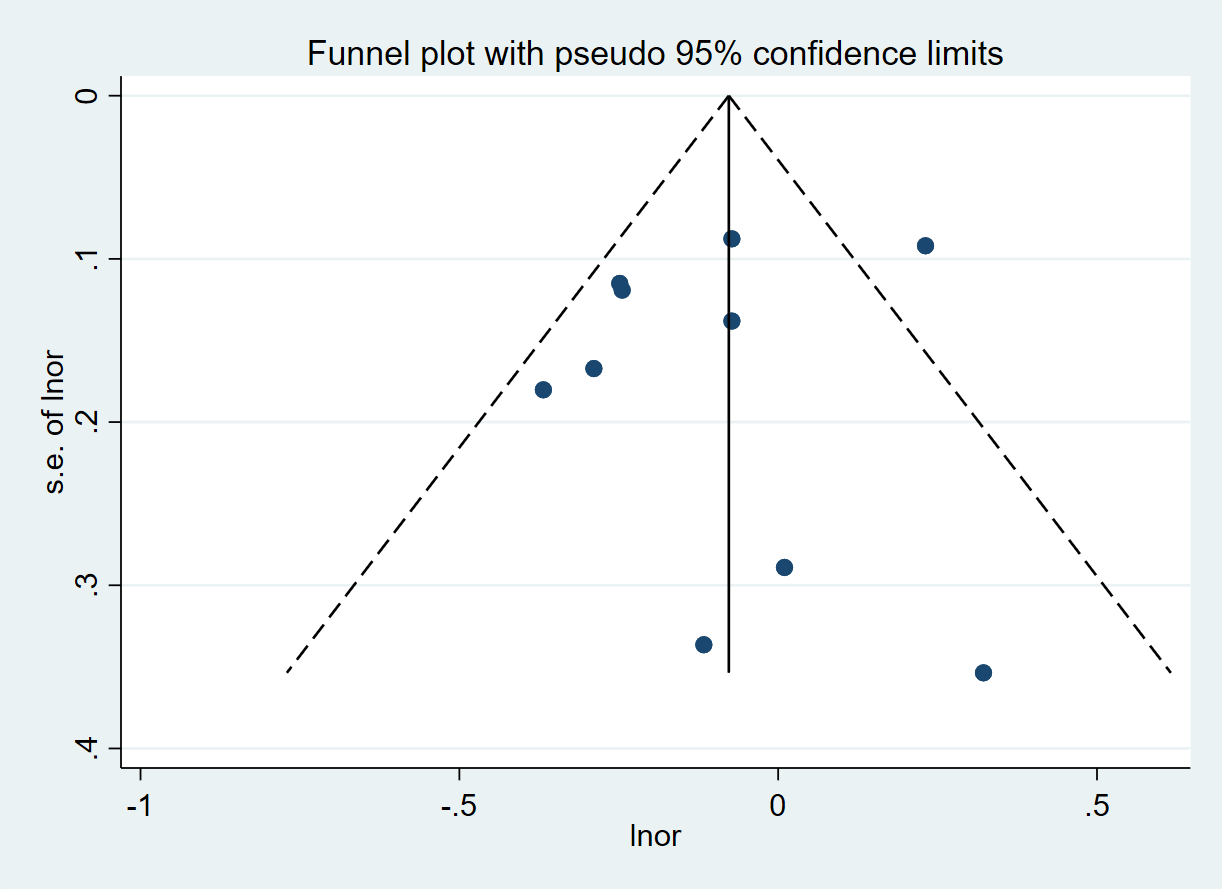


Figure S21: Funnel plot for the association between Carbohydrates intake and the incidence of hearing loss.
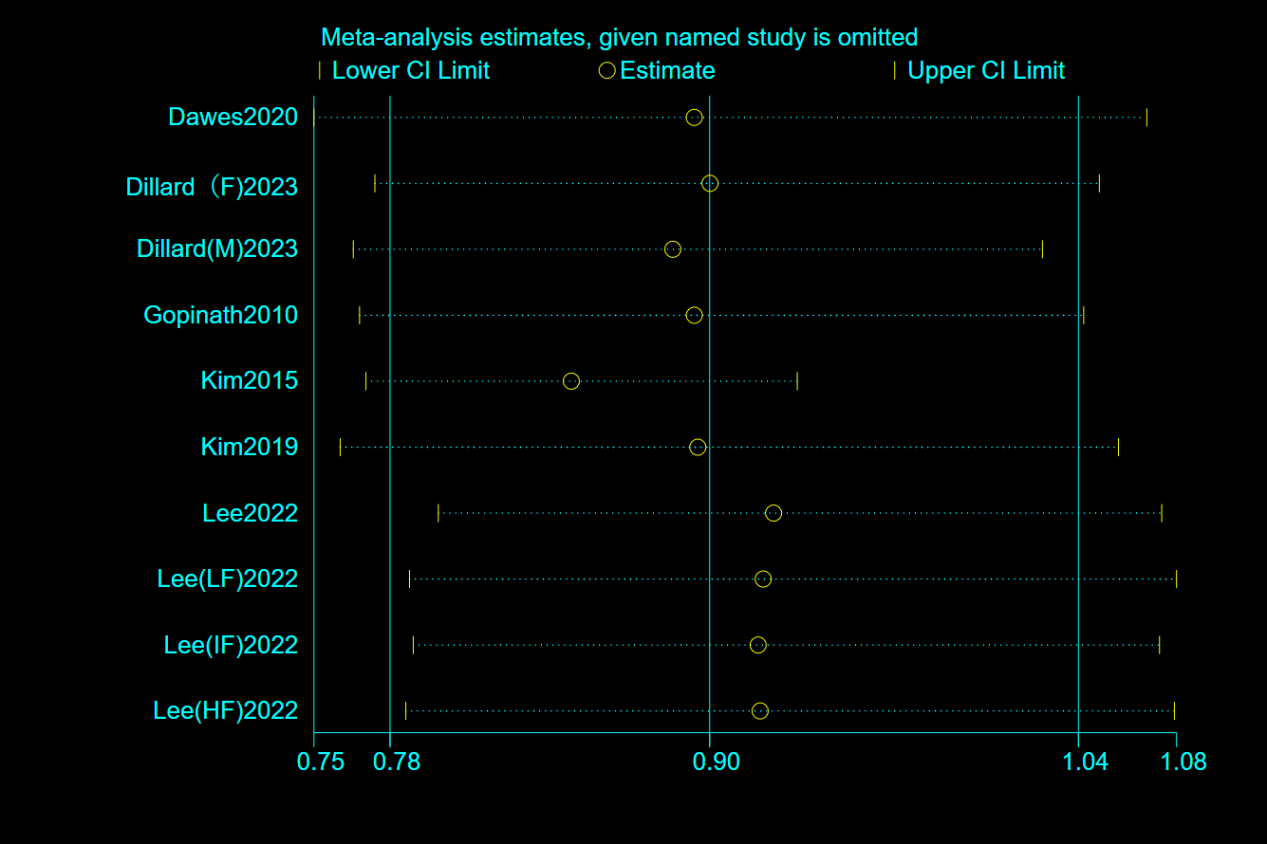


Figure S22: Sensitivity analysis for the effect of Carbohydrates intake on the incidence of hearing loss.


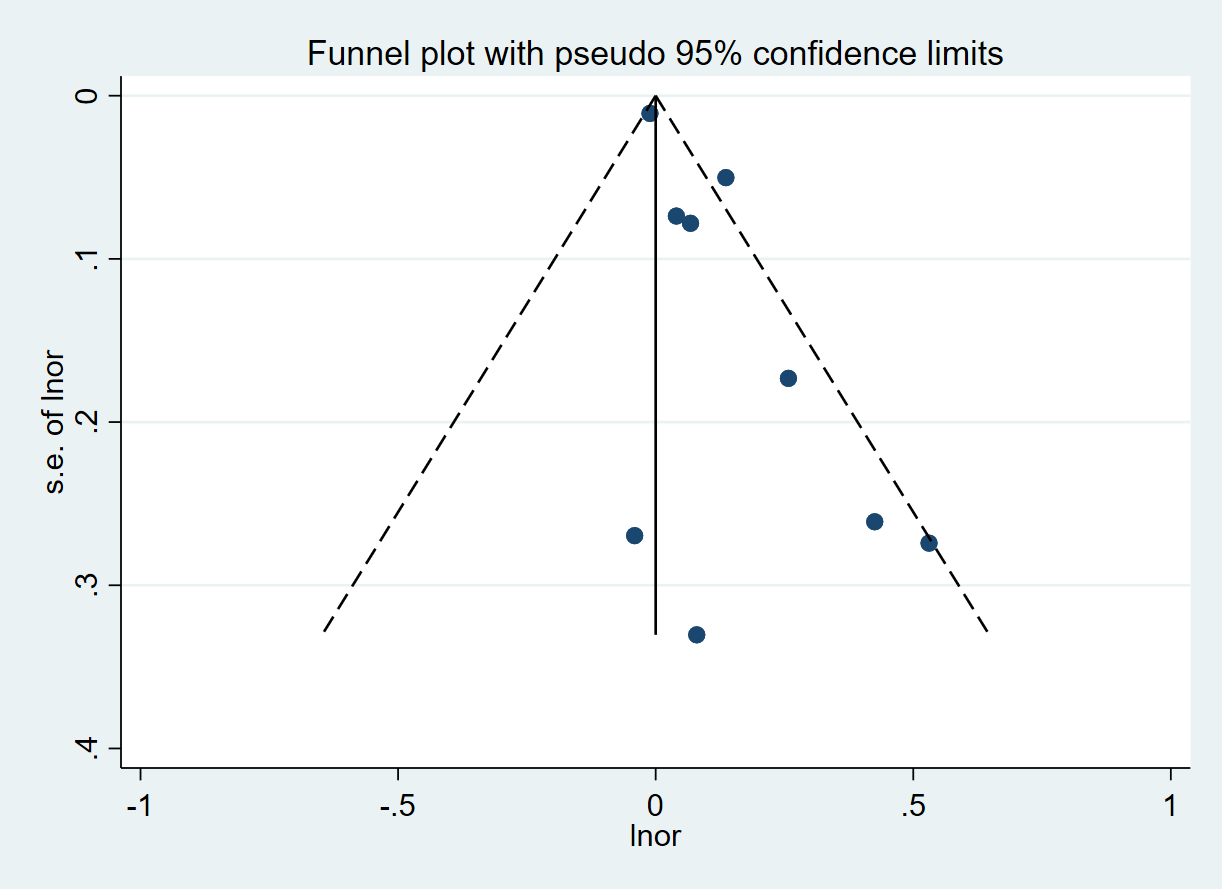


Figure S23: Funnel plot for the association between Sugar intake and the incidence of hearing loss.
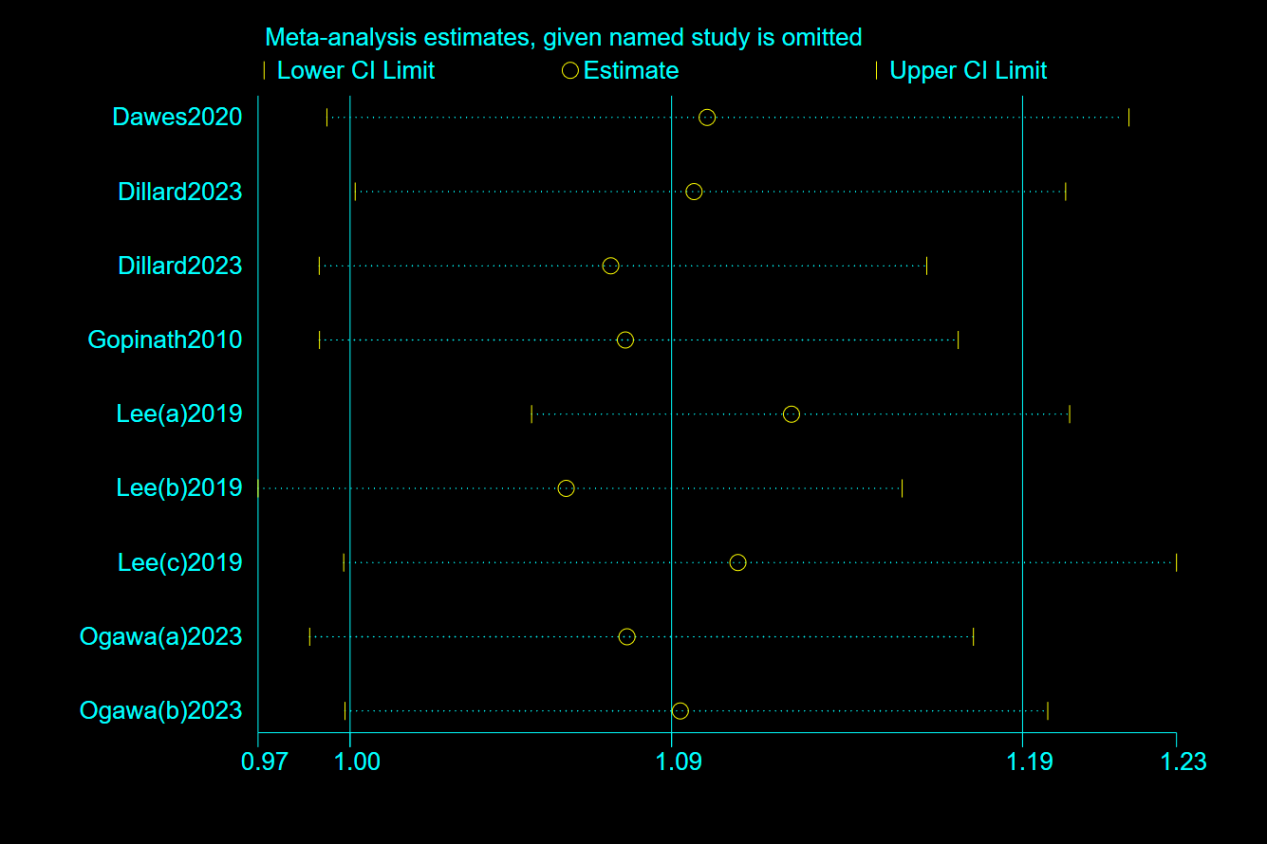


Figure S24: Sensitivity analysis for the effect of Sugar intake on the incidence of hearing loss.


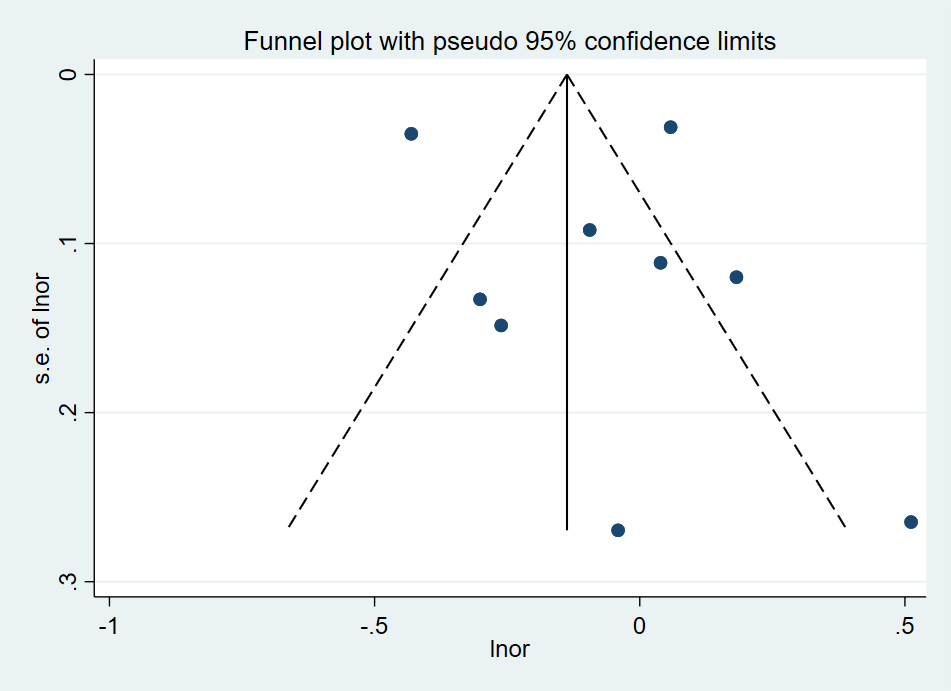


Figure S25: Funnel plot for the association between Alcohol intake and the incidence of hearing loss.
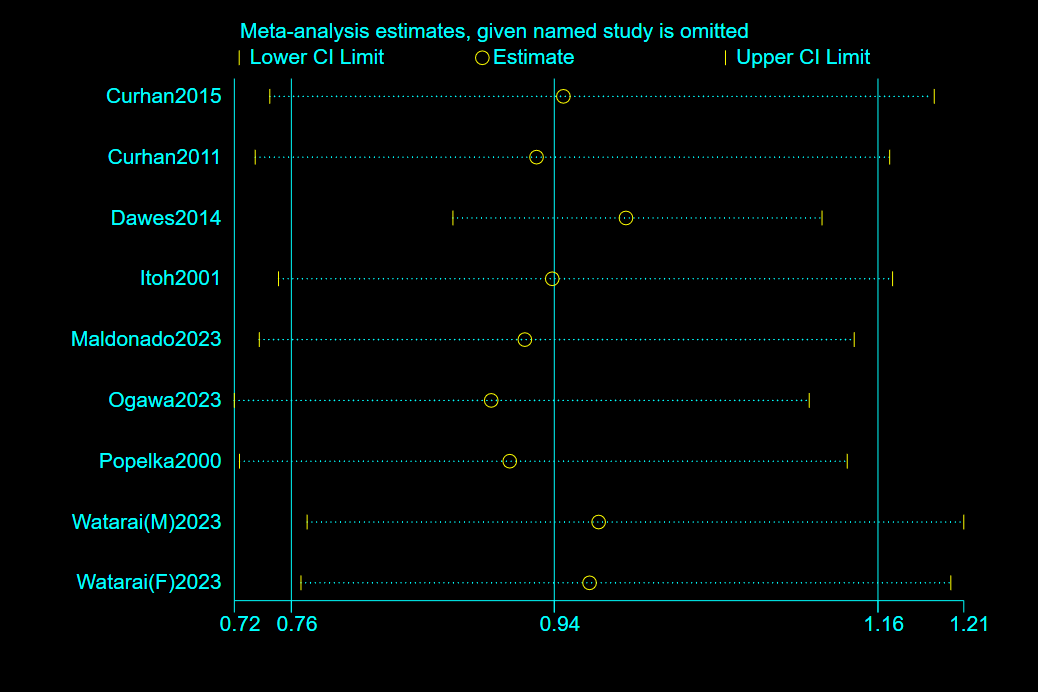


Figure S26: Sensitivity analysis for the effect of Alcohol intake on the incidence of hearing loss.


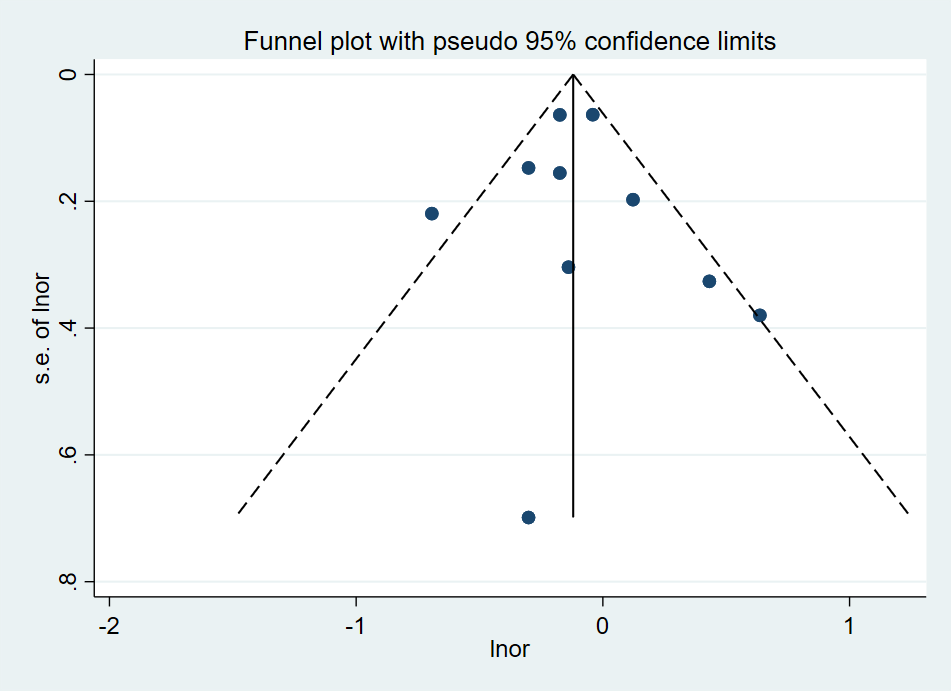


Figure S27: Funnel plot for the association between Coffee intake and the incidence of hearing loss.
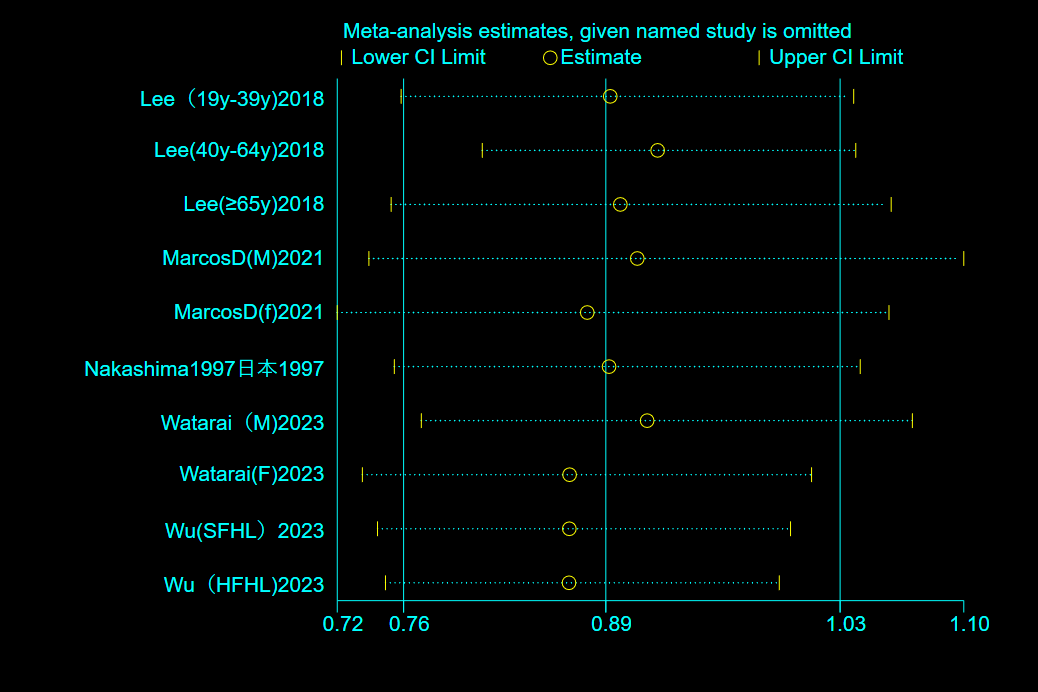


Figure S28: Sensitivity analysis for the effect of Coffee intake on the incidence of hearing loss.
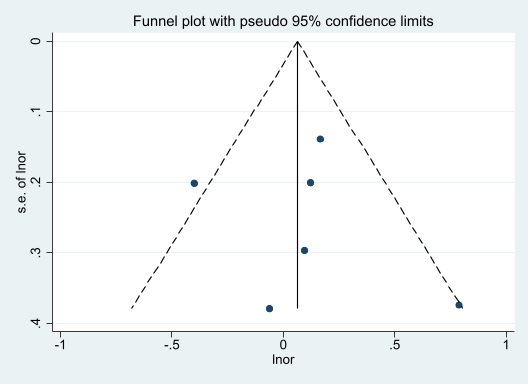


Figure S29: Funnel plot for the association between Tea intake and the incidence of hearing loss.
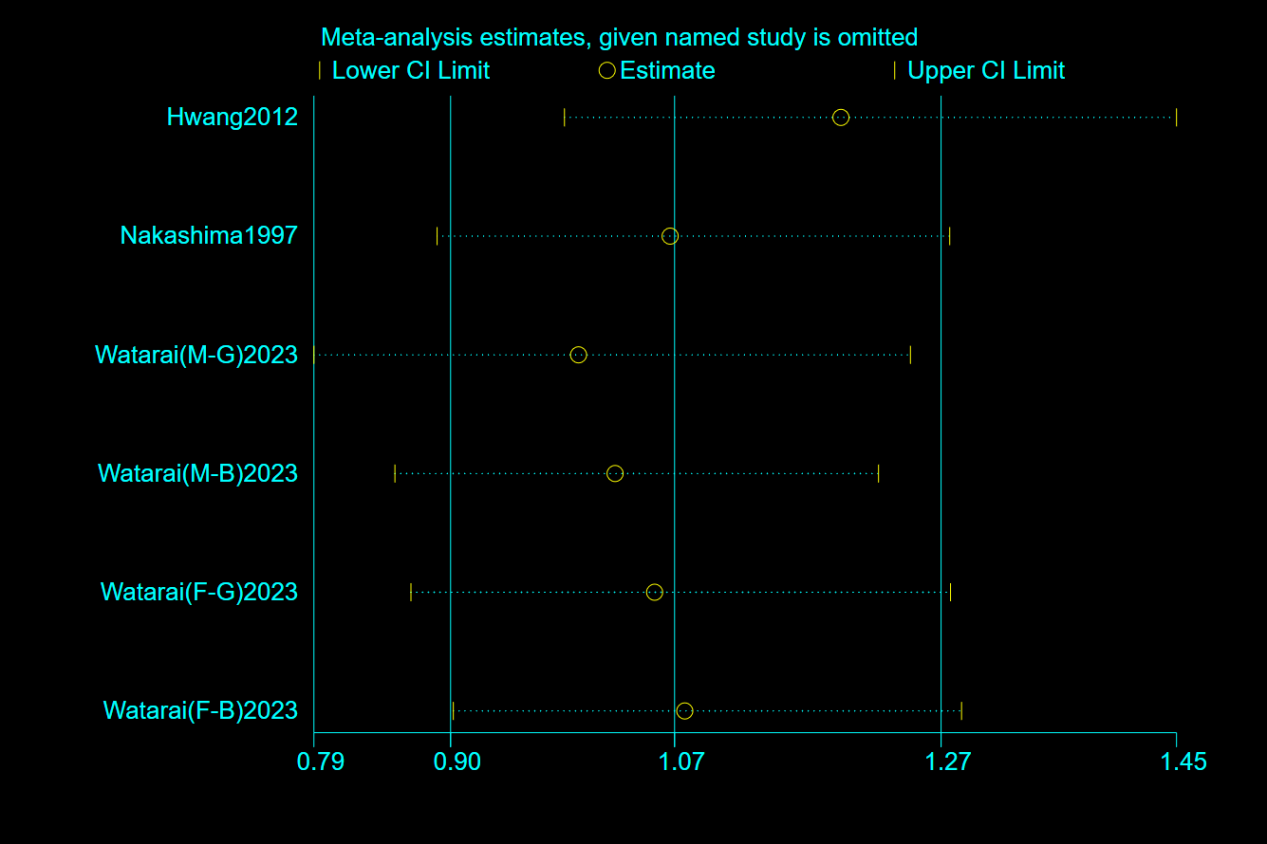


Figure S30: Sensitivity analysis for the effect of Tea intake on the incidence of hearing loss.


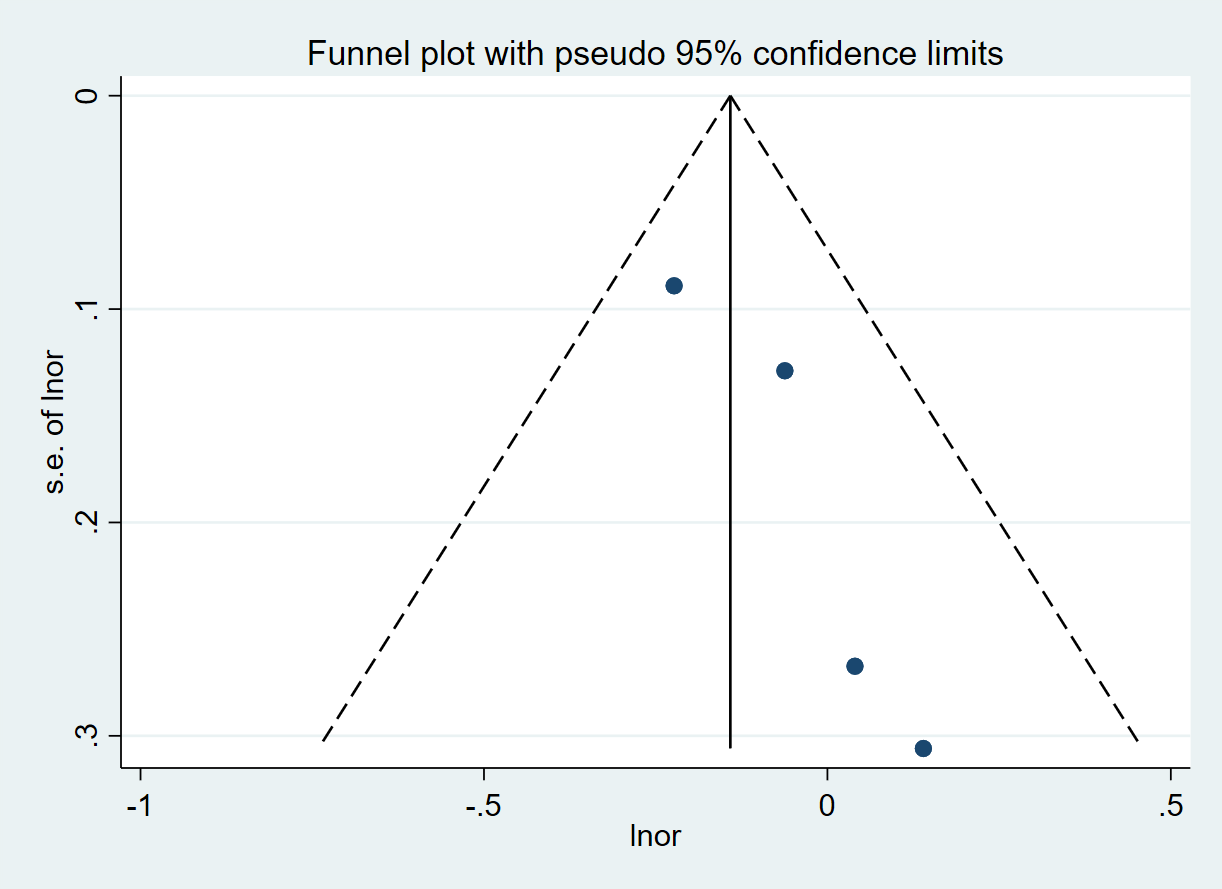


Figure S31: Funnel plot for the association between Fish intake and the incidence of hearing loss.


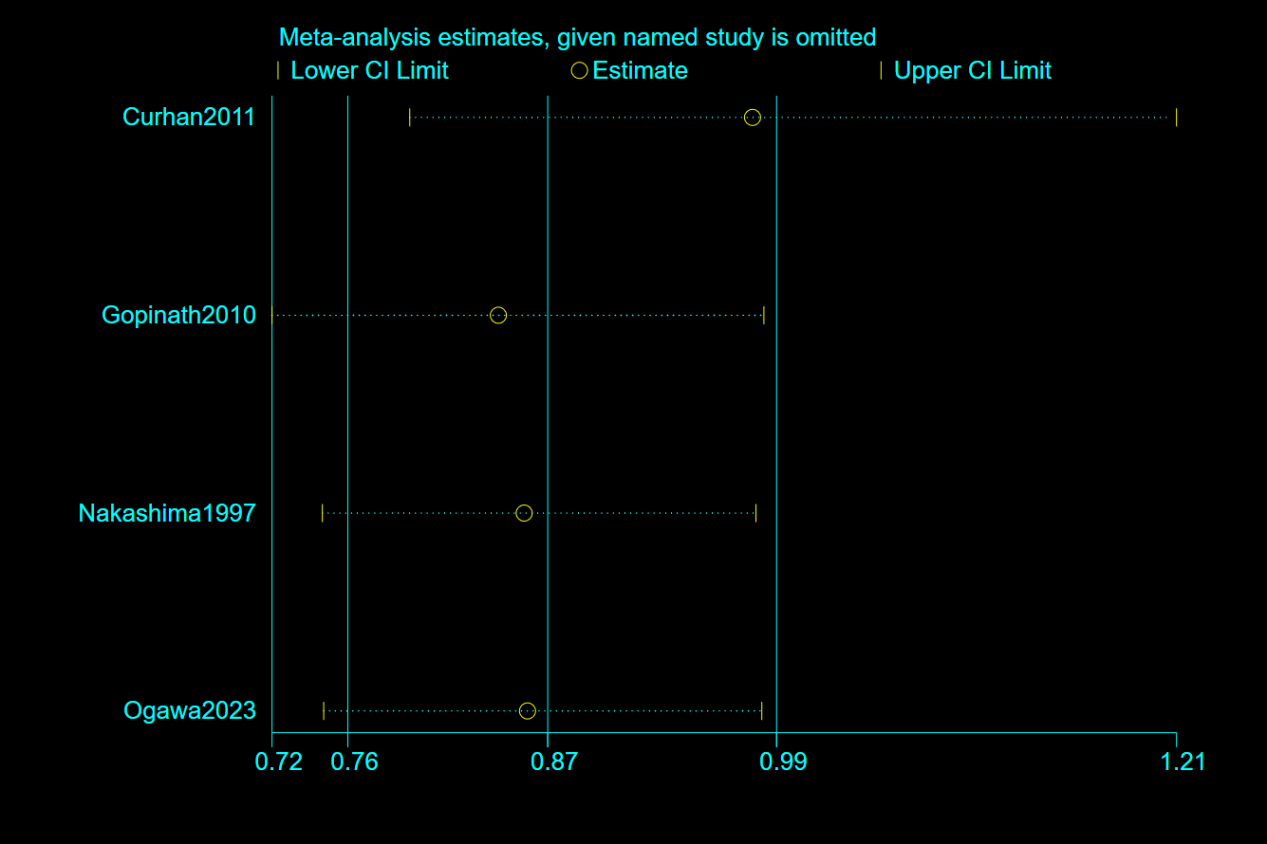


Figure S32: Sensitivity analysis for the effect of Fish intake on the incidence of hearing loss.

Figure 3: Forest maps for Vitamin A intake and incidence of hearing loss.

Figure 4: Forest maps for Vitamin B intake and incidence of hearing loss.

Figure 5: Forest maps for Vitamin C intake and incidence of hearing loss.

Figure 6: Forest maps for Vitamin E intake and incidence of hearing loss.

Figure 7: Forest maps for Carotenoids intake and incidence of hearing loss.

Figure 8: Forest maps for Carotenoid Types intake and incidence of hearing loss.

Figure 9: Forest maps for Minerals intake and incidence of hearing loss.

Figure 10: Forest maps for Fat intake and incidence of hearing loss.

Figure 11: Forest maps for Protein intake and incidence of hearing loss.

Figure 12: Forest maps for Fiber intake and incidence of hearing loss.

Figure 13: Forest maps for Carbohydrates intake and incidence of hearing loss.

Figure 14: Forest maps for Sugar intake and incidence of hearing loss.

Figure 15: Forest maps for Alcohol intake and incidence of hearing loss.

Figure 16: Forest maps for Coffee intake and incidence of hearing loss.

Figure 17: Forest maps for Tea intake and incidence of hearing loss.

Figure 18: Forest maps for Fish intake and incidence of hearing loss.
