# Supplementary material for: Protective effects of dietary nutrients on hearing loss: a systematic review and meta-analysis
Source: Front Nutr. 2025 May 9;12:1528771. doi: 10.3389/fnut.2025.1528771 (PMC12100664; doi:10.3389/fnut.2025.1528771)

**Meta-analysis of dietary nutrition and ARHL forest map**

Vitamin A


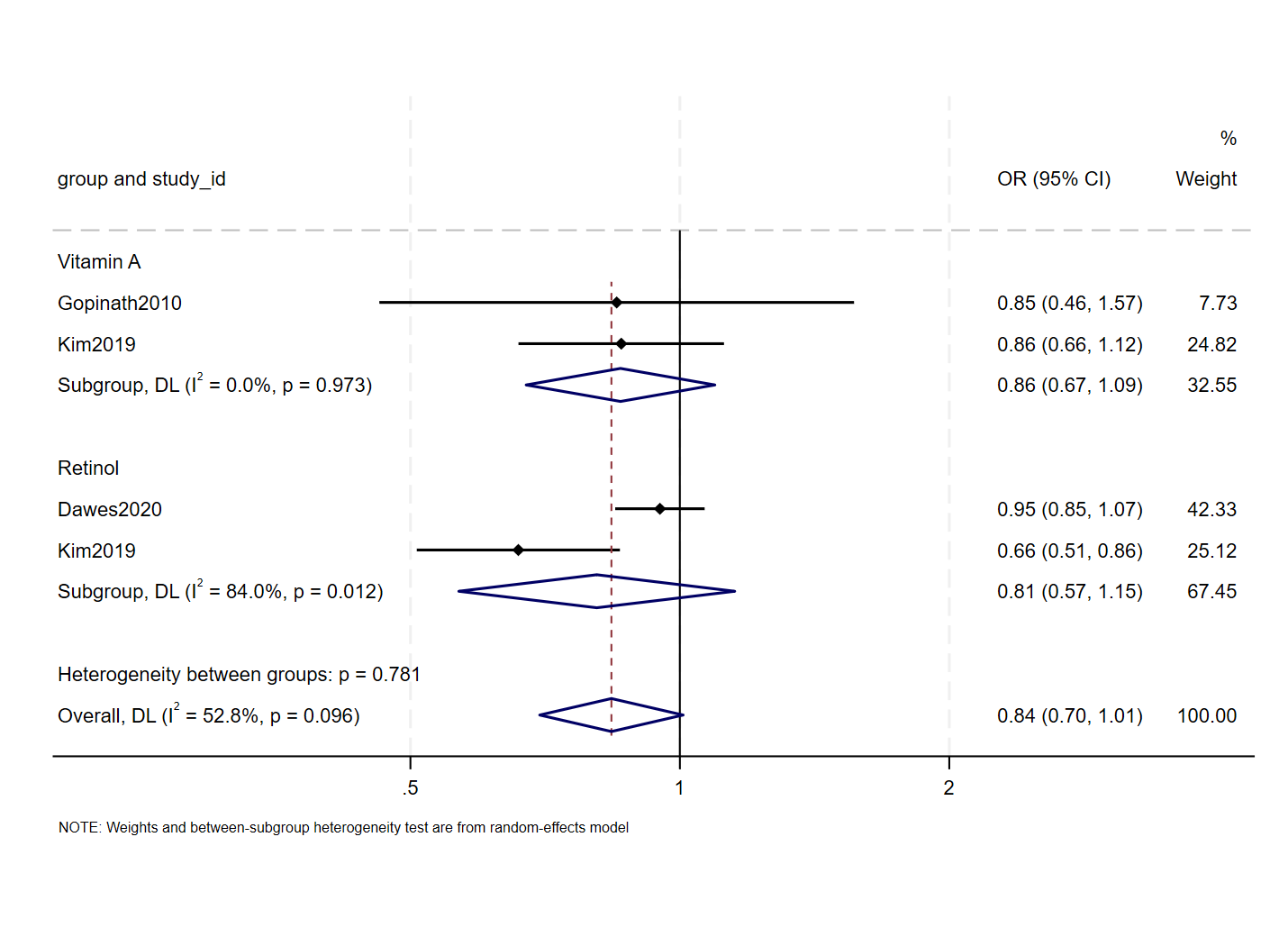


Vitamin B


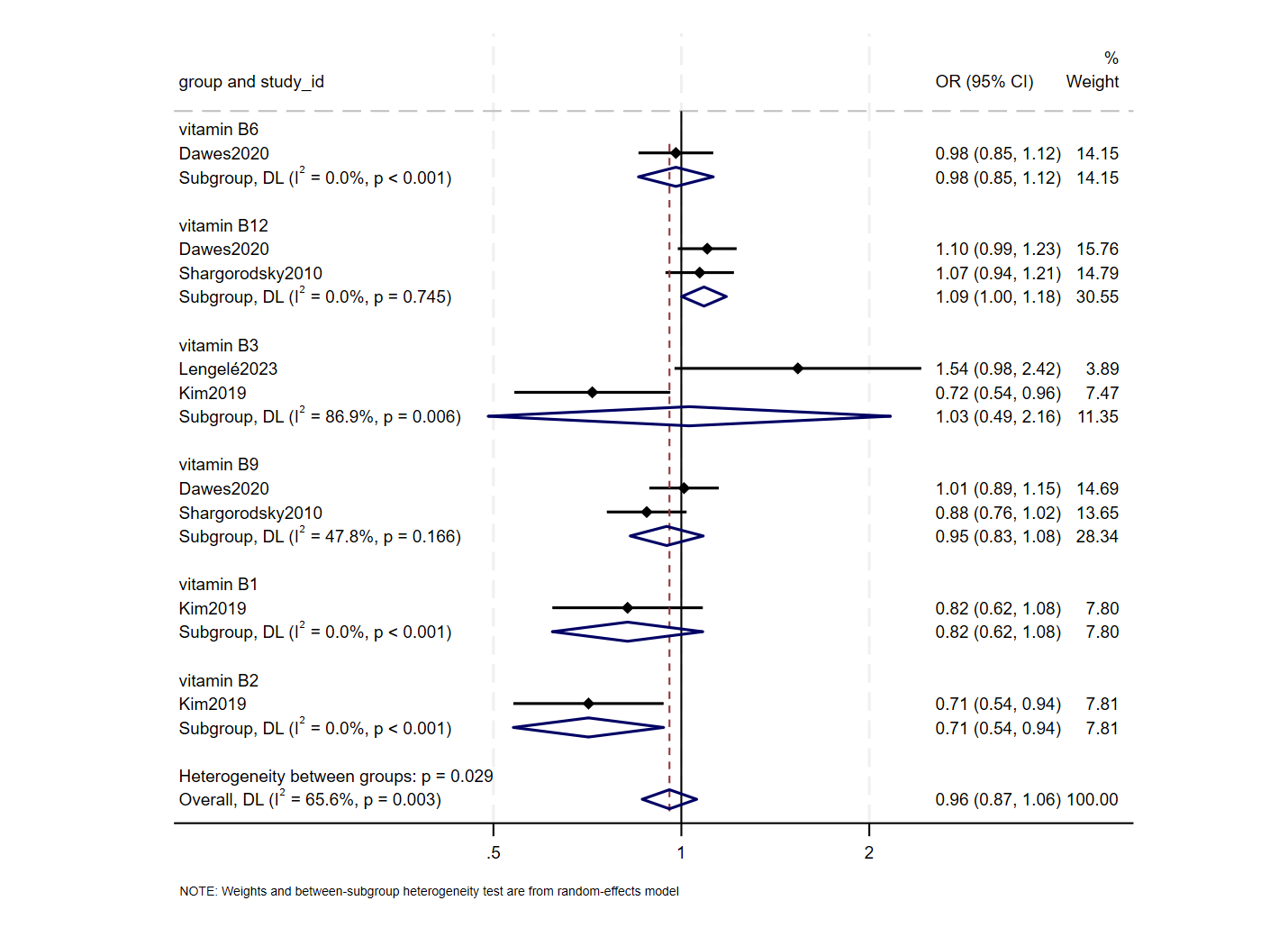


Vitamin C


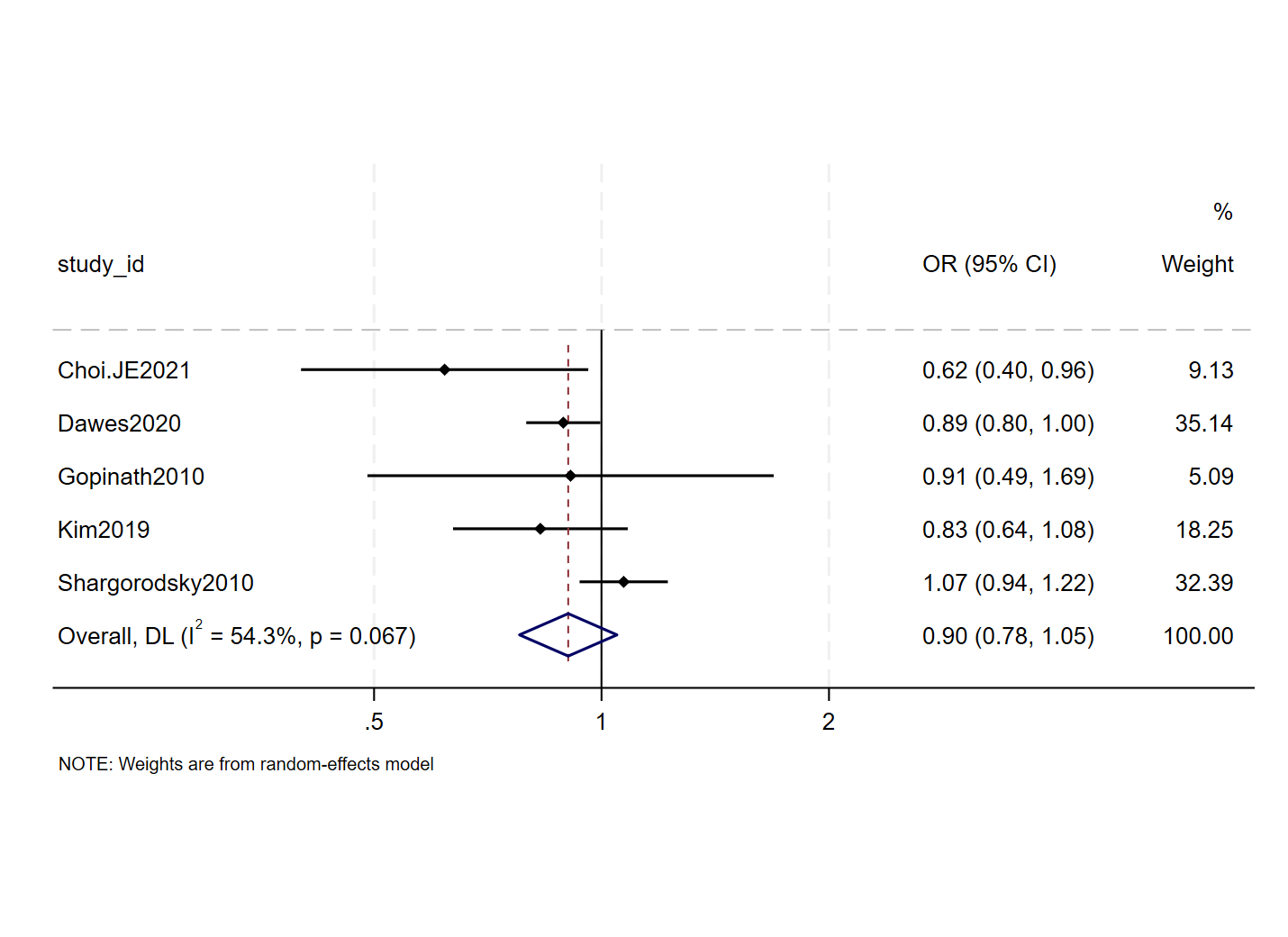


Vitamin E


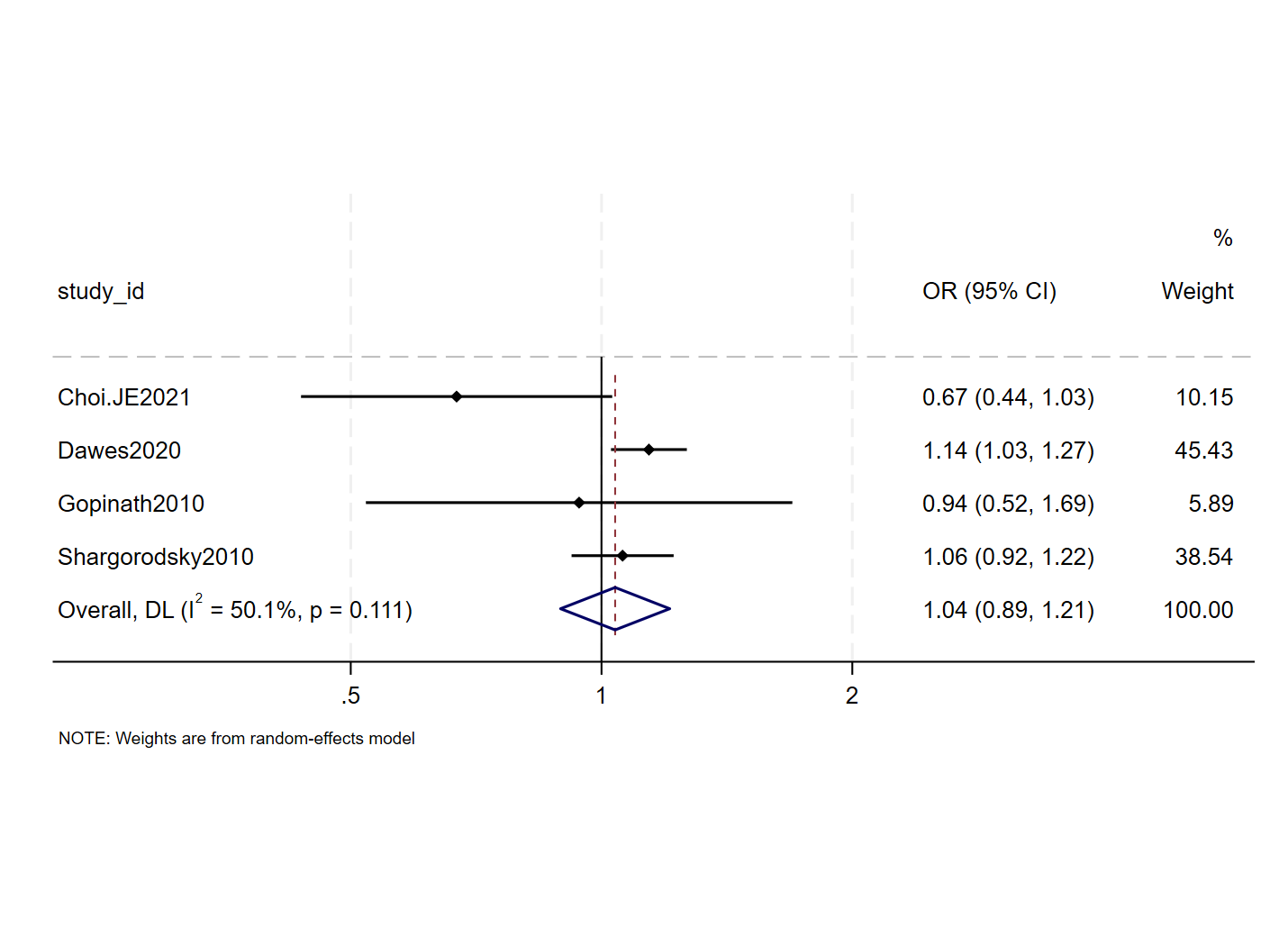


Carotenoids


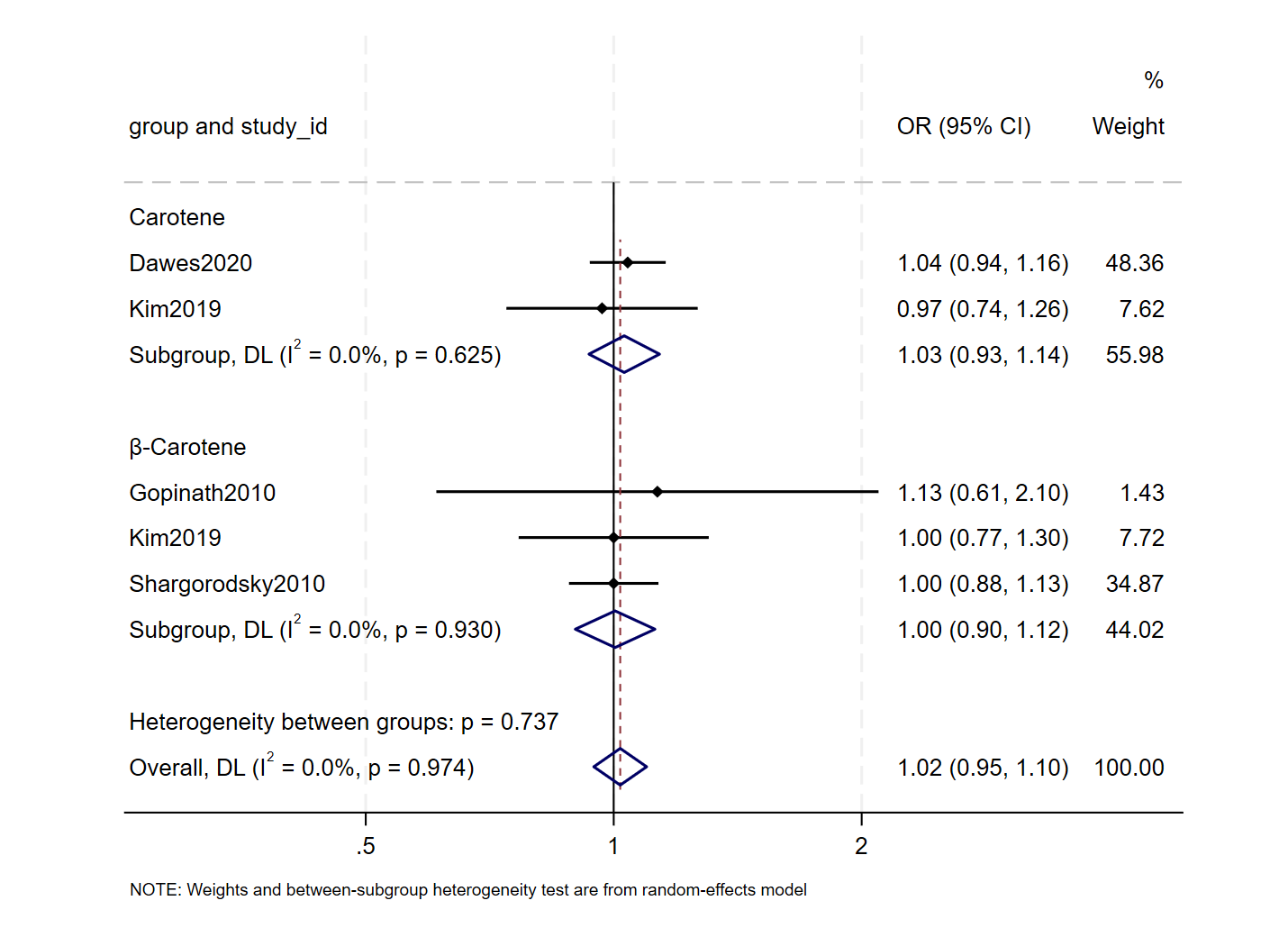


Carotenoid Types


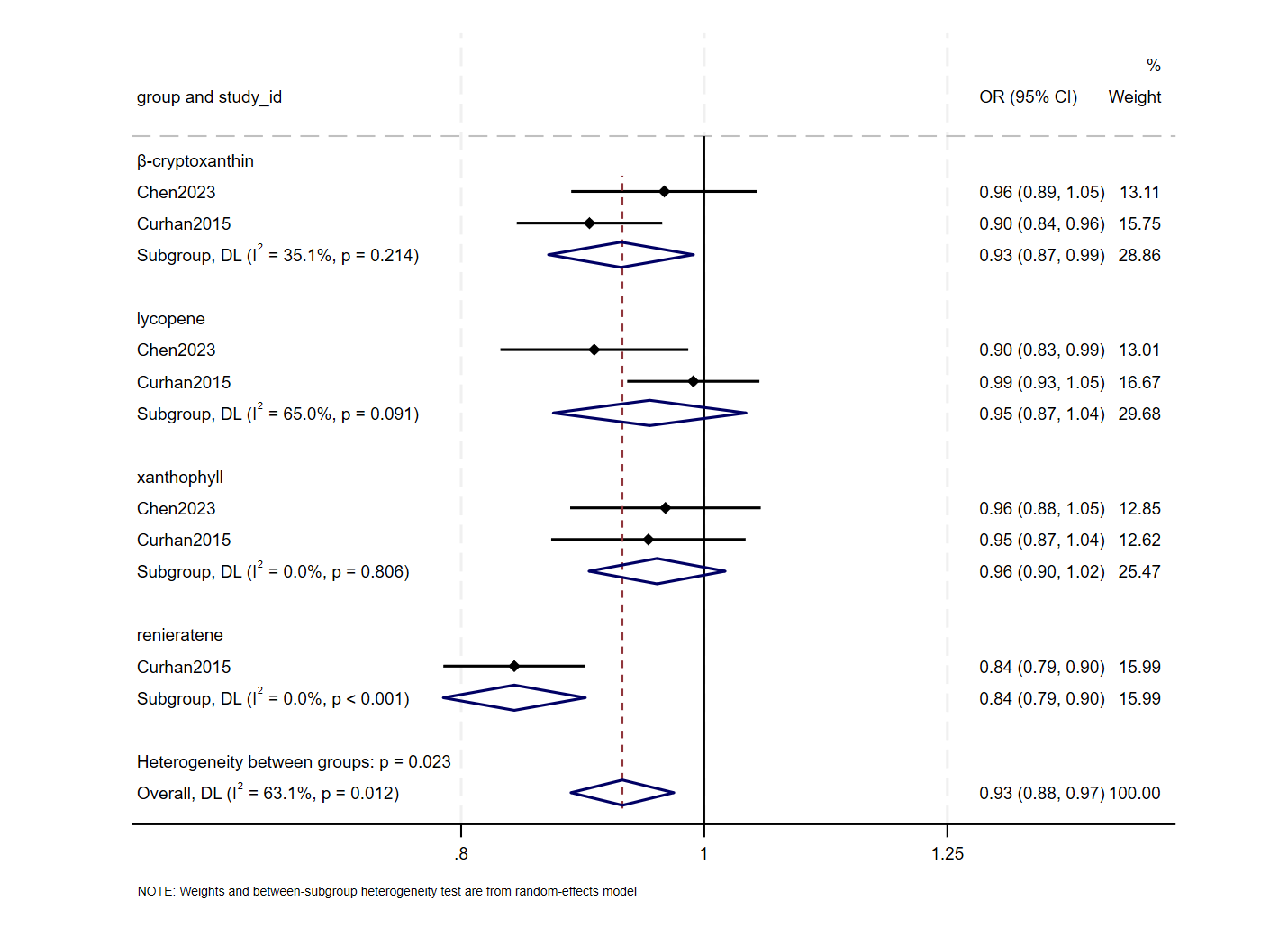


Minerals


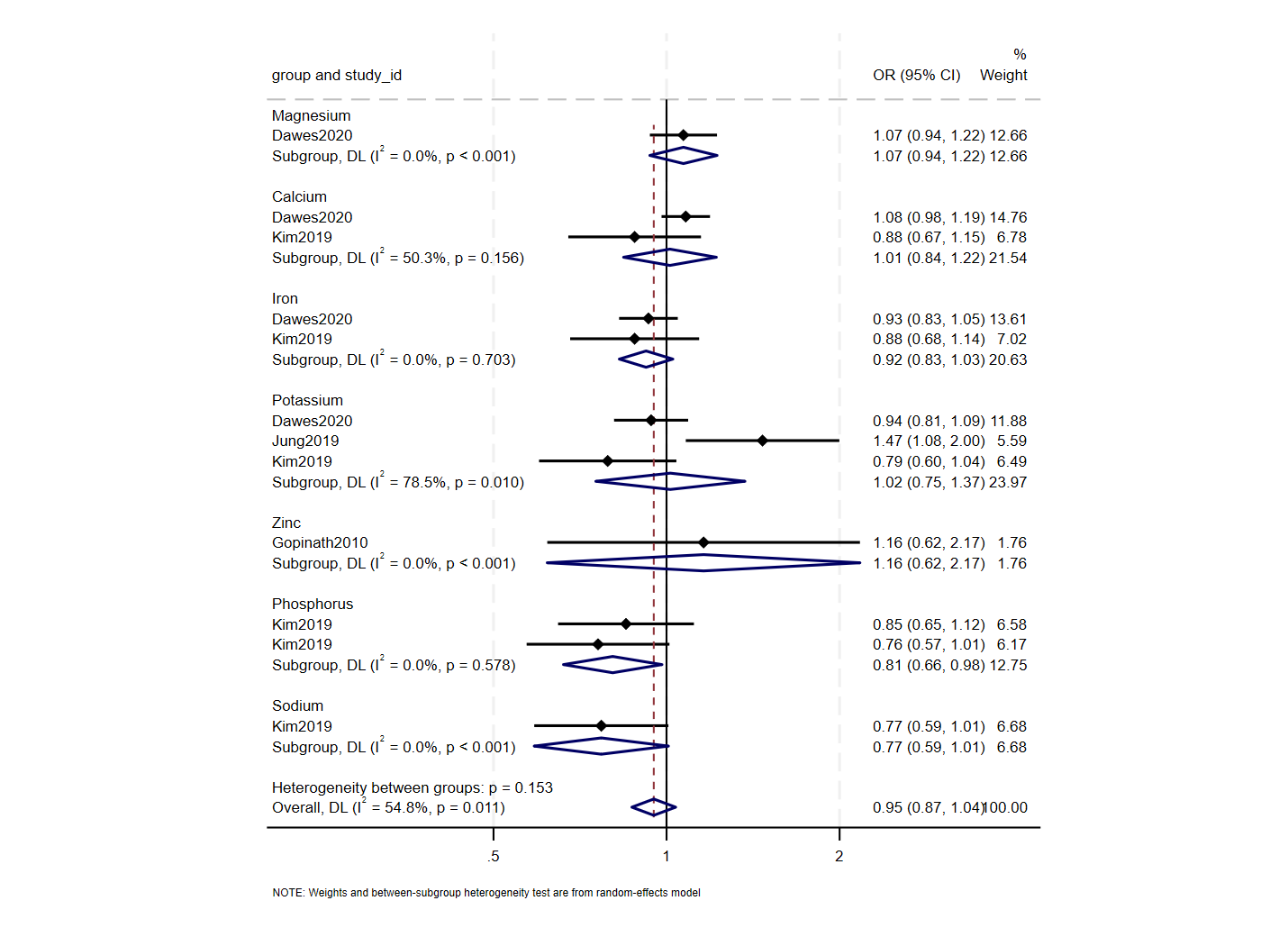


Fat


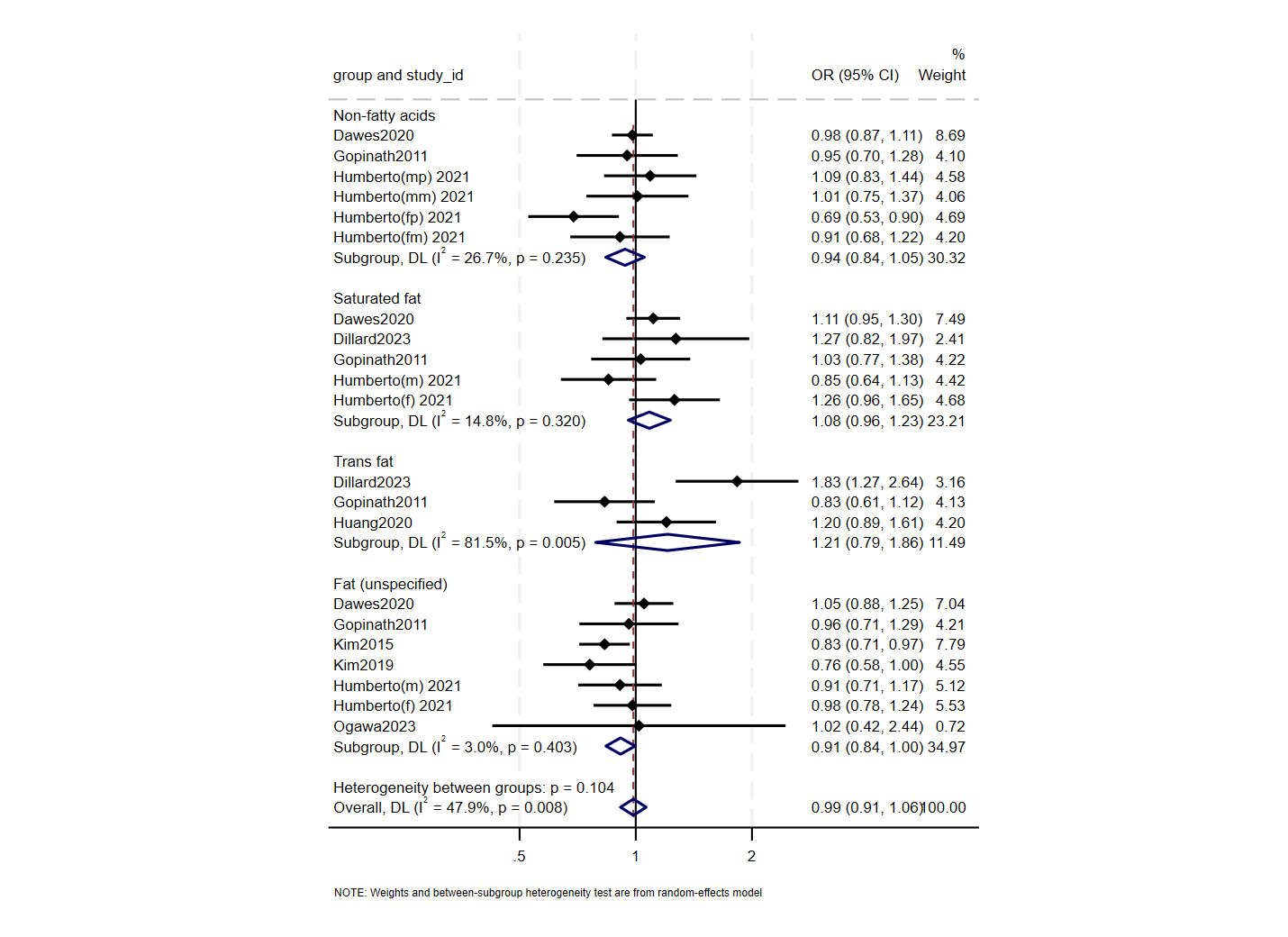


Protein


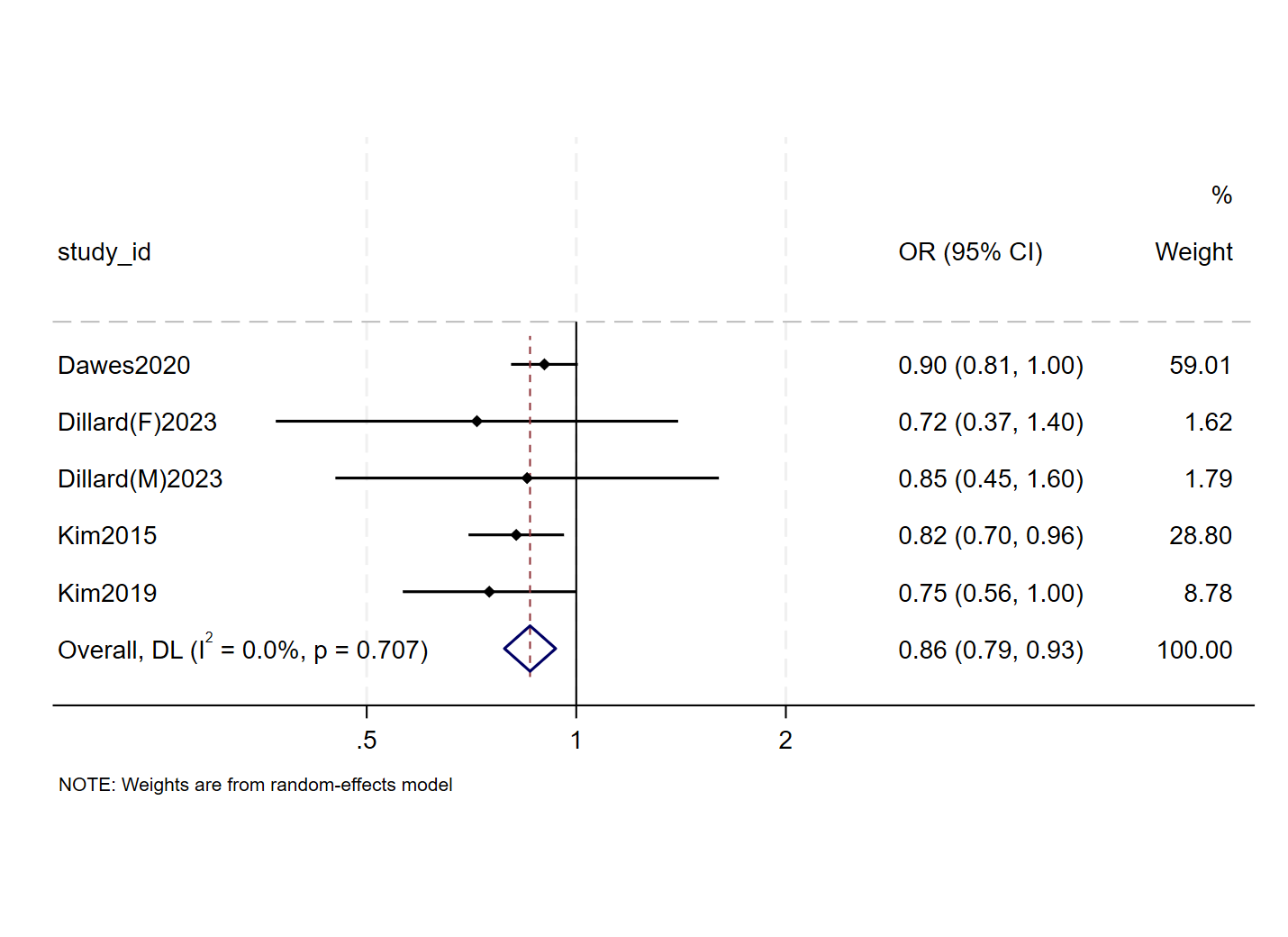


Fiber


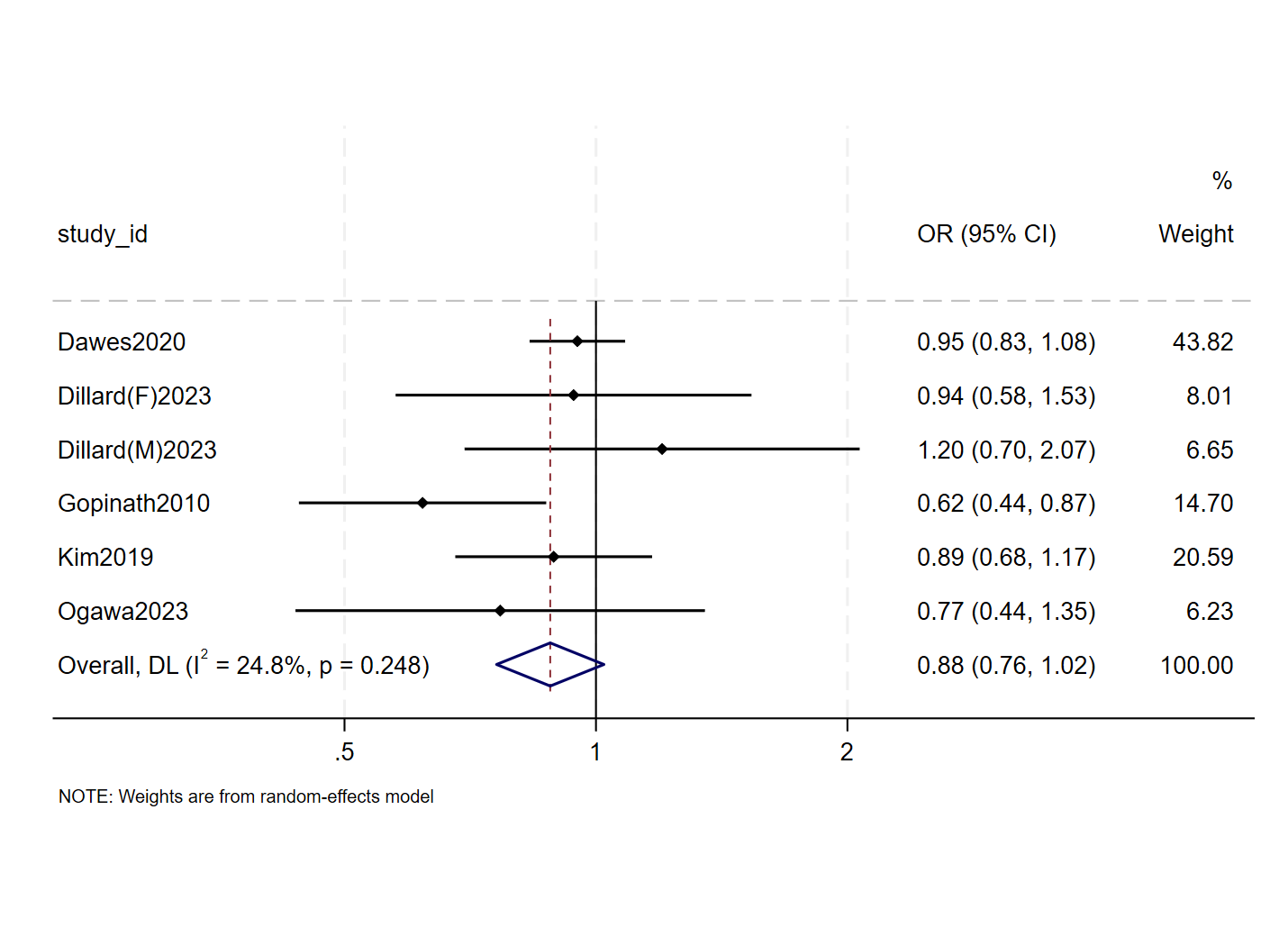


Sugar


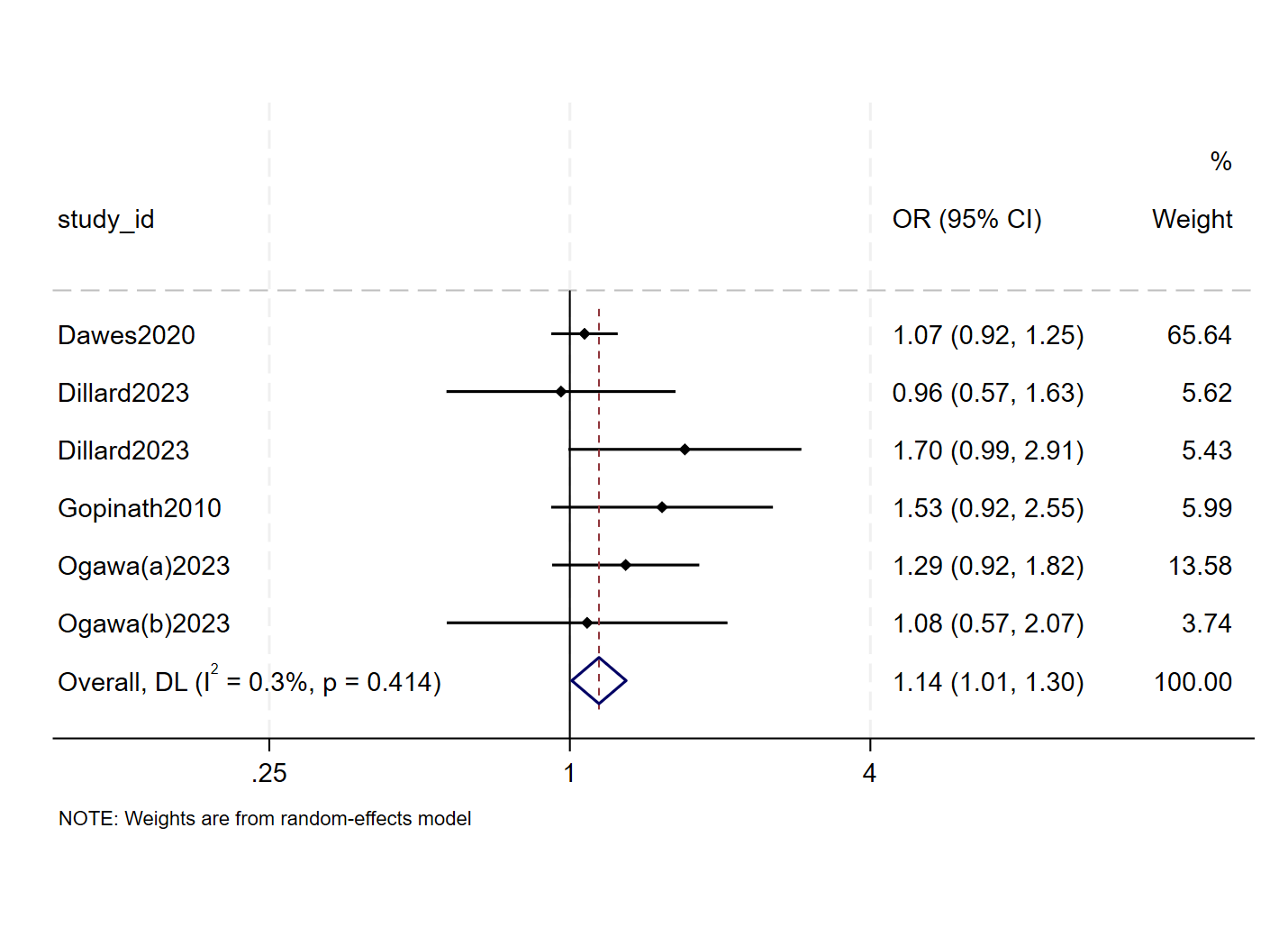


Alcohol


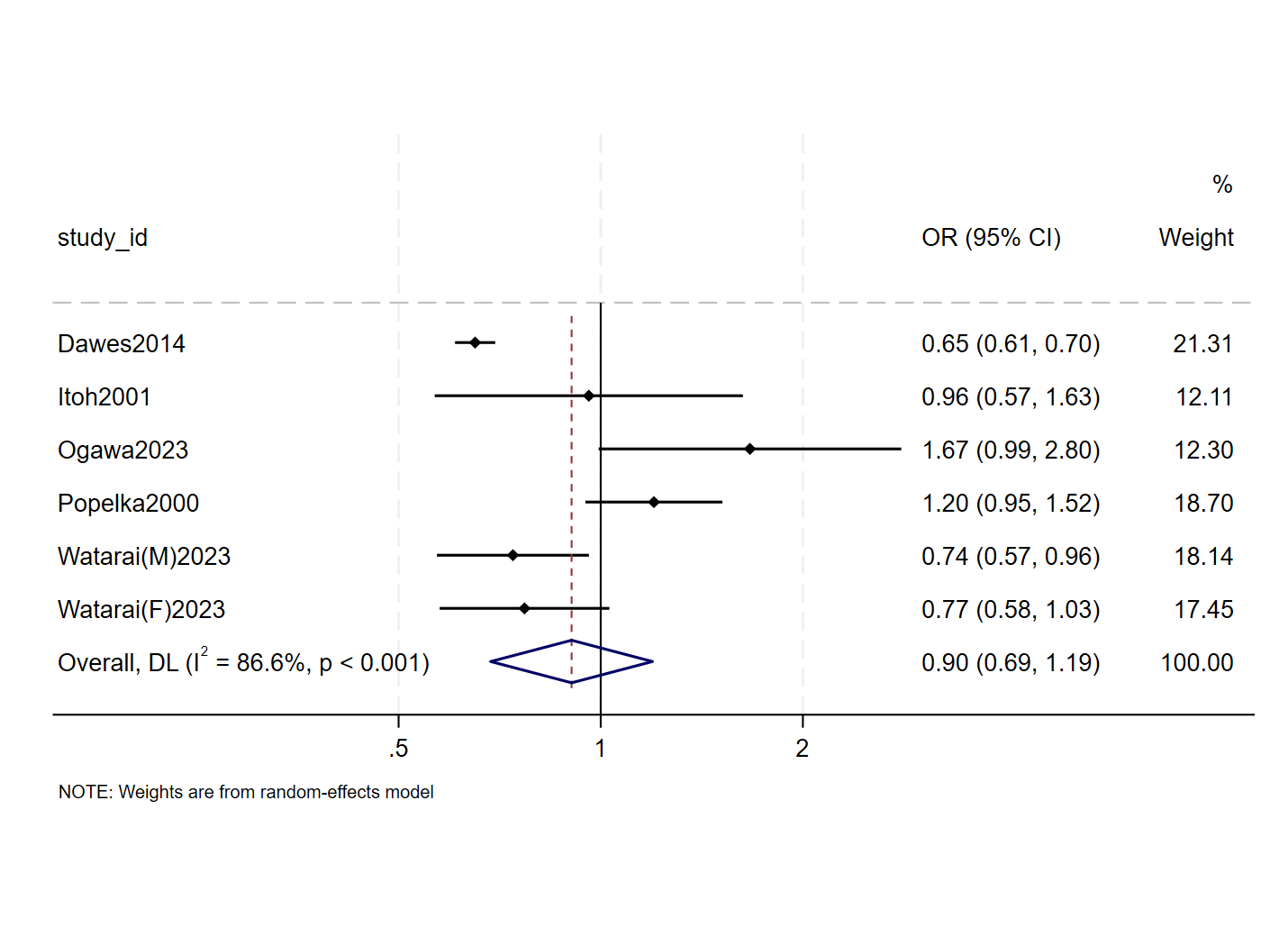


Coffee


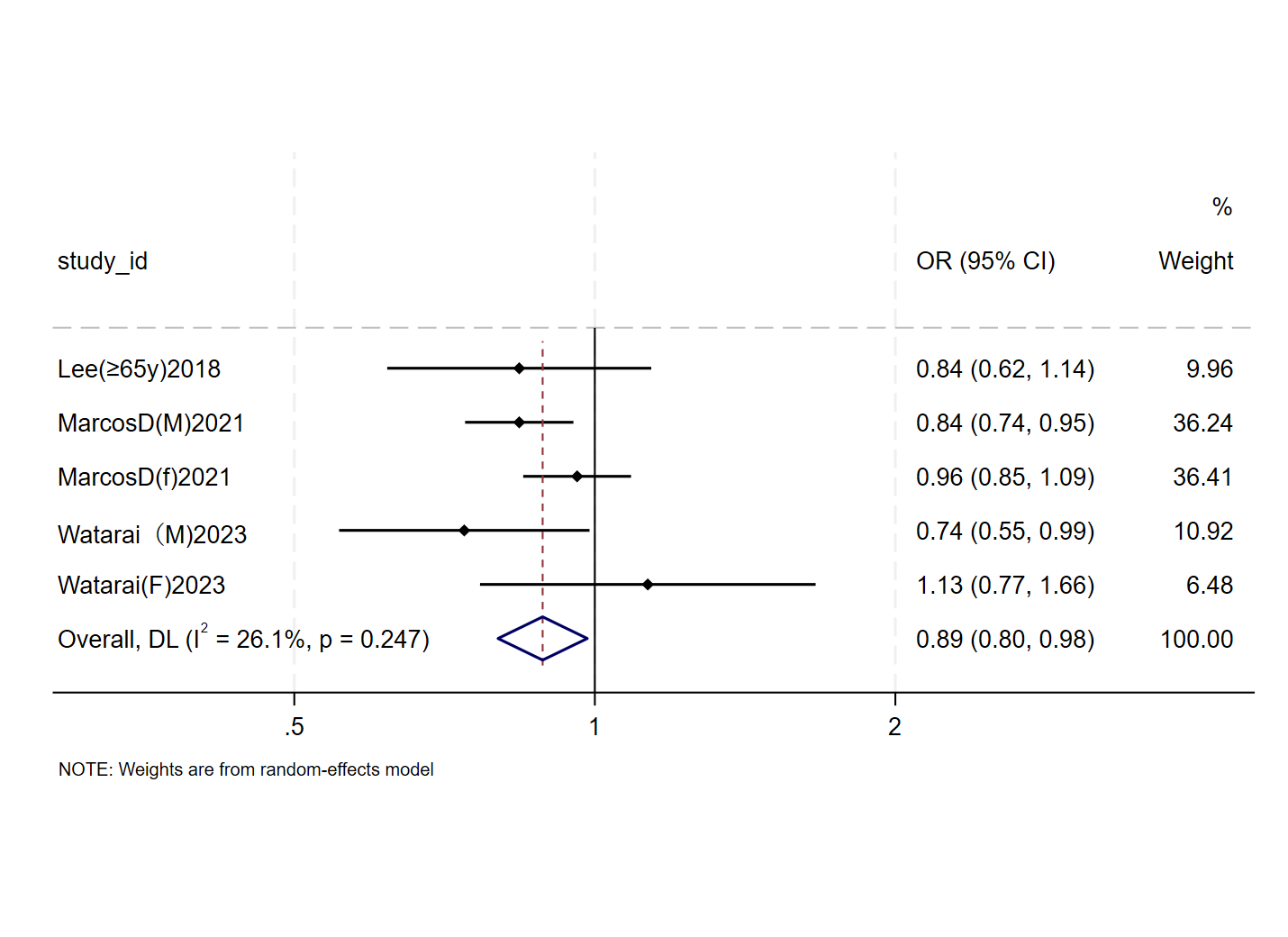


Tea
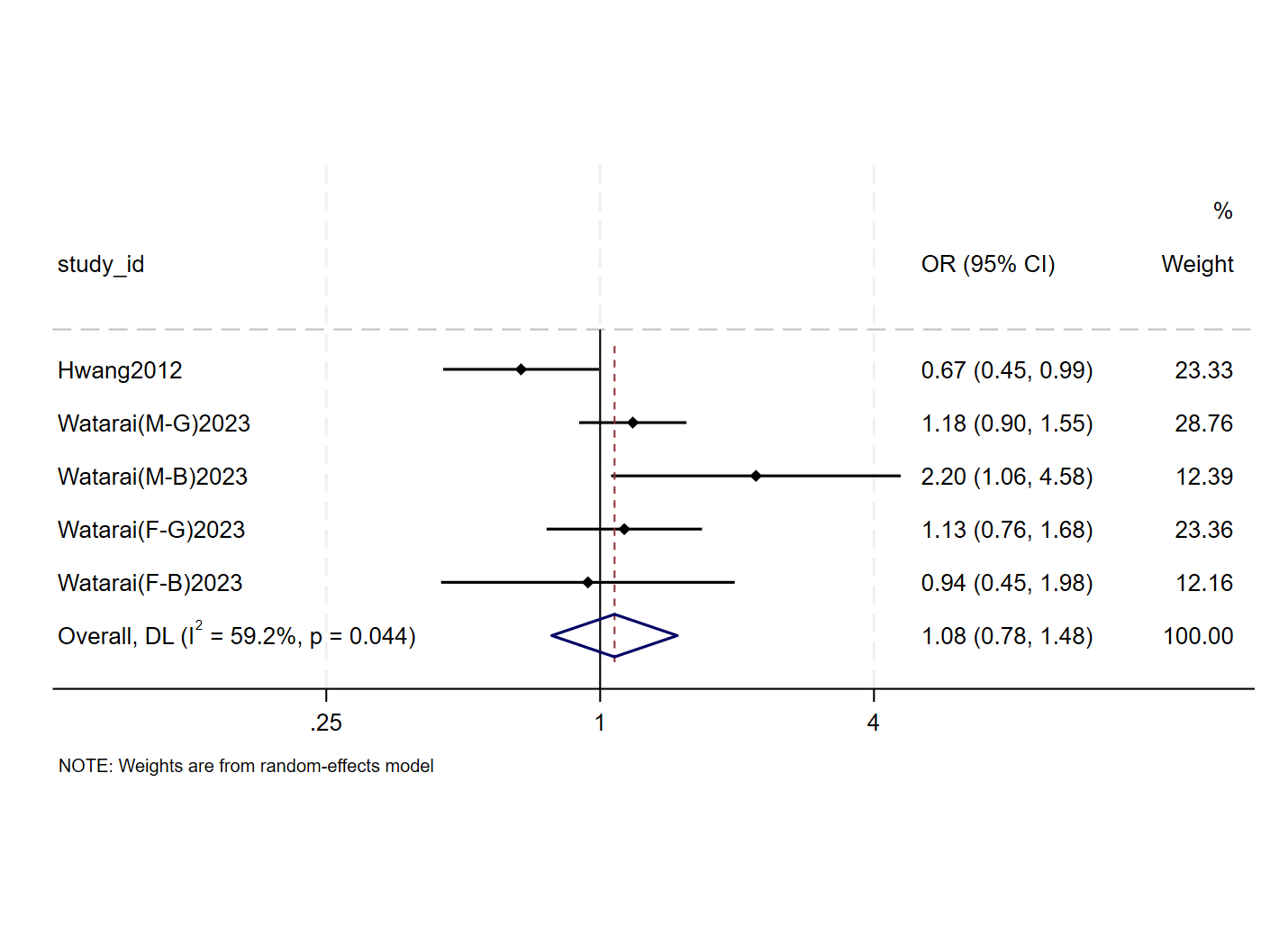


Fish


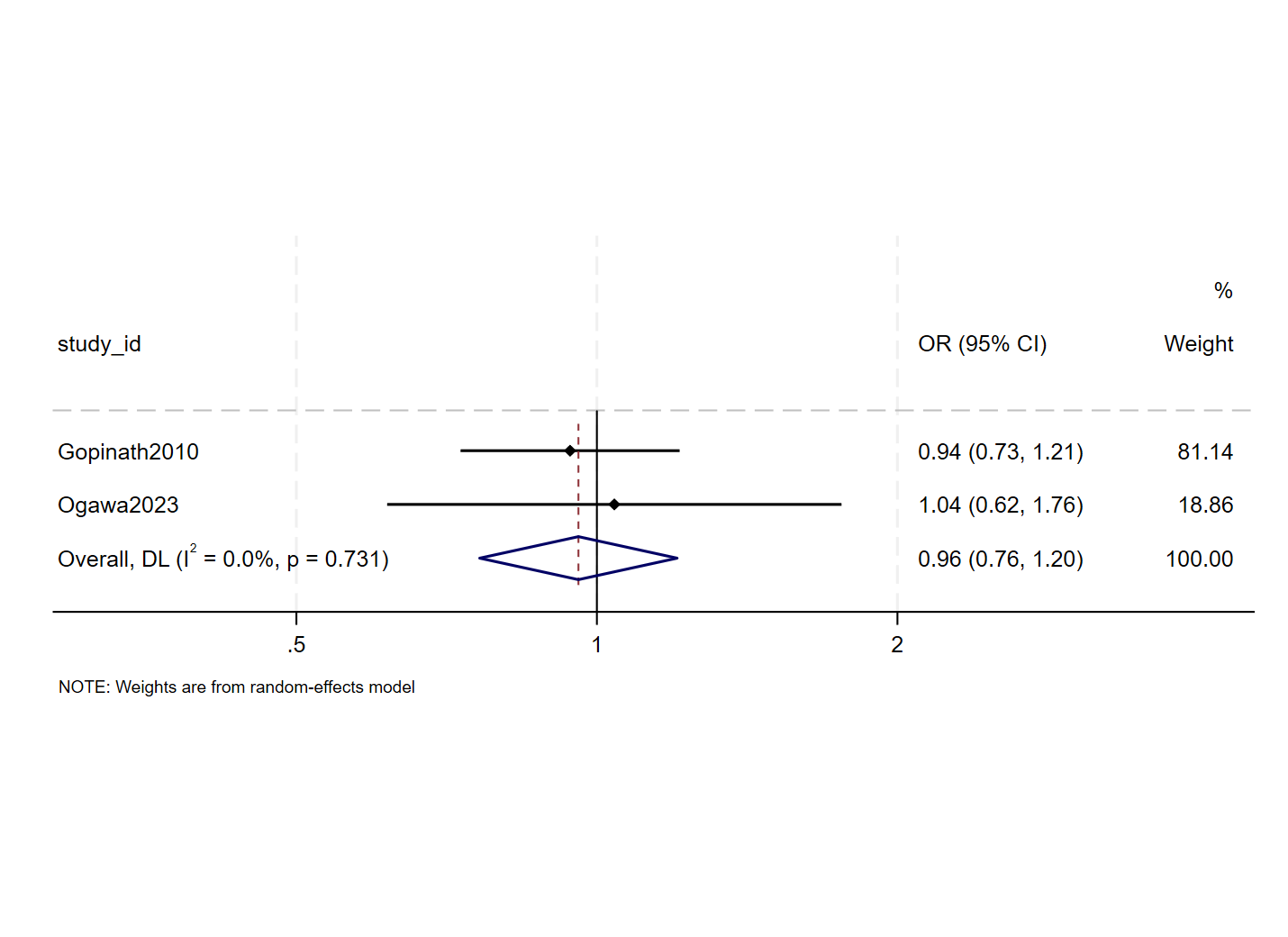

Supplement: Supplementary file 1 [file Data_Sheet_1.zip › 补充文件/Supplement Material 2 Meta-analysis of dietary nutrition and ARHL forest map.docx]
