# Supplementary material for: Protective effects of dietary nutrients on hearing loss: a systematic review and meta-analysis
Source: Front Nutr. 2025 May 9;12:1528771. doi: 10.3389/fnut.2025.1528771 (PMC12100664; doi:10.3389/fnut.2025.1528771)

Dietary Nutrition and Hearing loss (except ARHL)Meta-analysis of funnel plot

Vitamin A


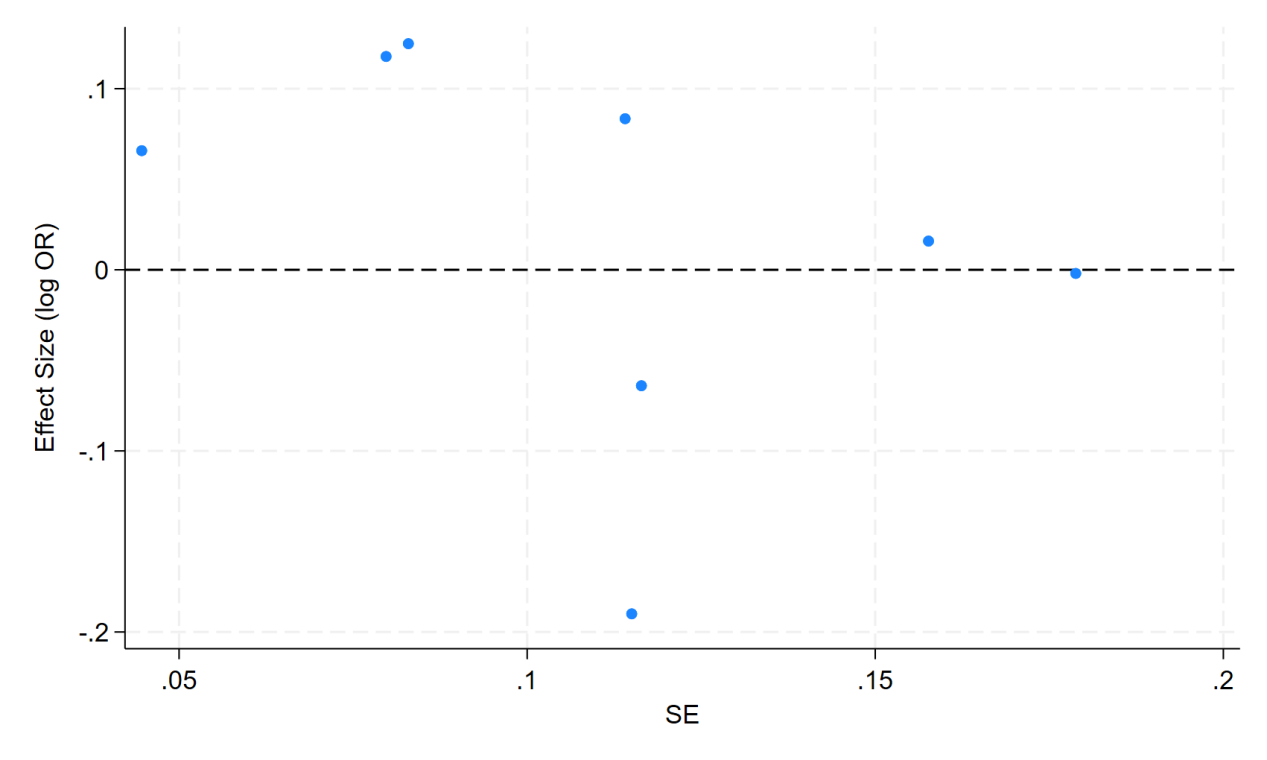


Vitamin B


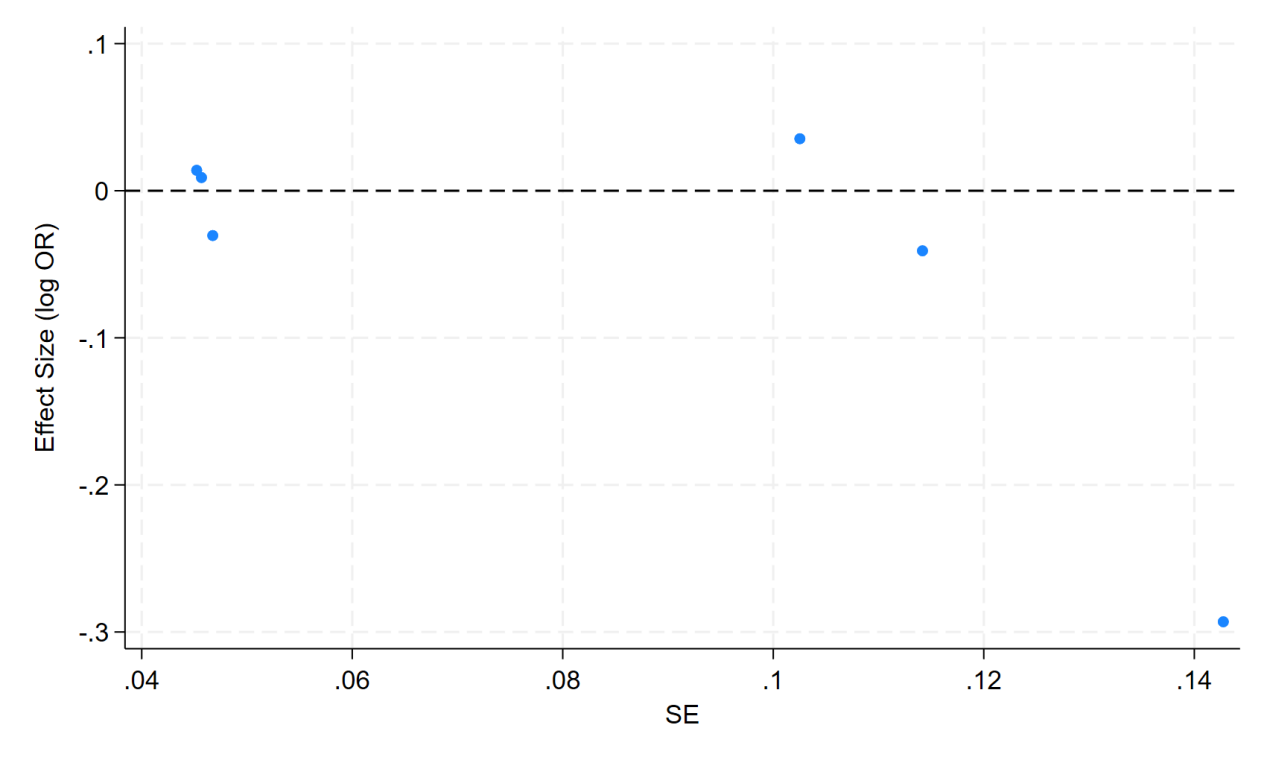


Vitamin C


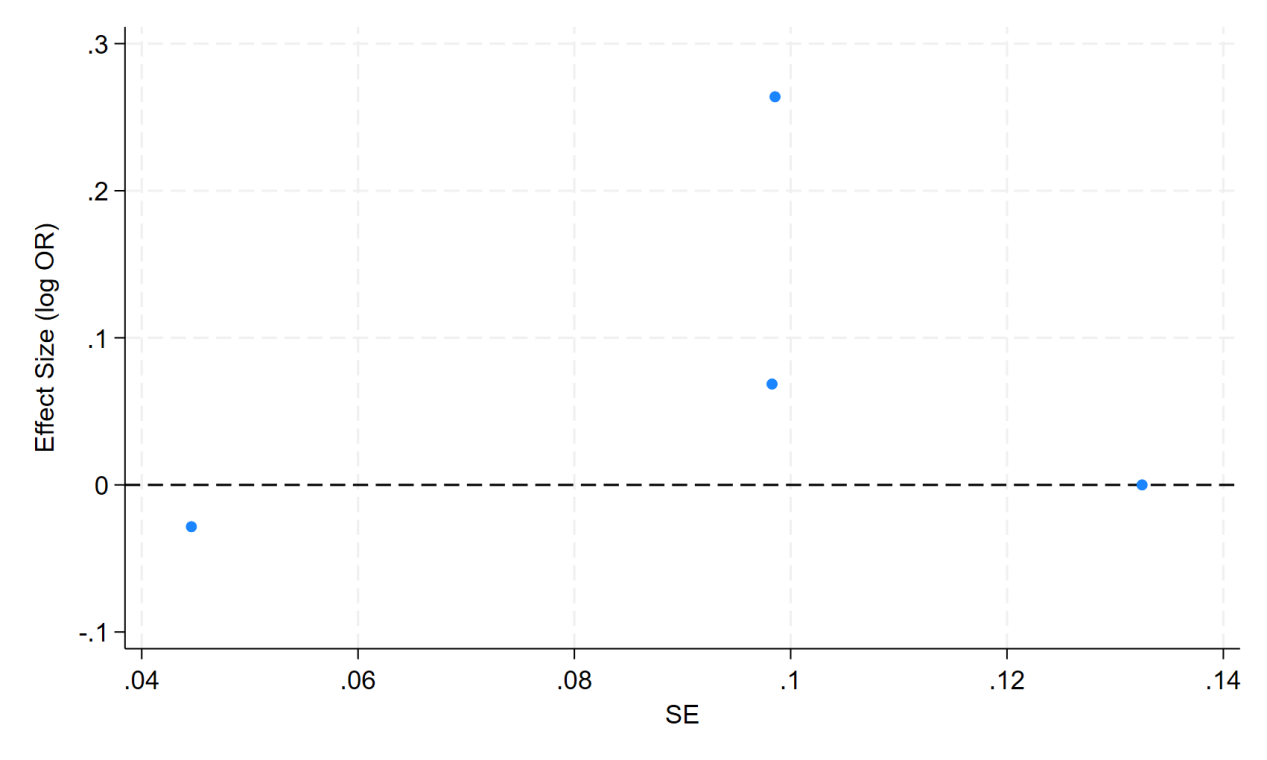


Minerals


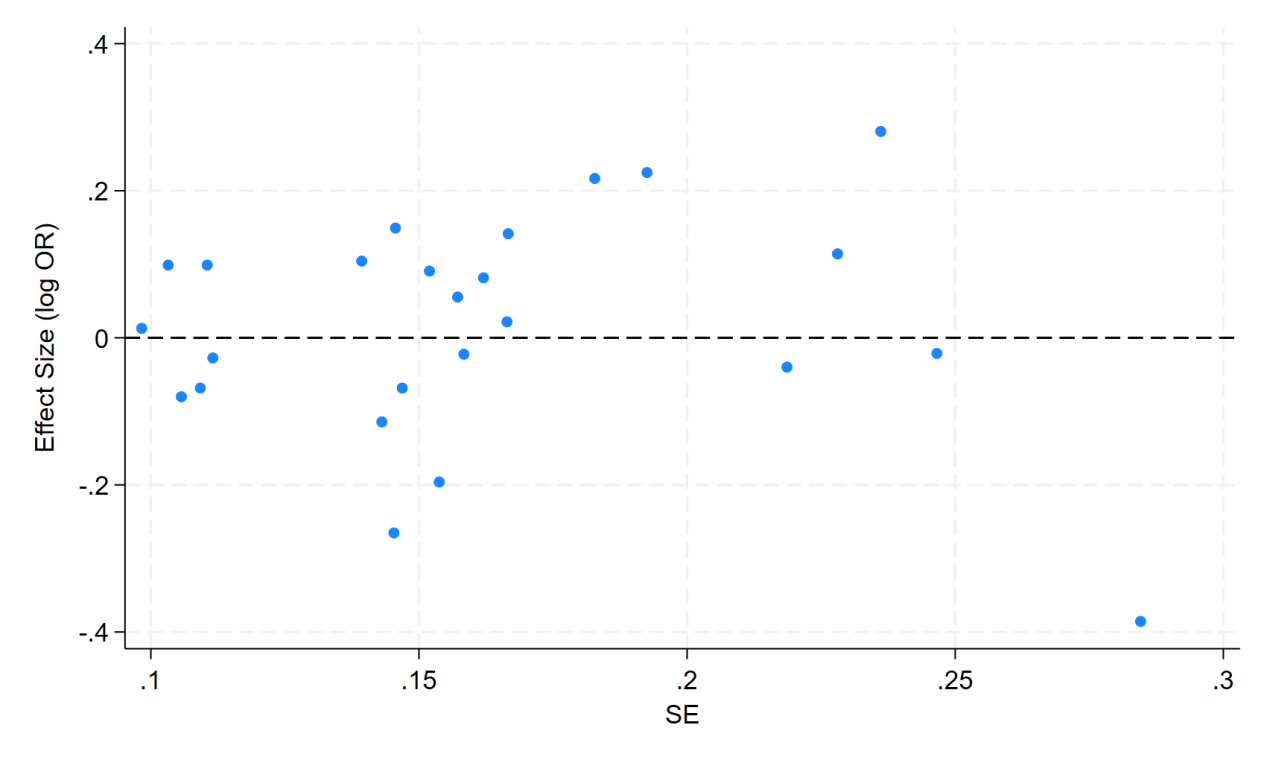


Fat


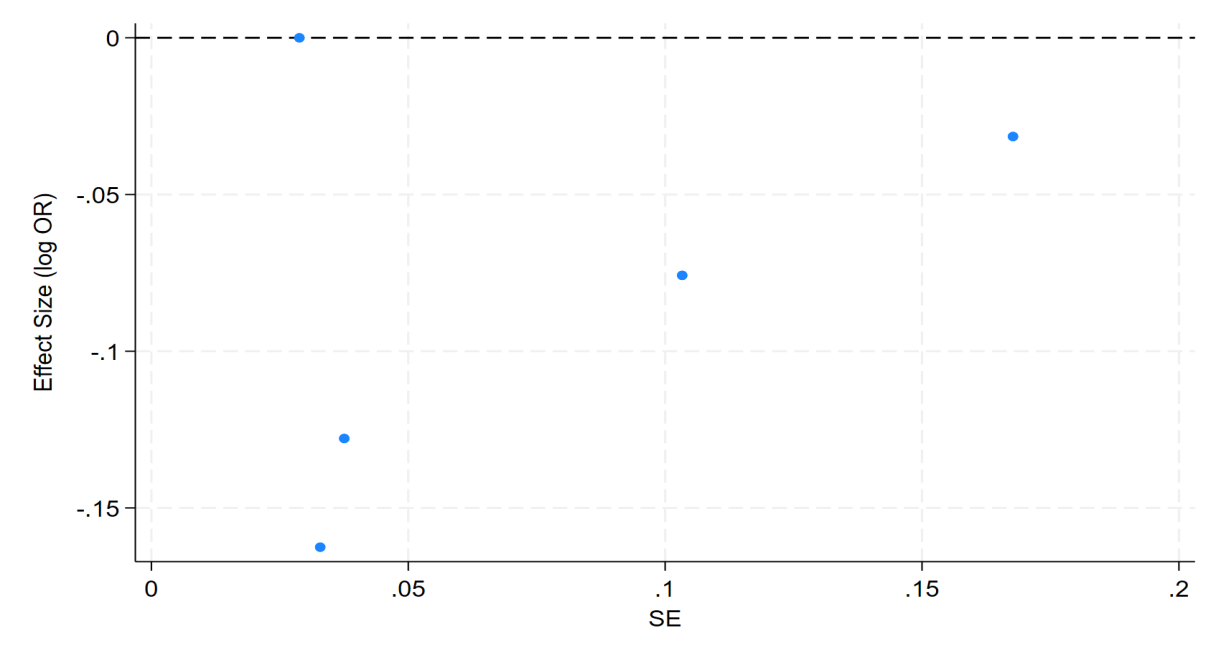


Protein


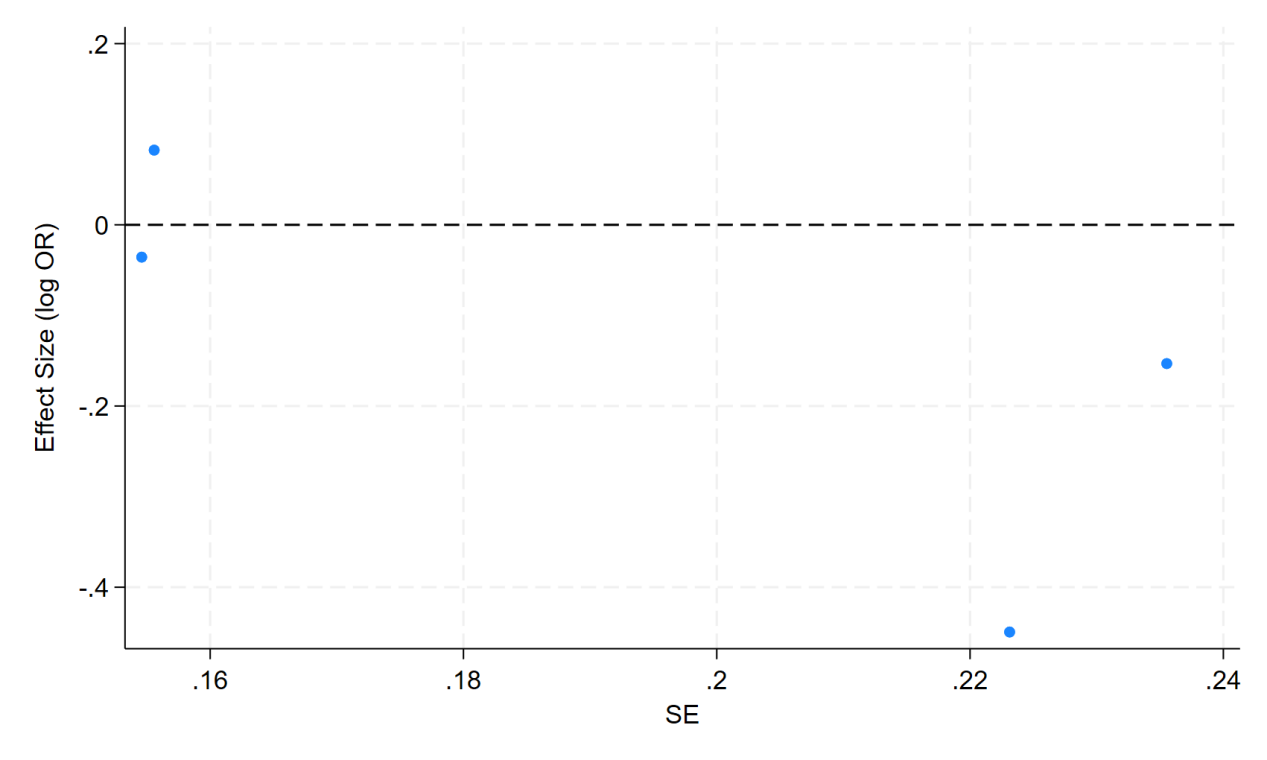


Fiber


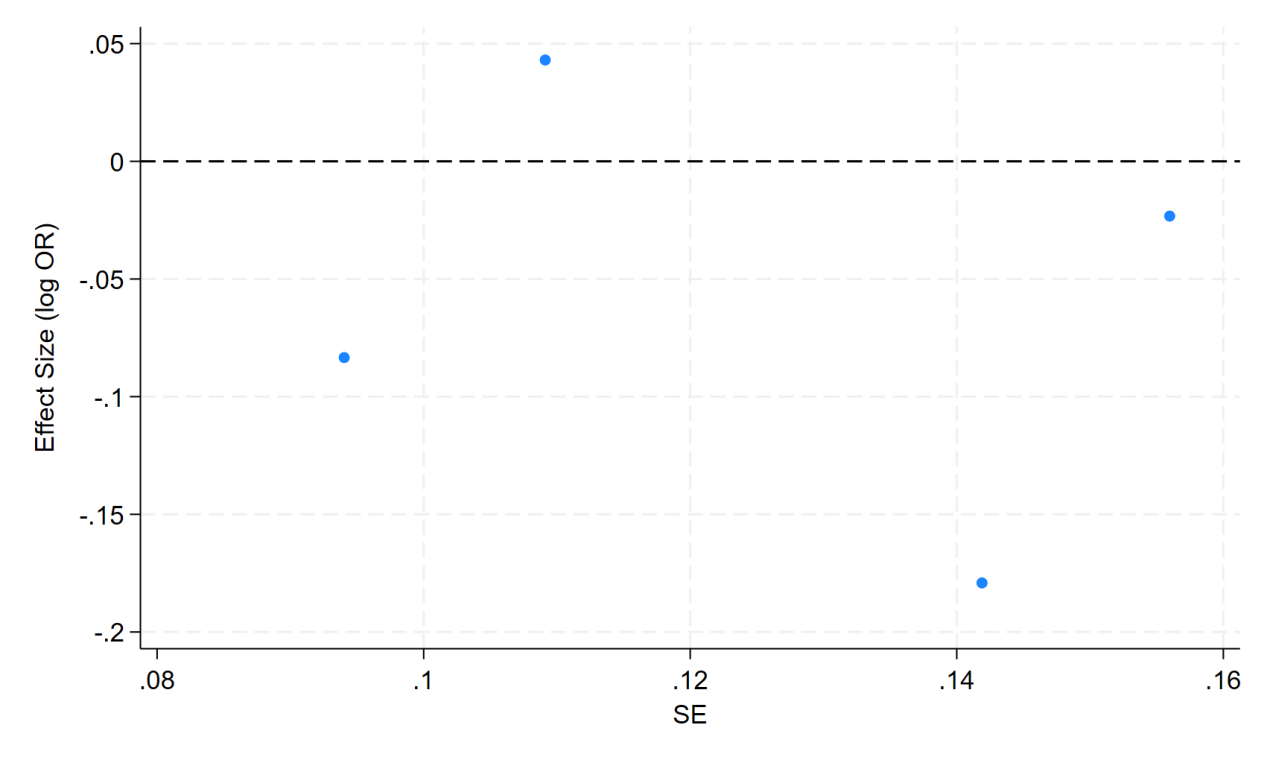


Carbohydrates


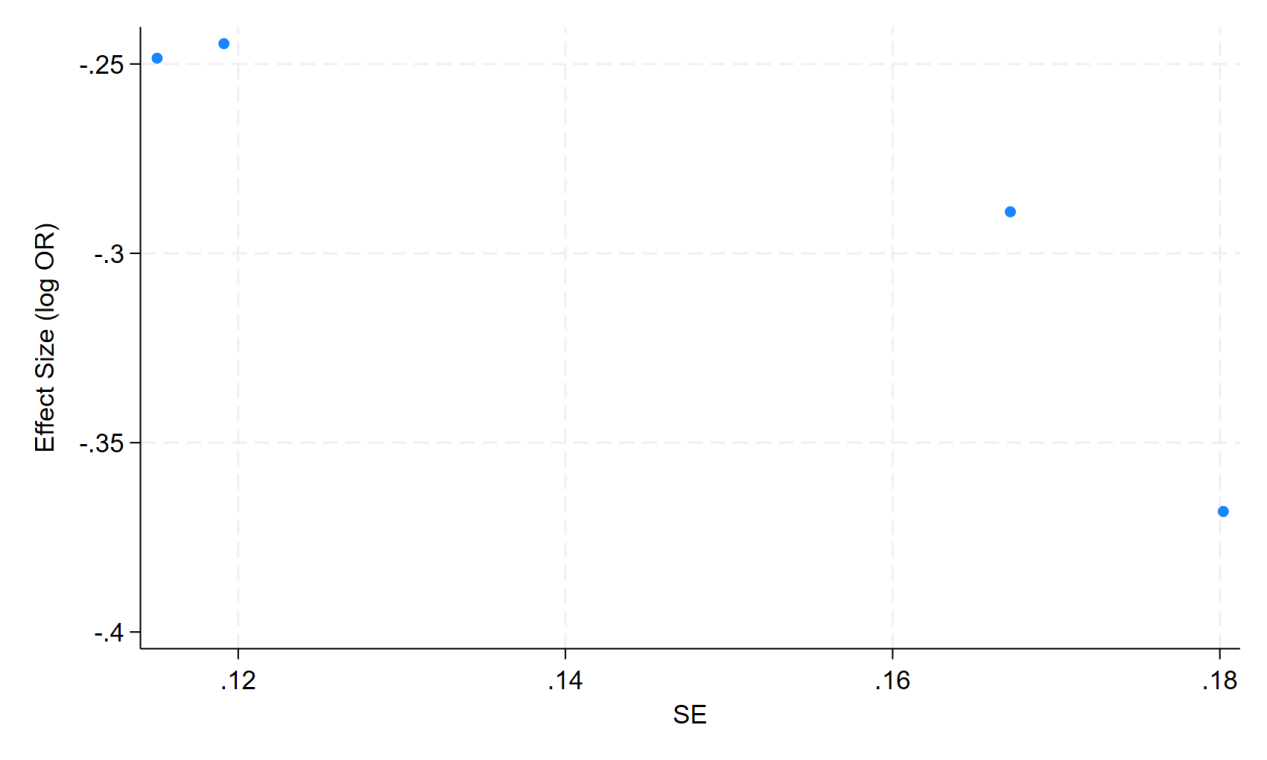


Sugar


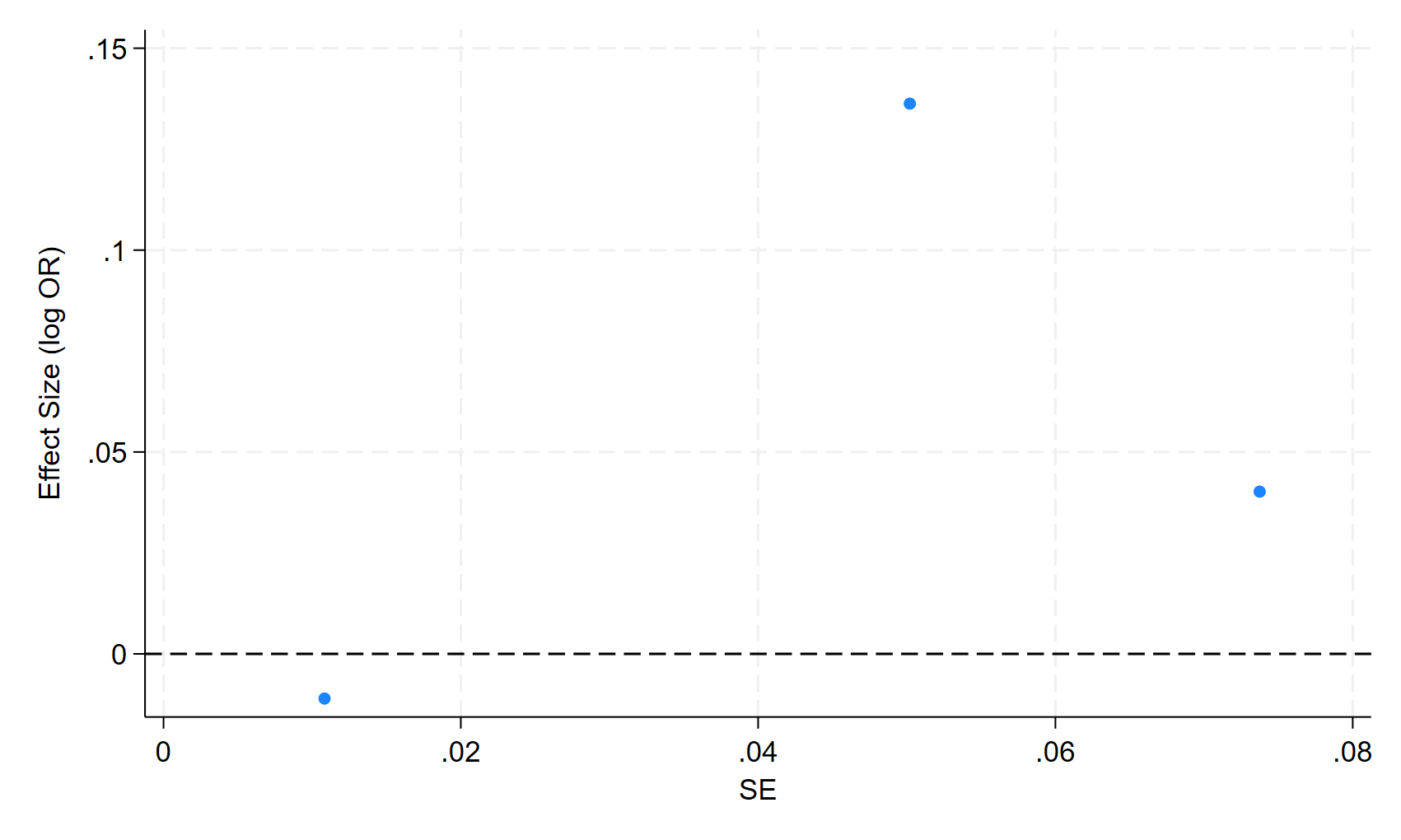


Alcohol


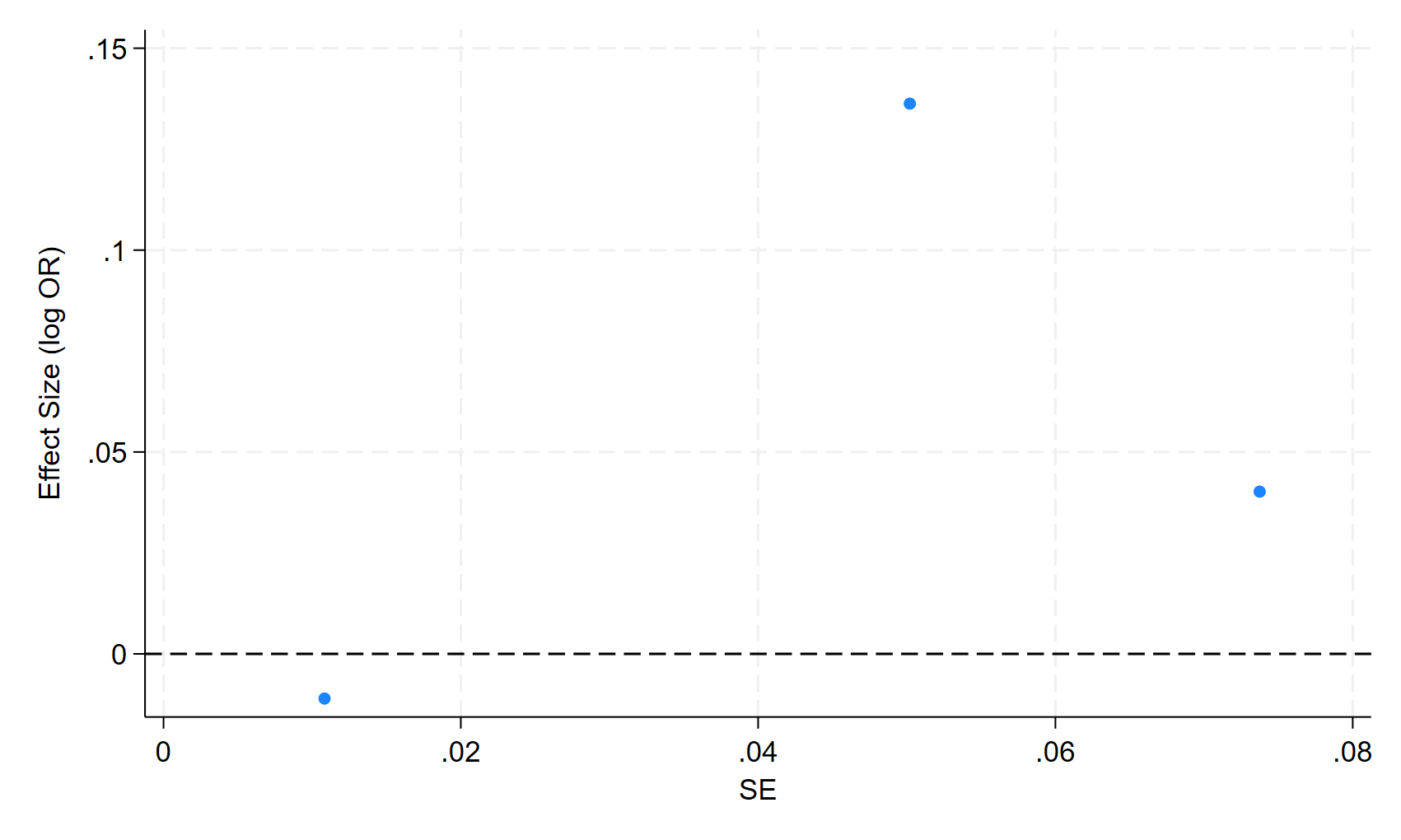


Coffee


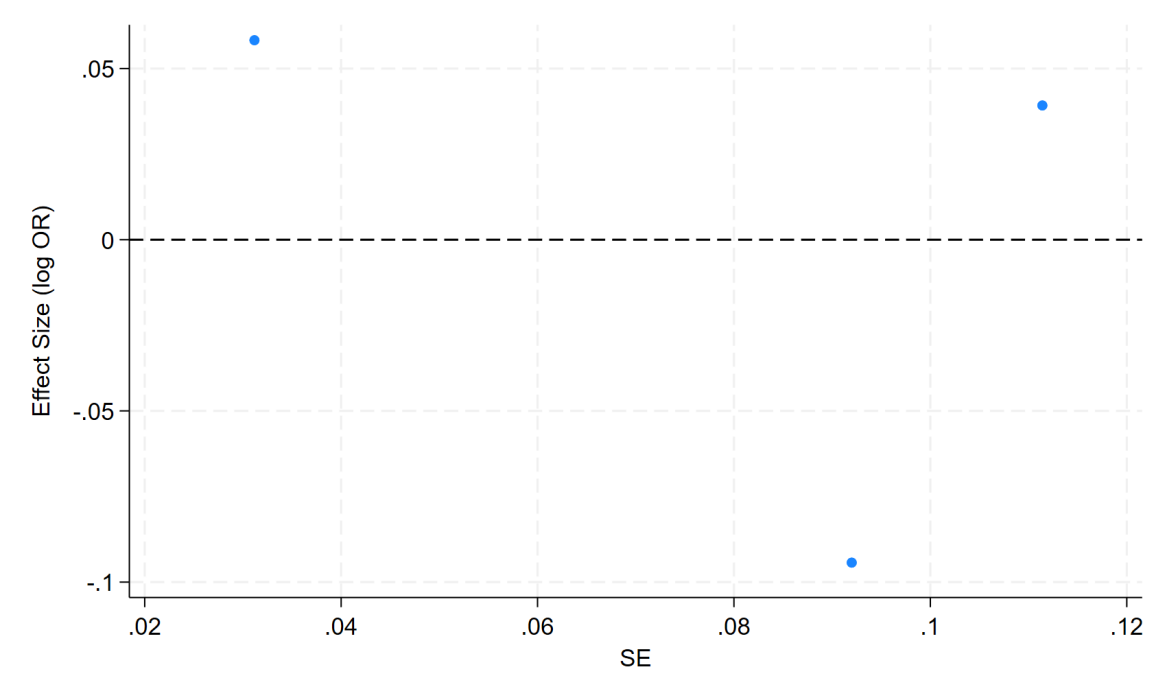


Fish


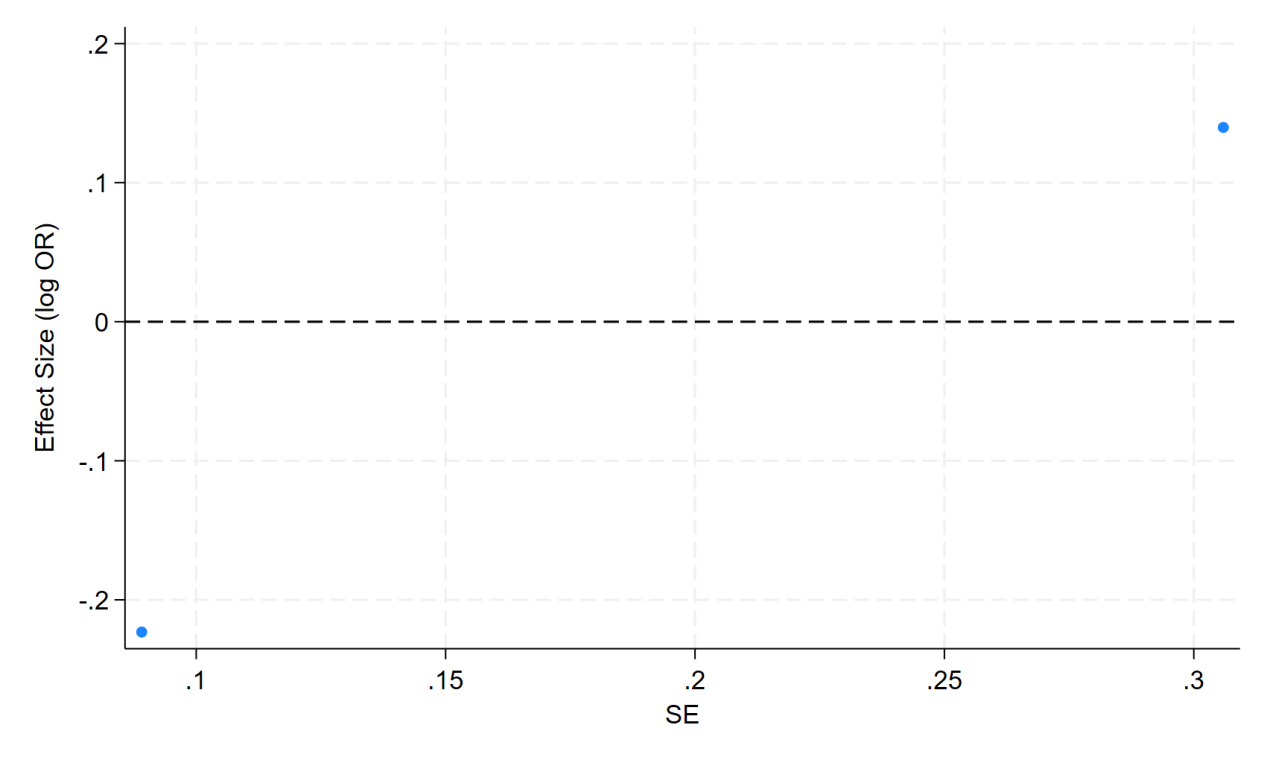

Supplement: Supplementary file 1 [file Data_Sheet_1.zip › 补充文件/Supplement Material 5 Dietary Nutrition and Hearing loss (except ARHL)Meta-analysis of funnel plot.docx]
