# Supplementary material for: Protective effects of dietary nutrients on hearing loss: a systematic review and meta-analysis
Source: Front Nutr. 2025 May 9;12:1528771. doi: 10.3389/fnut.2025.1528771 (PMC12100664; doi:10.3389/fnut.2025.1528771)
Supplement: Supplementary file 1 [file Data_Sheet_1.zip › 补充文件/Supplement Table 3 Search Strategy.docx]

Supplement Table 3: Search Strategy (last literature search performed on 27 July 2024)

|  | PubMed |
| --- | --- |
| #1 | ("diet"[MeSH Terms] OR "Diets"[Title/Abstract] OR "nutritional status"[MeSH Terms] OR "status nutritional"[Title/Abstract] OR "nutrition status"[Title/Abstract] OR "status nutrition"[Title/Abstract] OR "food"[MeSH Terms] OR "Foods"[Title/Abstract] OR "Bread"[Title/Abstract] OR "Candy"[Title/Abstract] OR "Chocolate"[Title/Abstract] OR "Condiments"[Title/Abstract] OR "crops agricultural"[Title/Abstract] OR "dairy products"[Title/Abstract] OR "dietary advanced glycation end products"[Title/Abstract] OR "dietary carbohydrates"[Title/Abstract] OR "dietary sugars"[Title/Abstract] OR "dietary fats"[Title/Abstract] OR "dietary fiber"[Title/Abstract] OR "dietary proteins"[Title/Abstract] OR "dietary supplements"[Title/Abstract] OR "edible insects"[Title/Abstract] OR "edible insects"[Title/Abstract] OR "fast foods"[Title/Abstract] OR "Fruit"[Title/Abstract] OR "Meat"[Title/Abstract] OR "meat substitutes"[Title/Abstract] OR "Nuts"[Title/Abstract] OR "Vegetables"[Title/Abstract] OR "nutrients"[MeSH Terms] OR "Nutrient"[Title/Abstract] OR "Macronutrients"[Title/Abstract] OR "Macronutrient"[Title/Abstract] OR "vitamins"[MeSH Terms] OR "Vitamin"[Title/Abstract] OR "alcohols"[MeSH Terms] OR "Beverages"[MeSH Terms] OR "Beverage"[Title/Abstract] OR "Coffee"[Title/Abstract] OR "alcoholic beverages"[Title/Abstract] OR "Tea"[Title/Abstract] OR "drinks"[Title/Abstract]) |
| #2 | "hearing loss"[MeSH Terms] OR "hearing impairment"[Title/Abstract] OR "Deafness"[MeSH Terms] OR "hearing damage"[Title/Abstract] OR "hearing loss, noise induced"[MeSH Terms] OR "noise induced hearing loss"[Title/Abstract] OR "hearing loss, sudden"[MeSH Terms] OR "sudden deafness"[Title/Abstract] OR "sudden hearing loss"[Title/Abstract] OR "hypacusia"[Title/Abstract] OR "hearing tests"[MeSH Terms] OR "hearing test"[Title/Abstract] OR "test hearing"[Title/Abstract] OR "impaired hearing"[Title/Abstract] OR "age related hearing impairment"[Title/Abstract] |
| #3 | Humans[Filter] |
| #4 | English[Filter] |
| #5 | #1 AND #2 AND #3 AND #4 |
