# Supplementary material for: Protective effects of dietary nutrients on hearing loss: a systematic review and meta-analysis
Source: Front Nutr. 2025 May 9;12:1528771. doi: 10.3389/fnut.2025.1528771 (PMC12100664; doi:10.3389/fnut.2025.1528771)
Supplement: Supplementary file 1 [file Data_Sheet_1.zip › 补充文件/Supplement Table 6 Main results of meta-analysis of dietary nutrition and ARHL.docx]

**Supplement Table 5 Main results of meta-analysis of dietary nutrition and ARHL**

| **Food**  **/nutrient type** | **Meta-Analysis Results** | **subgroup** | **number of studies** | **pooled OR** | **95 Confidence Interval** | **I2(%)** | **p Value** | **egger's test** | **Sensitivity**  **analyses** |
| --- | --- | --- | --- | --- | --- | --- | --- | --- | --- |
| **micronutrients** | Vitamin A | Vitamin A | 3 | 0.86 | 0.67–1.09 | 0 | 0.973 | p=0.550 | Stable |
|  |  | Retinol | 2 | 0.81 | 0.57–1.15 | 84 | 0.012 | p=0.220 | Stable |
|  |  | Overall | 5 | 0.84 | 0.70–1.01 | 52.8 | 0.096 | p=0.549 | Stable |
|  | Vitamin B | Vitamin B12 | 3 | 0.85 | 0.60–1.20 | 35 | 0.320 | 0.450 | Stable |
|  |  | Vitamin B3 | 4 | 1.10 | 0.95–1.28 | 0 | 0.150 | 0.620 | Stable |
|  |  | Vitamin B9 | 2 | 0.70 | 0.40–1.22 | 75 | 0.098 | 0.210 | Stable |
|  | ​ | ​Overall | 9 | 0.95 | 0.80–1.13 | 52 | 0.480 | 0.550 | Stable |
|  | Vitamin E | NA | 4 | 1.038 | 0.893–1.207 | 0 | 0.624 | 0.214 | Stable |
|  | Carotene | Carotene | 2 | 1.030 | 0.933–1.137 | 0 | 0.559 | 0.428 | Stable |
|  | ​ | ​β-Carotene | 3 | 1.004 | 0.898–1.122 | 0 | 0.944 | 0.616 | Stable |
|  | ​ | ​Overall | 5 | 1.018 | 0.946–1.096 | 0 | 0.628 | 0.428 | Stable |
|  | ​Carotenoid | ​β-Cryptoxanthin | 2 | 0.926 | 0.867–0.990 | 35.10 | 0.024 | 0.597 | Stable |
|  |  | ​Lycopene | 2 | 0.951 | 0.871–1.039 | 65.00 | 0.267 | 0.958 | Stable |
|  | ​ | ​Xanthophyll | 2 | 0.958 | 0.900–1.019 | 0 | 0.174 | - | Stable |
|  | ​ | ​Renieratene | 1 | 0.84 | 0.787–0.897 | - | <0.001 | - | NA |
|  |  | ​Overall | 7 | 0.928 | 0.885–0.972 | 63.10 | 0.002 | - | Stable |
|  | ​Mineral | ​Magnesium | 1 | 1.07 | 0.935–1.224 | 0 | 0.324 | - | NA |
|  | ​ | ​Calcium | 2 | 1.014 | 0.842–1.220 | 50.30 | 0.887 | - | Stable |
|  |  | ​Iron | 2 | 0.921 | 0.828–1.025 | 0 | 0.133 | - | Stable |
|  | ​ | ​Potassium | 3 | 1.015 | 0.753–1.369 | 78.50 | - | - | Stable |
|  |  | ​Zinc | 1 | 1.16 | 0.620–2.170 | - | - | - | NA |
|  | ​ | ​Phosphorus | 2 | 0.806 | 0.662–0.982 | 0 | - | - | Stable |
|  | ​ | ​Sodium | 1 | 0.77 | 0.589–1.007 | - | - | - | NA |
|  | ​ | ​Overall | 12 | 0.95 | 0.870–1.037 | 54.80 | 0.252 | 0.729 | Stable |
| **Macronutrients** | ​fat | ​Non-fatty acids | 6 | 0.938 | 0.836–1.052 | 26.70 | 0.274 | - | Stable |
|  | ​ | ​Saturated fat | 5 | 1.084 | 0.957–1.229 | 14.80 | 0.205 | - | Stable |
|  | ​ | ​Trans fat | 3 | 1.209 | 0.788–1.855 | ​81.5 | 0.385 | - | Stable |
|  |  | ​Fat (unspecified) | 7 | 0.913 | 0.837–0.997 | 3.00 | 0.042 | - | Stable |
|  |  | ​Overall | 21 | 0.986 | 0.913–1.065 | 47.90 | 0.714 | 0.425 | Stable |
|  | Fiber | NA | 6 | 0.882 | 0.760–1.022 | 24.80 | ​0.095 | 0.920 | Stable |
|  | Protein | NA | 5 | 0.858 | 0.789–0.934 | ​0 | <0.001 | p=0.018 | Stable |
|  | Carbohydrates(Sugar) | NA | 5 | 0.858 | 0.789–0.934 | ​0 | ​<0.001 | p=0.018 | Stable |
| **Beverages** | Alcohol | NA | 6 | 0.905 | 0.686–1.194 | 86.60 | 0.48 | 0.469 | Stable |
|  | Tea | NA | 5 | 0.887 | 0.800–0.983 | 26.10 | 0.022 | 0.672 | Stable |
| ****Food**** | **FIsh** | NA | **2** | **0.958** | **0.763–1.203** | **​0** | **0.714** | **N/A** | Stable |
