# Supplementary material for: Protective effects of dietary nutrients on hearing loss: a systematic review and meta-analysis
Source: Front Nutr. 2025 May 9;12:1528771. doi: 10.3389/fnut.2025.1528771 (PMC12100664; doi:10.3389/fnut.2025.1528771)
Supplement: Supplementary file 1 [file Data_Sheet_1.zip › 补充文件/Supplement Table 7 Main results of meta-analysis of dietary nutrition and Hearing loss (except ARHL).docx]

**Supplement Table 6 Main results of meta-analysis of dietary nutrition and Hearing loss (except ARHL)**

| **Food**  **/nutrient type** | **MetaNSAnalysis Results** | **subgroup** | **number of studies** | **pooled OR** | **95 Confidence Interval** | **I^2^(%)** | **p Value** | **egger's test** | **Sensitivity**  **analyses** |
| --- | --- | --- | --- | --- | --- | --- | --- | --- | --- |
| **micronutrients** | Vitamin A | ​Vitamin A | 5 | 1.050 | 0.976–1.129 | ​0.0 | 0.19 | NS | Stable |
|  |  | ​Retinol | 3 | 1.035 | 0.867–1.235 | ​65.9 | 0.702 | NS | Stable |
|  |  | ​Overall | 8 | 1.053 | 0.991–1.119 | ​2.6 | 0.098 | 0.366 | Stable |
|  | Vitamin B | Vitamin B6 | 1 | 1.009 | 0.923–1.104 | 0 | 0.845 | NS | NS |
|  |  | ​Vitamin B1 | 3 | 0.925 | 0.773–1.106 | 43.70 | 0.391 | NS | Stable |
|  |  | ​Vitamin B9 | 1 | 1.014 | 0.928–1.108 | 0 | 0.759 | NS | NS |
|  |  | ​Vitamin B3 | 1 | 0.970 | 0.885–1.063 | 0 | 0.515 | NS | NS |
|  |  | ​Overall | 6 | 0.990 | 0.943–1.039 | ​0 | 0.675 | 0.267 | Stable |
|  | Vitamin C | NA | 9 | 0.988 | 0.896 -1.088 | 58.7 | 0.804 | 0.964 | Stable |
|  | Carotene | Carotene | 4 | 0.982 | 0.910–1.060 | 0.00 | 0.639 | 0.961 | Stable |
|  |  | ​βNSCarotene | 3 | 0.914 | 0.832–1.004 | 48.80 | ​0.059 | NS | Stable |
|  |  | ​αNSCarotene | 1 | 1.01 | 0.929–1.098 | NS | 0.816 | NS | NS |
|  |  | ​Overall | 8 | 0.958 | 0.909–1.010 | 28.90 | 0.115 | 0.961 | Stable |
|  | Minerals | Magnesium | 1 | 0.68 | 0.389–1.188 | NS | 0.175 | NS | NS |
|  |  | ​Calcium | 4 | 0.968 | 0.861–1.088 | ​0.0 | 0.585 | 0.785 | Stable |
|  |  | ​Iron | 4 | 1.054 | 0.934–1.189 | ​0.0 | 0.398 | NS | Stable |
|  |  | ​Potassium | 4 | 1.133 | 0.962–1.334 | ​0.0 | 0.134 | NS | Stable |
|  |  | ​Phosphorus | 4 | 0.971 | 0.837–1.126 | 26.30 | 0.696 | NS | Stable |
|  |  | ​Ash | 4 | 1.084 | 0.899–1.306 | ​0.0 | 0.397 | NS | Stable |
|  |  | ​Sodium | 4 | 0.955 | 0.808–1.130 | ​0.0 | 0.594 | NS | Stable |
|  |  | ​Overall | 25 | 1.012 | 0.956–1.071 | ​0.0 | 0.674 | 0.886 | Stable |
|  |  | Magnesium | 1 | 0.68 | 0.389–1.188 | NS | 0.175 | NS | Stable |
| **Macronutrients** | Fat | NonNSfatty acids | 3 | 0.909 | 0.819–1.009 | 87.20 | NS | 0.732 | Stable |
|  |  | ​Fat (unspecified) | 2 | 0.938 | 0.790–1.115 | 0.00 | NS | 0.491 | Stable |
|  |  | ​Overall | 5 | ​0.915 | 0.840–0.996 | 74.50 | 0.041 | NS | Stable |
|  | Protein | ​Overall | 4 | 0.909 | 0.737–1.122 | ​25.2 | 0.374 | 0.185 | Stable |
|  | Fiber | NA | 4 | 0.953 | 0.854 -1.060 | 0 | 0.636 | 0.792 | Stable |
|  | Carbohydrates | NA | 5 | ​0.915 | 0.840–0.996 | ​74.5 | ​0.041 | 0.491 | Stable |
|  | Carbohydrates(Sugar) | NA | 3 | 1.049 | 0.948–1.162 | ​76.7 | 0.356 | 0.351 | Stable |
| **Beverages** | Alcohol | ​NA | 3 | 1.031 | 0.952–1.116 | ​19.0 | 0.455 | 0.689 | Stable |
|  | Coffee | NA | 5 | 0.977 | 0.560–1.705 | ​70.7 | 0.936 | 0.595 | Stable |
| **Food** | Fish | NA | 2 | 0.852 | 0.651–1.117 | ​22.9 | 0.247 | NS | Stable |
