# Supplementary material for: Protective effects of dietary nutrients on hearing loss: a systematic review and meta-analysis
Source: Front Nutr. 2025 May 9;12:1528771. doi: 10.3389/fnut.2025.1528771 (PMC12100664; doi:10.3389/fnut.2025.1528771)
Supplement: Supplementary file 1 [file Data_Sheet_1.zip › 补充文件/Supplement Table 8 Forest Plot of Meta-analysis on Other Dietary Nutrients.docx]

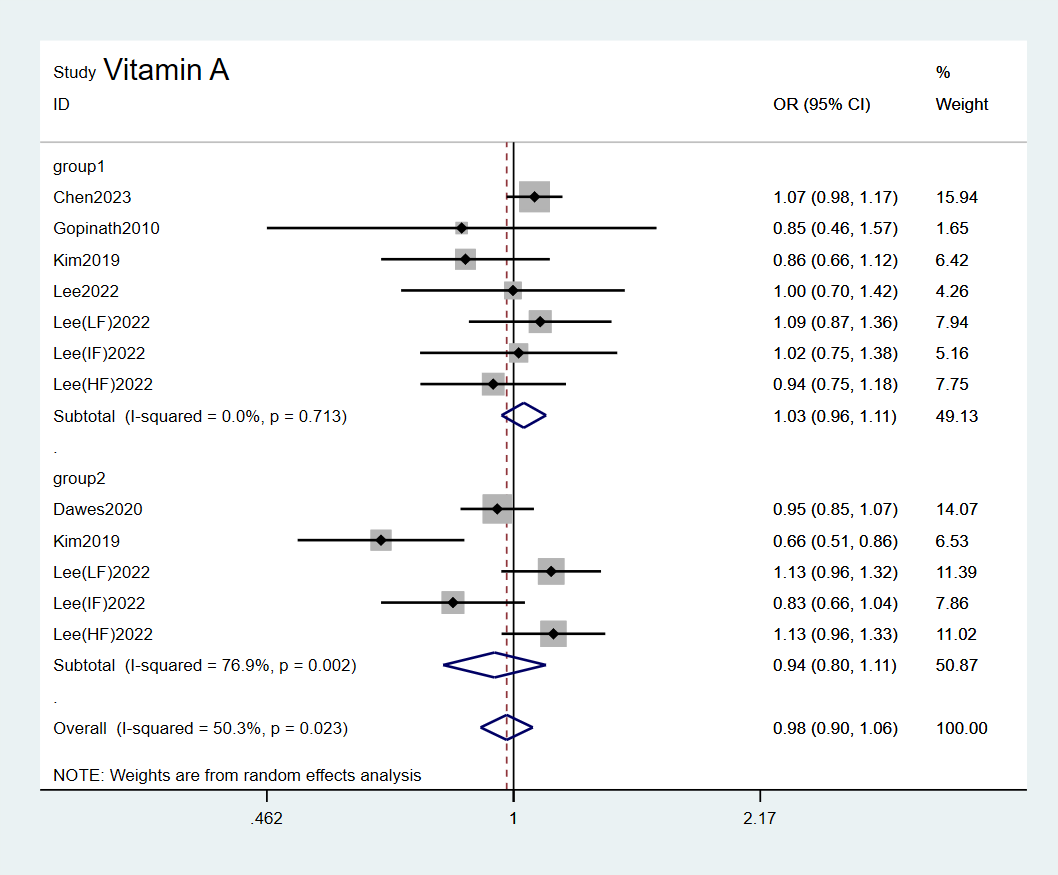


Figure S1 Forest maps for Vitamin A intake and incidence of hearing loss


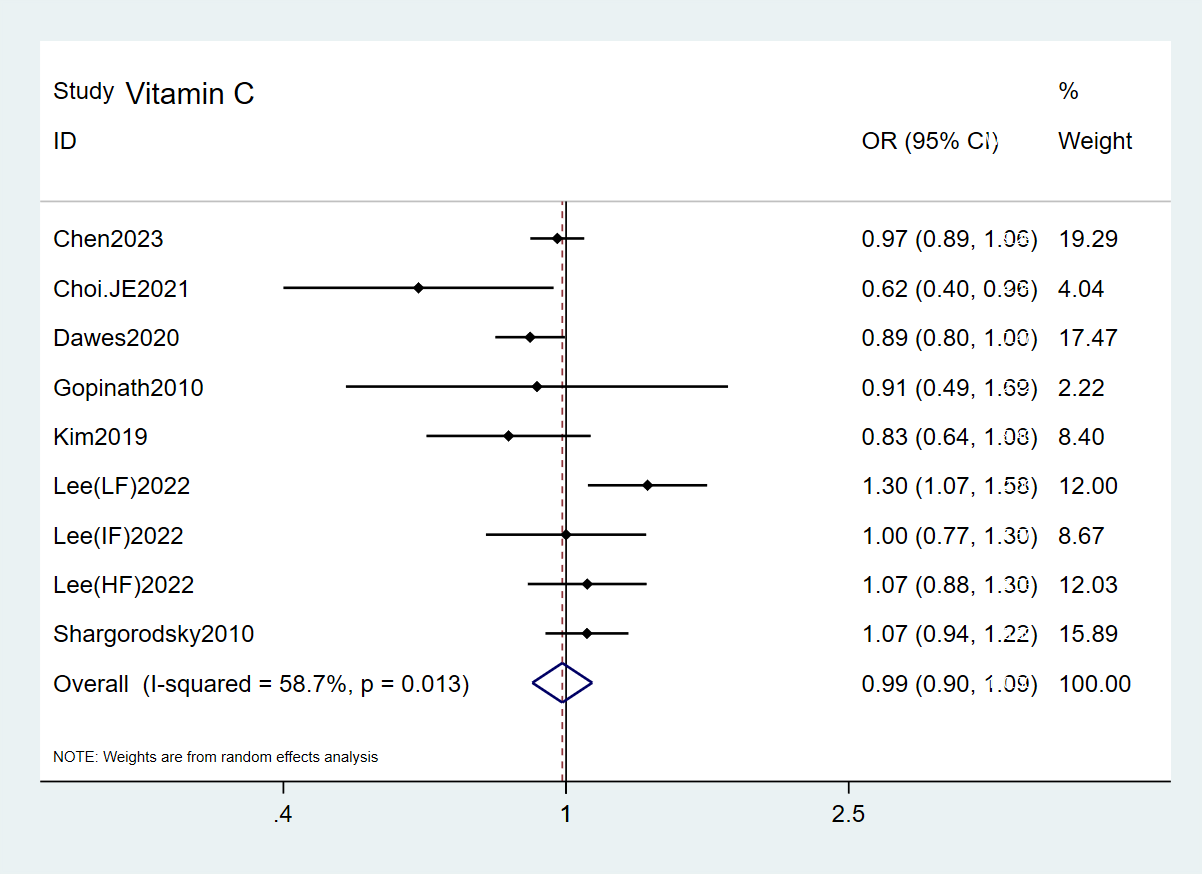


Figure S2 Forest maps for Vitamin C intake and incidence of hearing loss


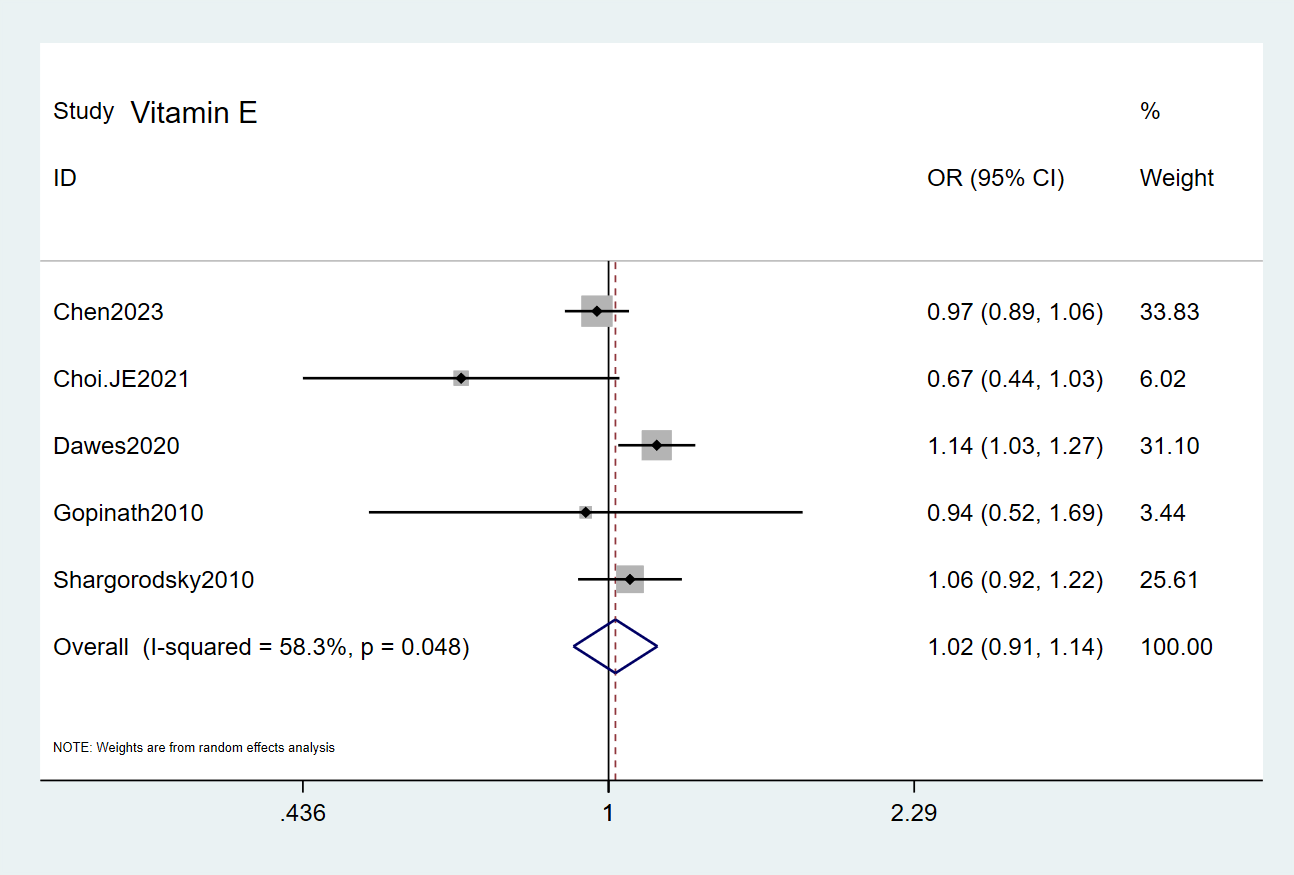


Figure S3 Forest maps for Vitamin E intake and incidence of hearing loss


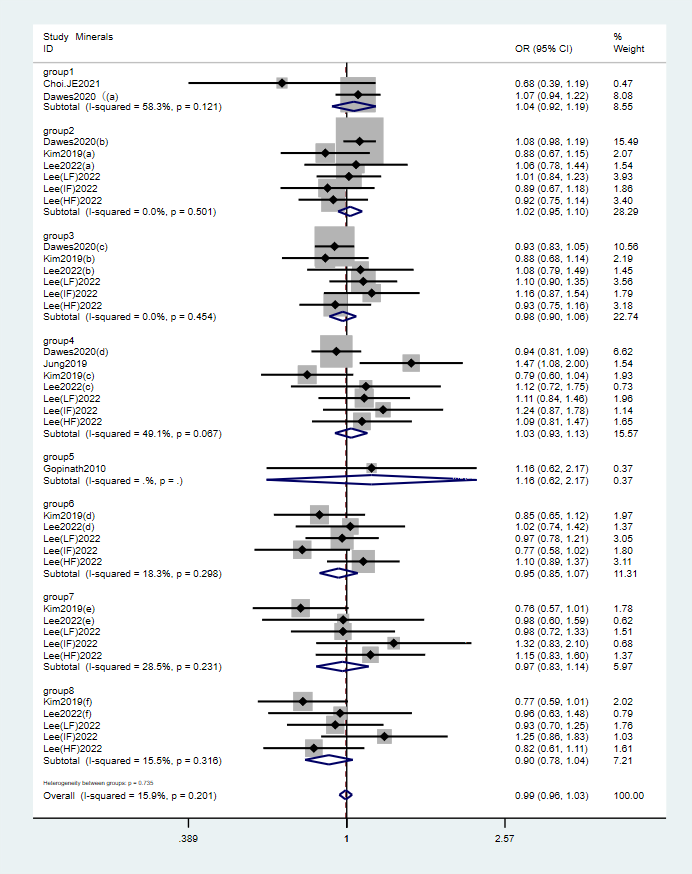


Figure S4 Forest maps for Minerals intake and incidence of hearing loss


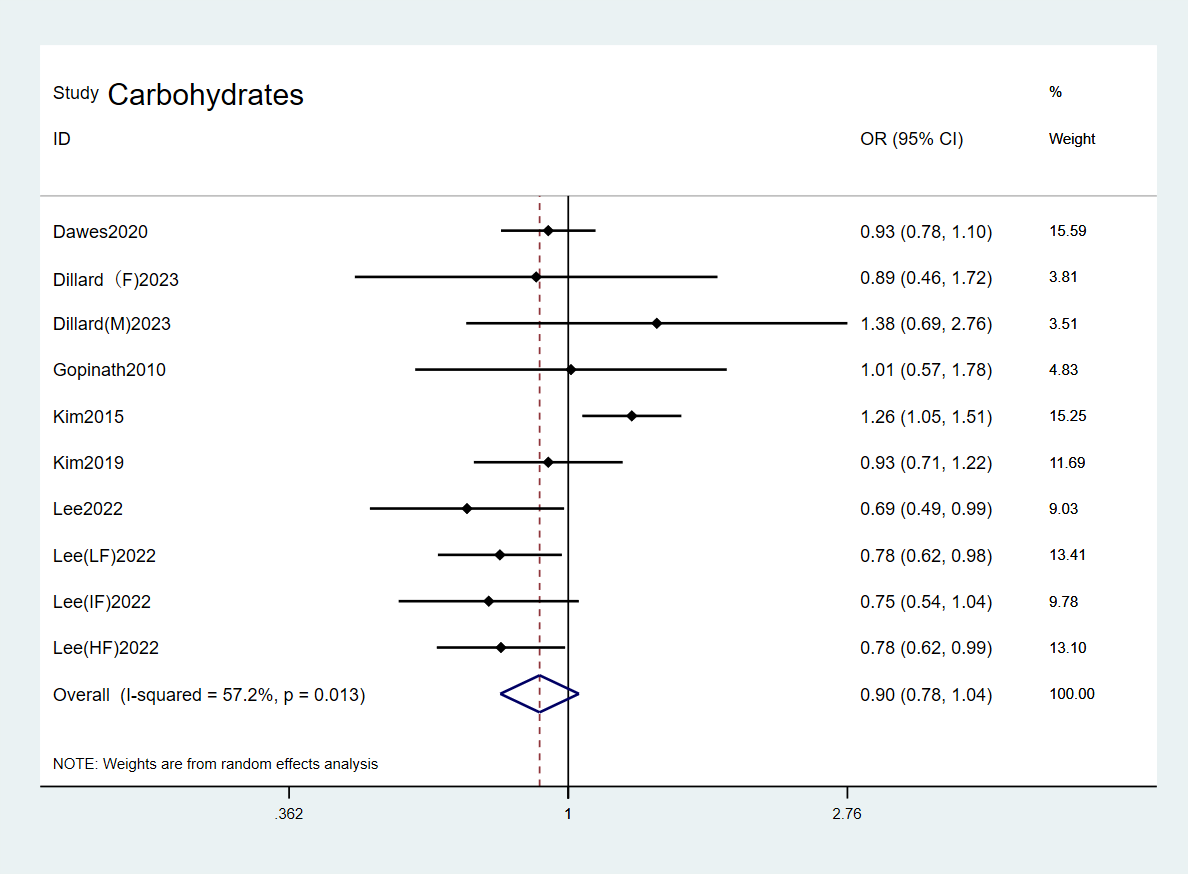


Figure S5 Forest maps for Carbohydrates intake and incidence of hearing loss


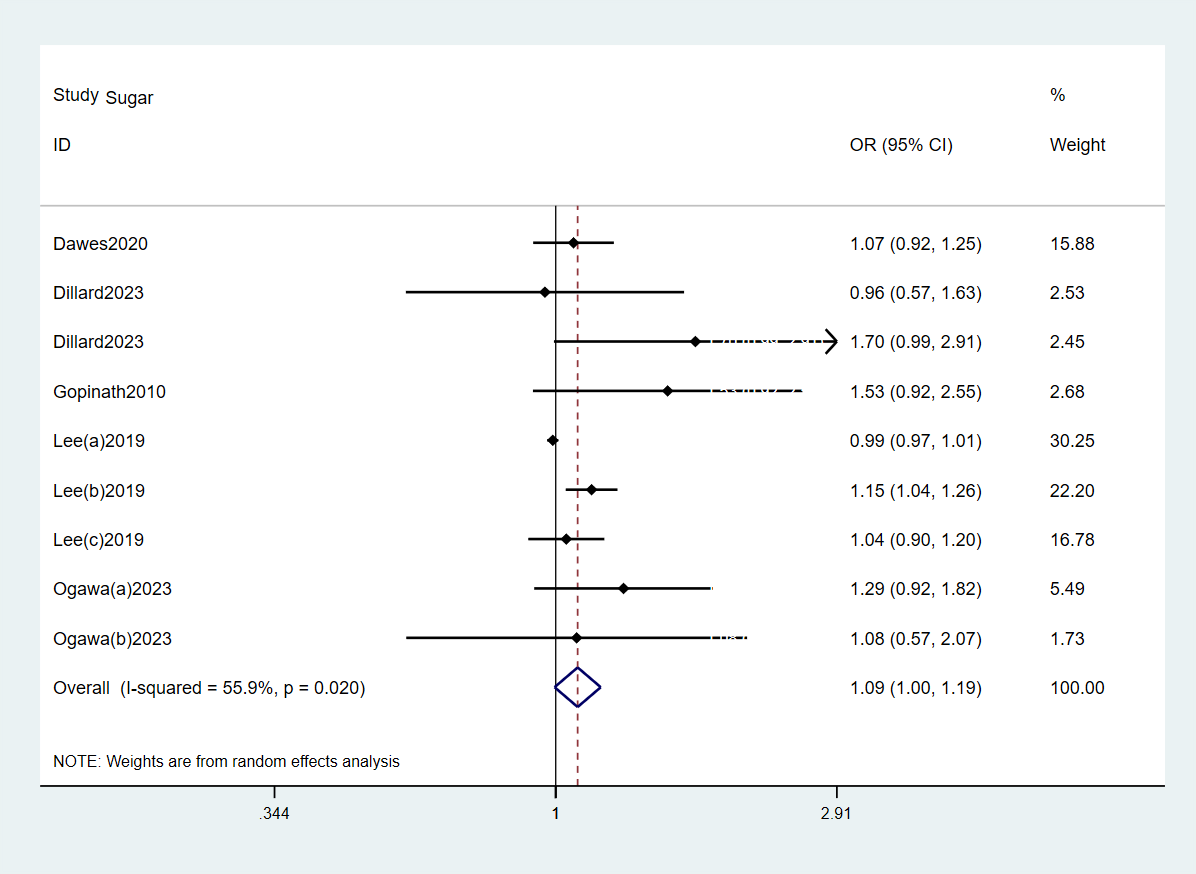


Figure S6 Forest maps for Sugar intake and incidence of hearing loss


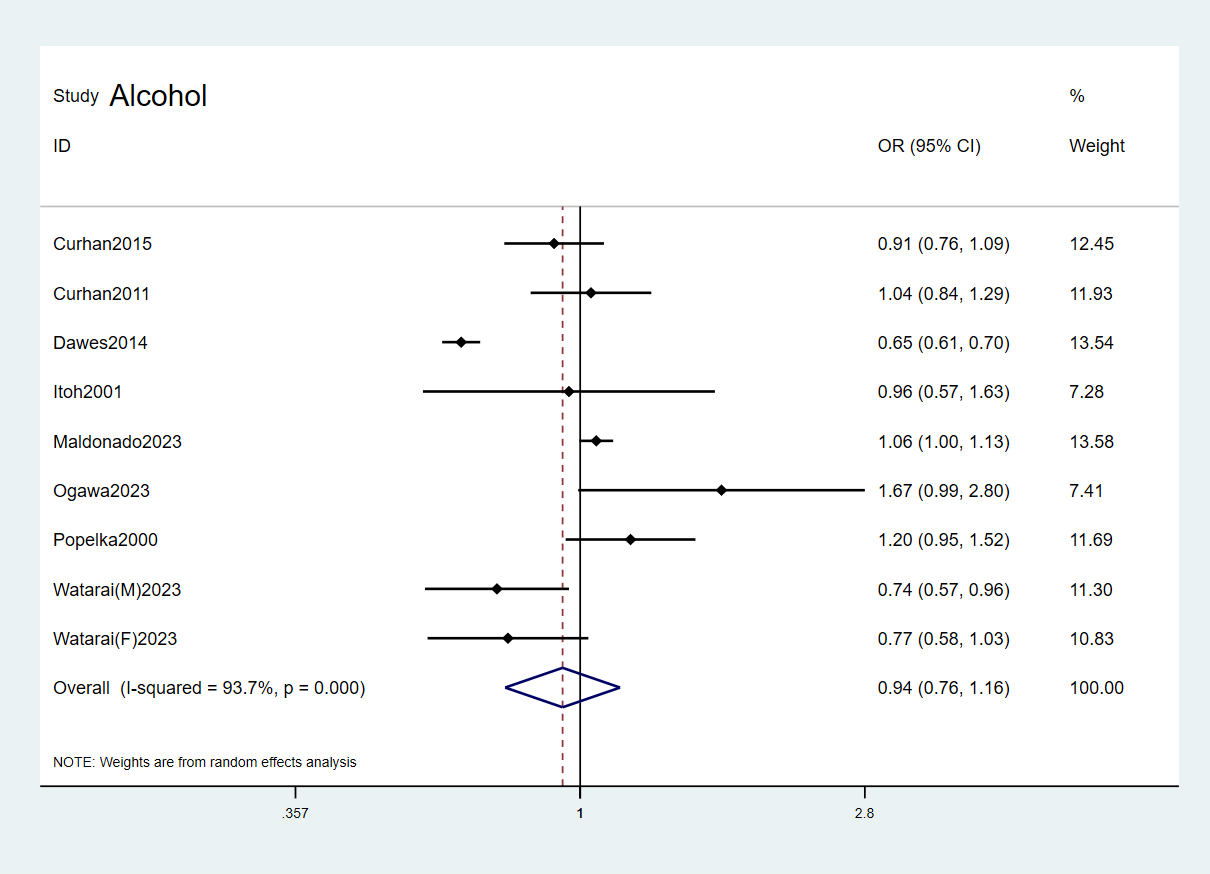


Figure S7 Forest maps for Alcohol intake and incidence of hearing loss


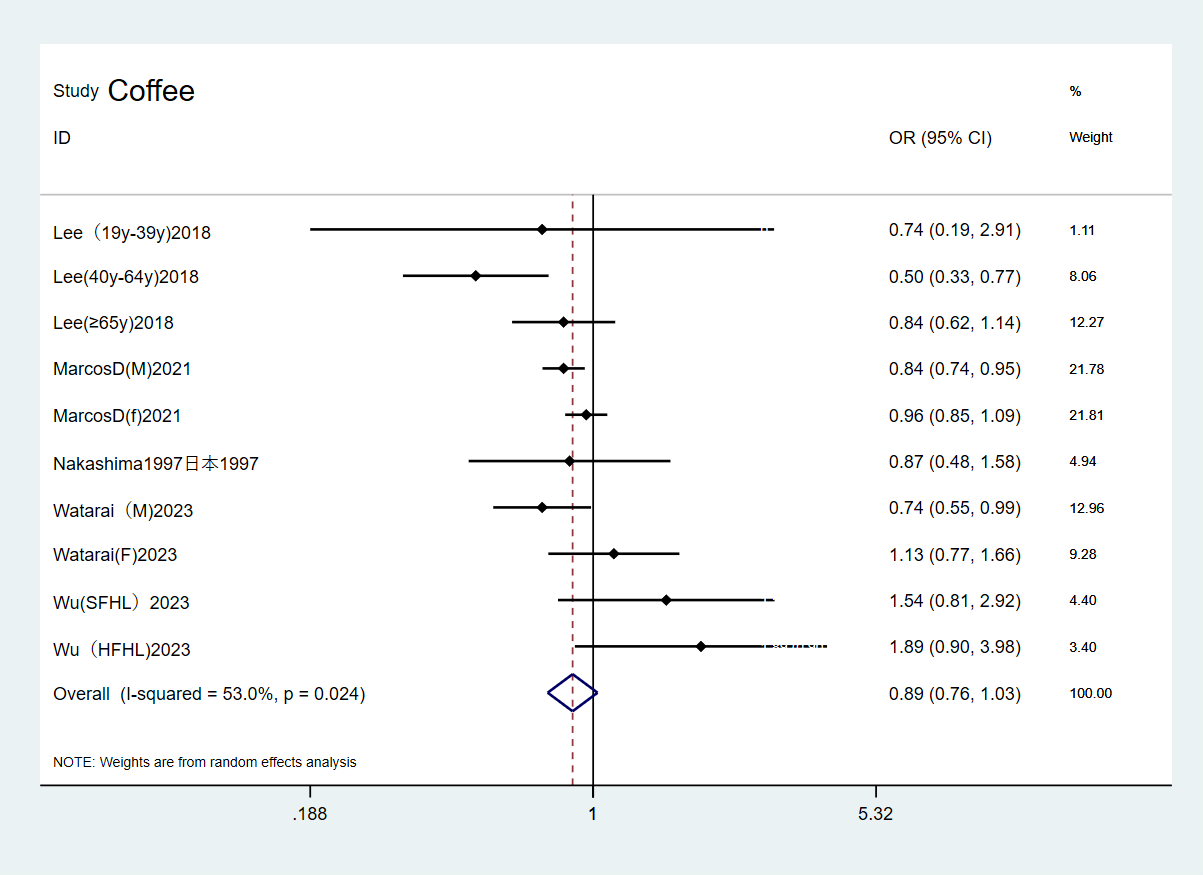


Figure S8 Forest maps for Coffee intake and incidence of hearing loss


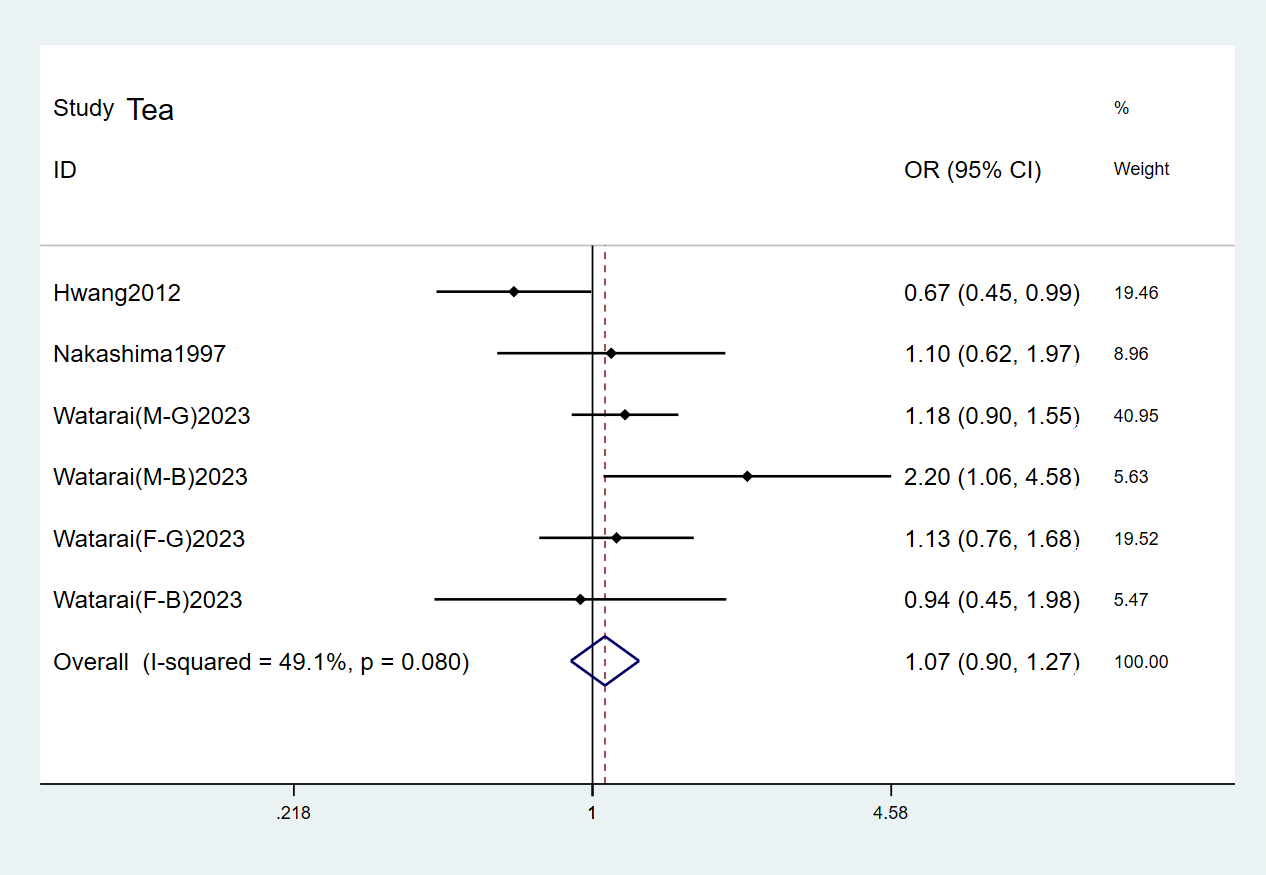


Figure S9 Forest maps for Tea intake and incidence of hearing loss
