# Supplementary figures and images for: Protective effects of dietary nutrients on hearing loss: a systematic review and meta-analysis
Source: Front Nutr. 2025 May 9;12:1528771. doi: 10.3389/fnut.2025.1528771 (PMC12100664; doi:10.3389/fnut.2025.1528771)

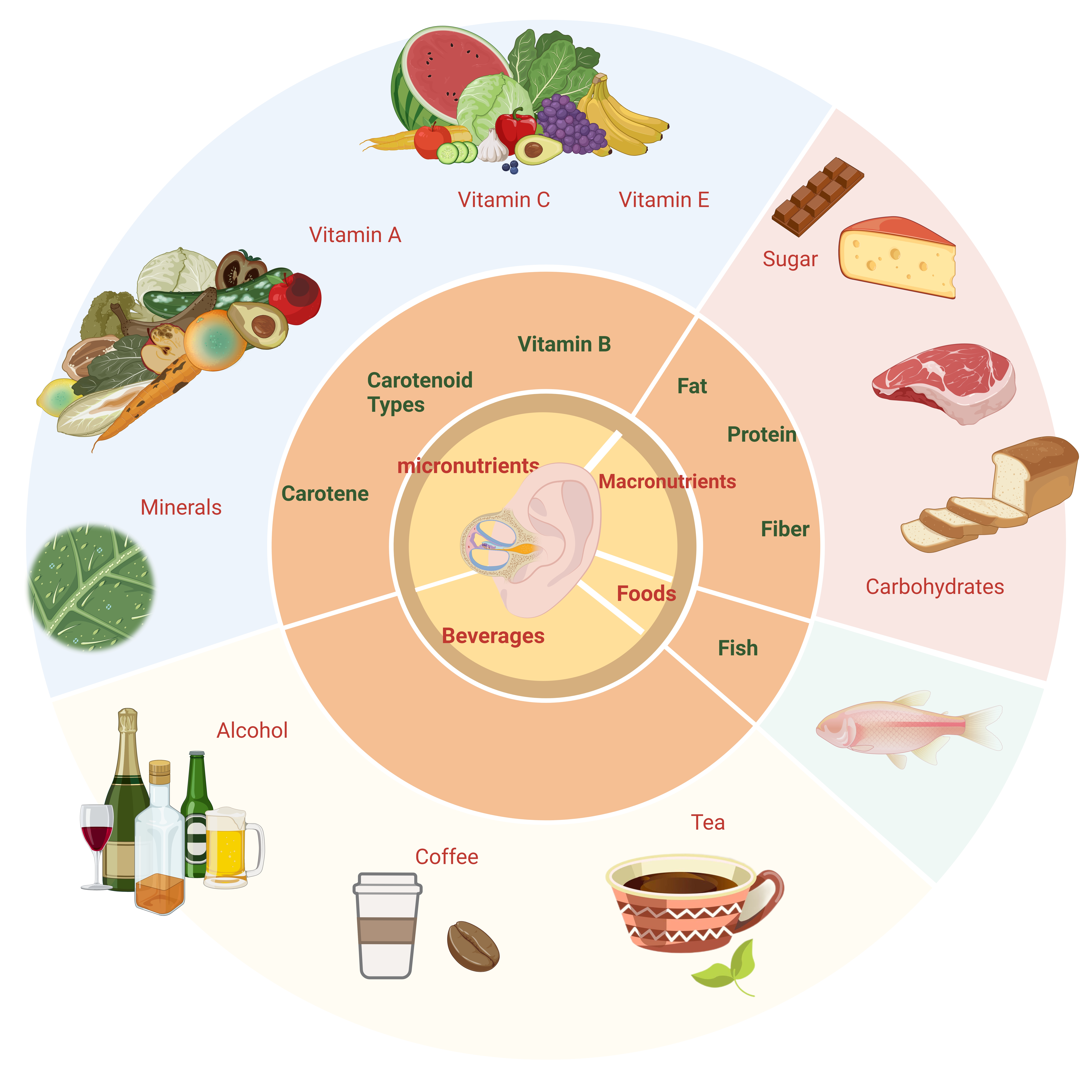

Supplement: Supplementary file 1 [file Data_Sheet_1.zip › 补充文件/Figure 1 Graphical summary.jpg]

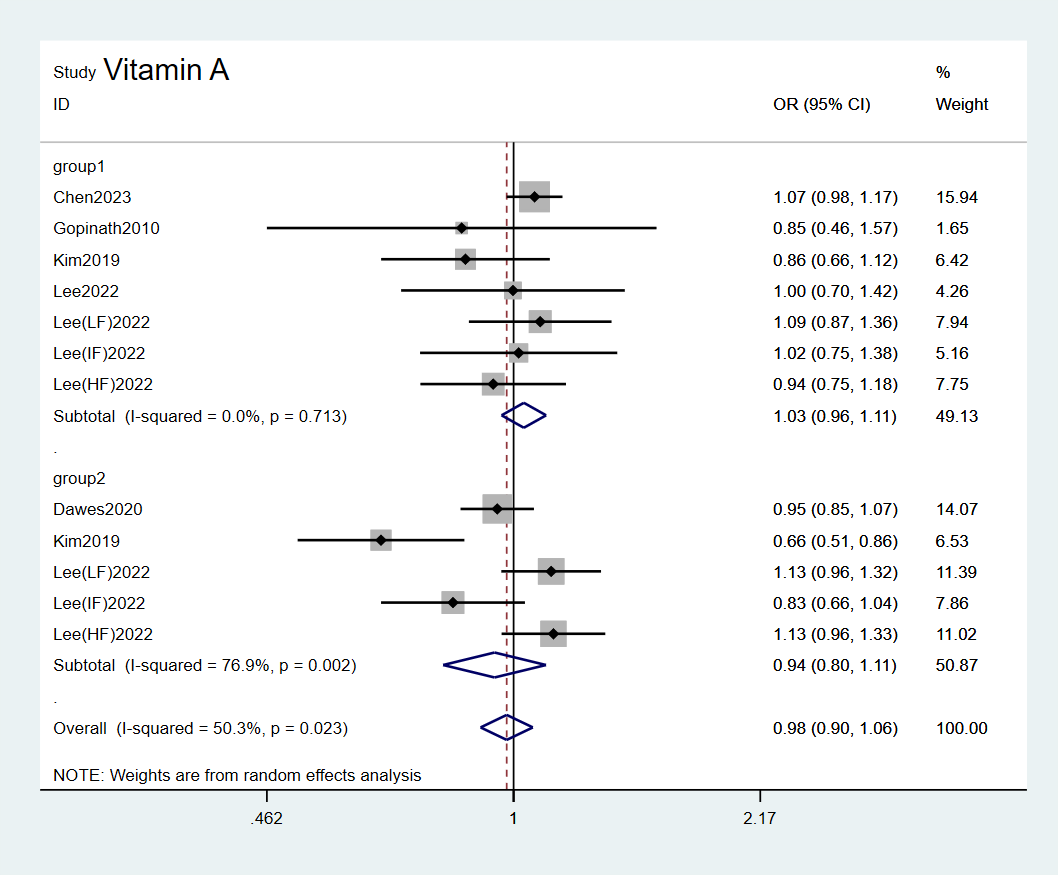

Supplement: Supplementary file 1 [file Data_Sheet_1.zip › 补充文件/Figure S1 Forest maps for Vitamin A intake and incidence of hearing loss.tif]

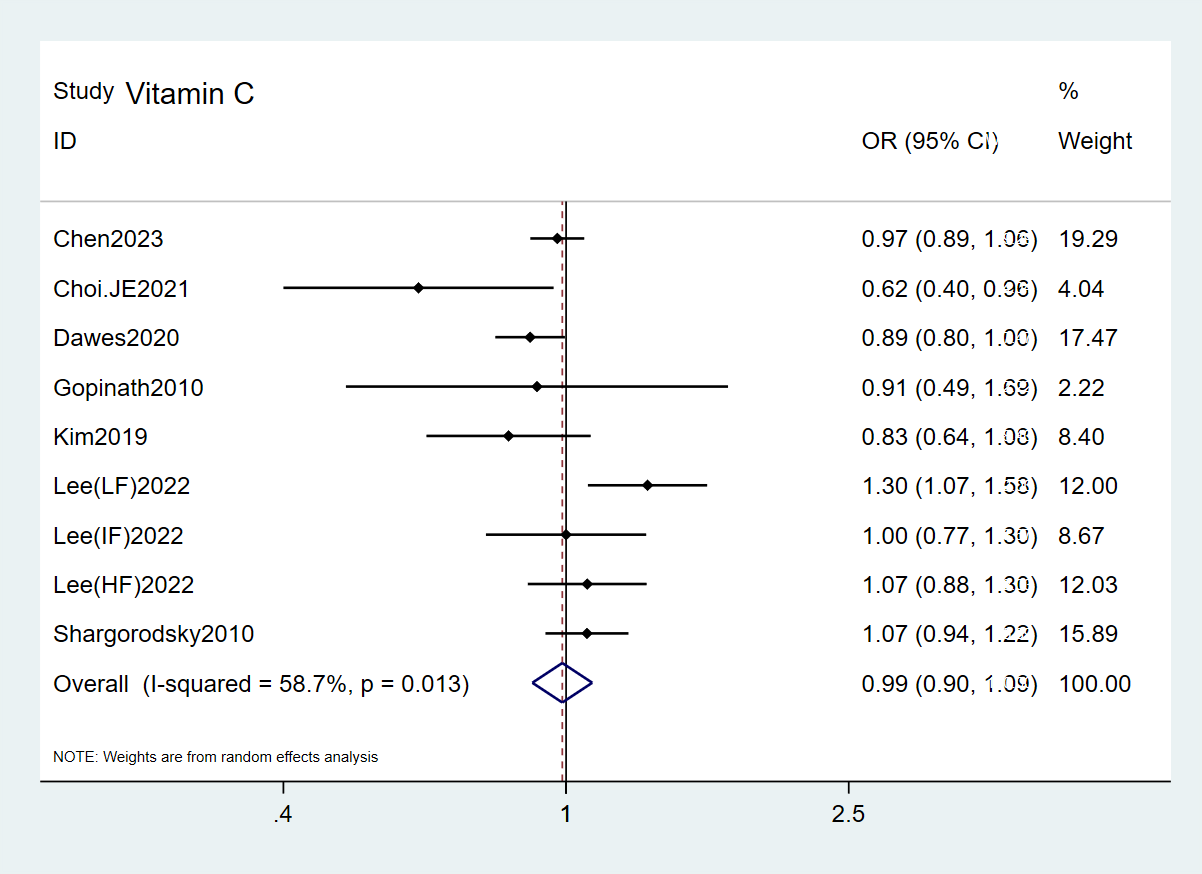

Supplement: Supplementary file 1 [file Data_Sheet_1.zip › 补充文件/Figure S2 Forest maps for Vitamin C intake and incidence of hearing loss.tif]

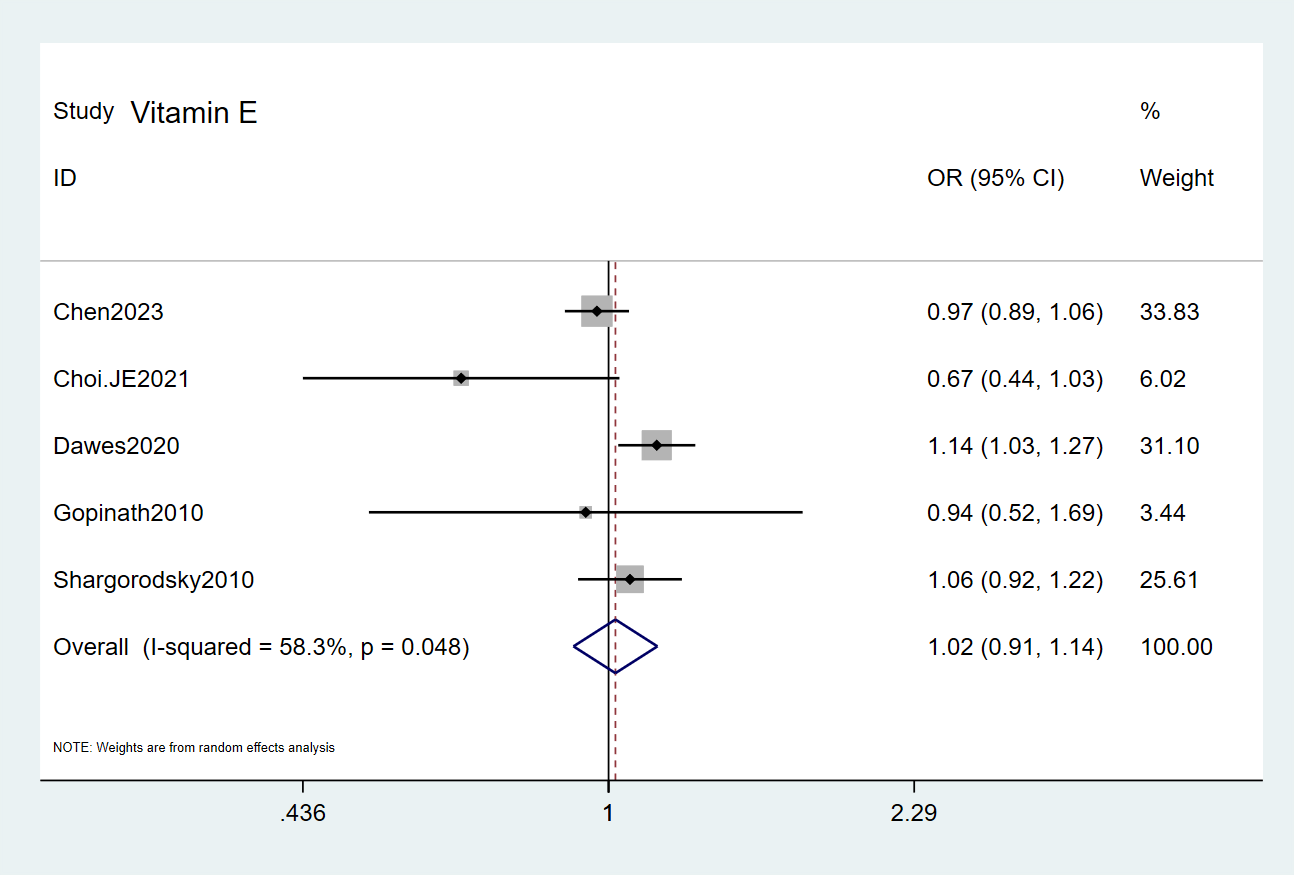

Supplement: Supplementary file 1 [file Data_Sheet_1.zip › 补充文件/Figure S3 Forest maps for Vitamin E intake and incidence of hearing loss.tif]

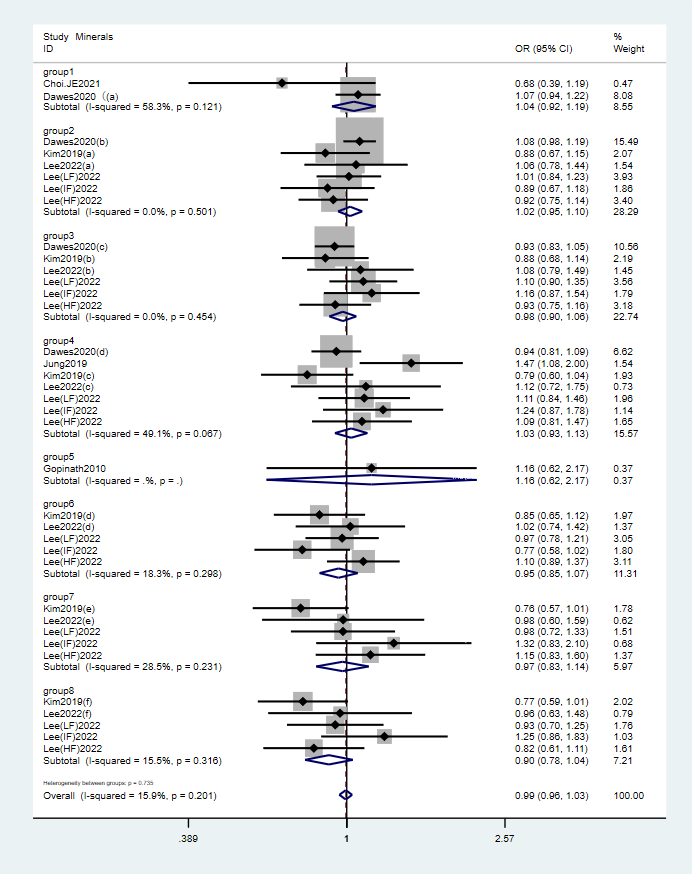

Supplement: Supplementary file 1 [file Data_Sheet_1.zip › 补充文件/Figure S4 Forest maps for Minerals intake and incidence of hearing loss.tif]

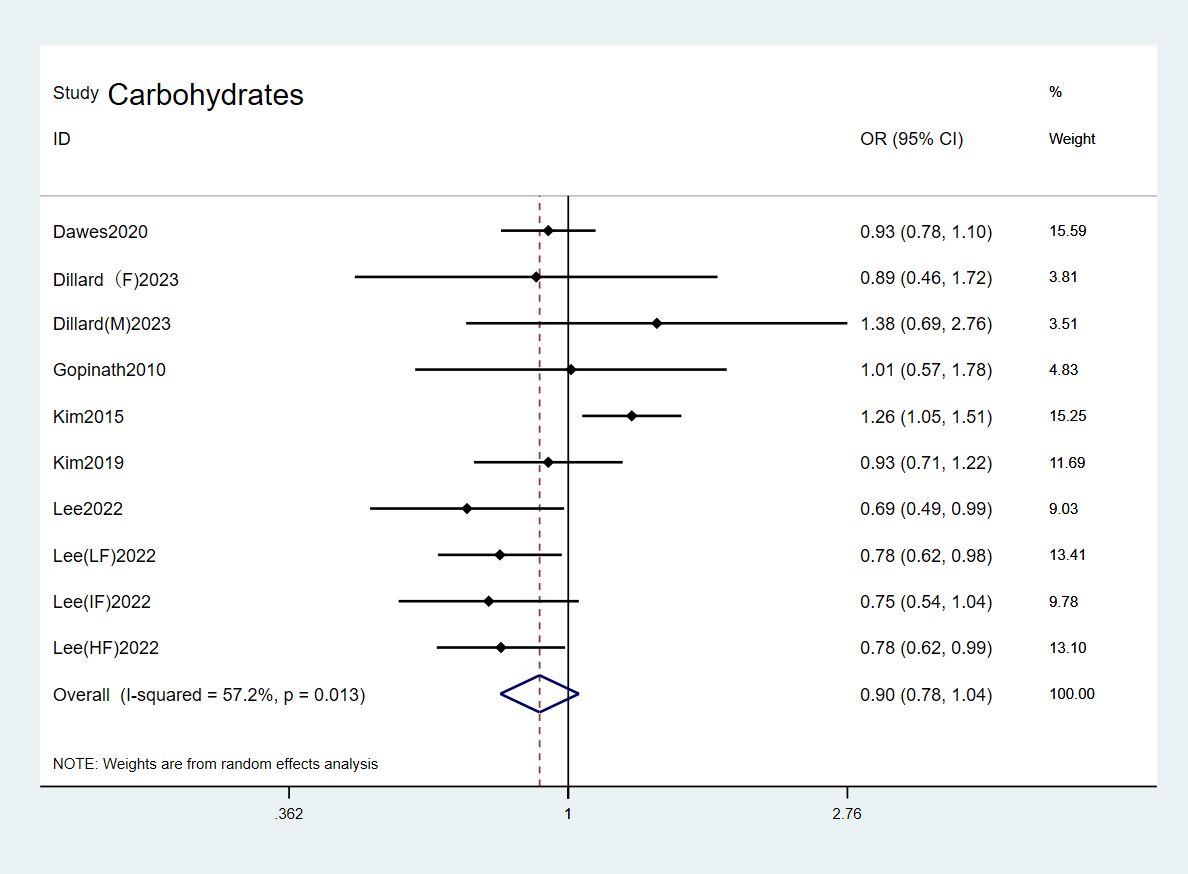

Supplement: Supplementary file 1 [file Data_Sheet_1.zip › 补充文件/Figure S5 Forest maps for Carbohydrates intake and incidence of hearing loss.tif]

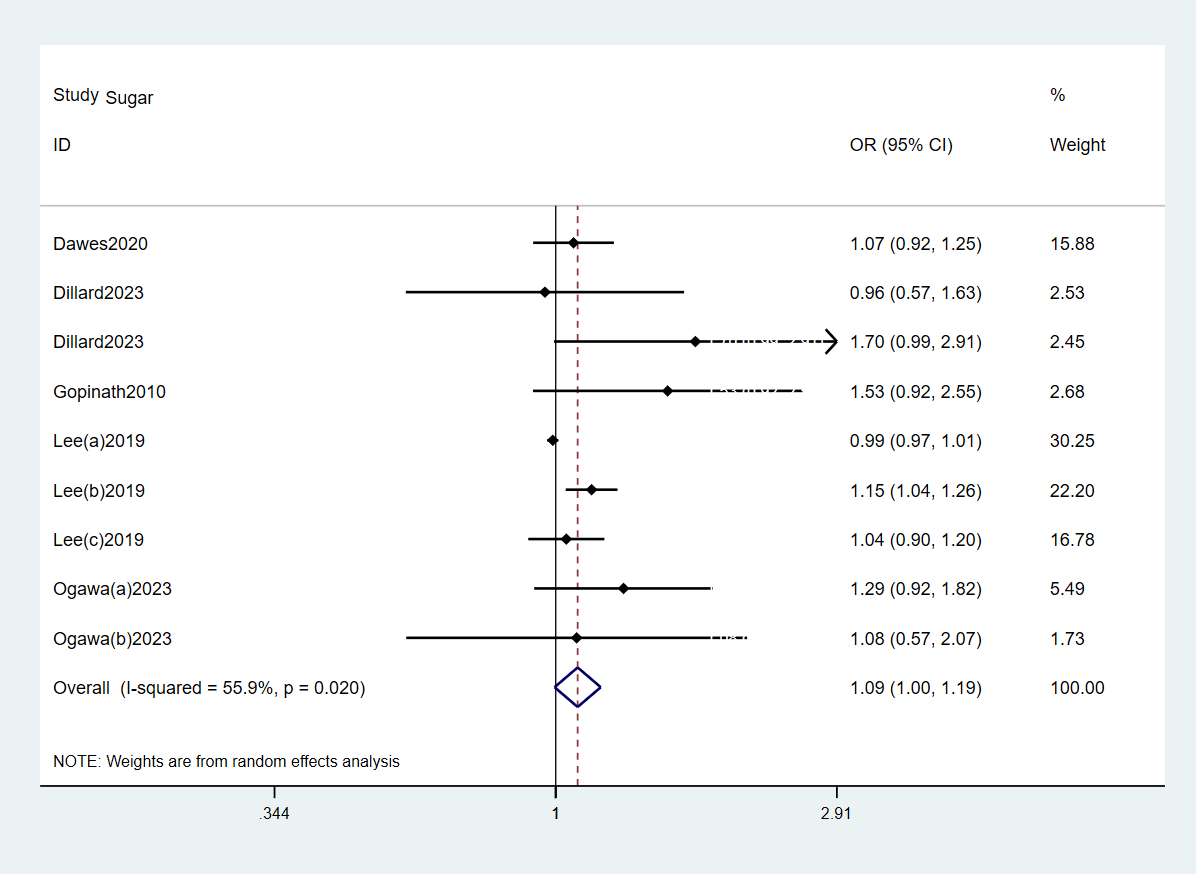

Supplement: Supplementary file 1 [file Data_Sheet_1.zip › 补充文件/Figure S6 Forest maps for Sugar intake and incidence of hearing loss.tif]

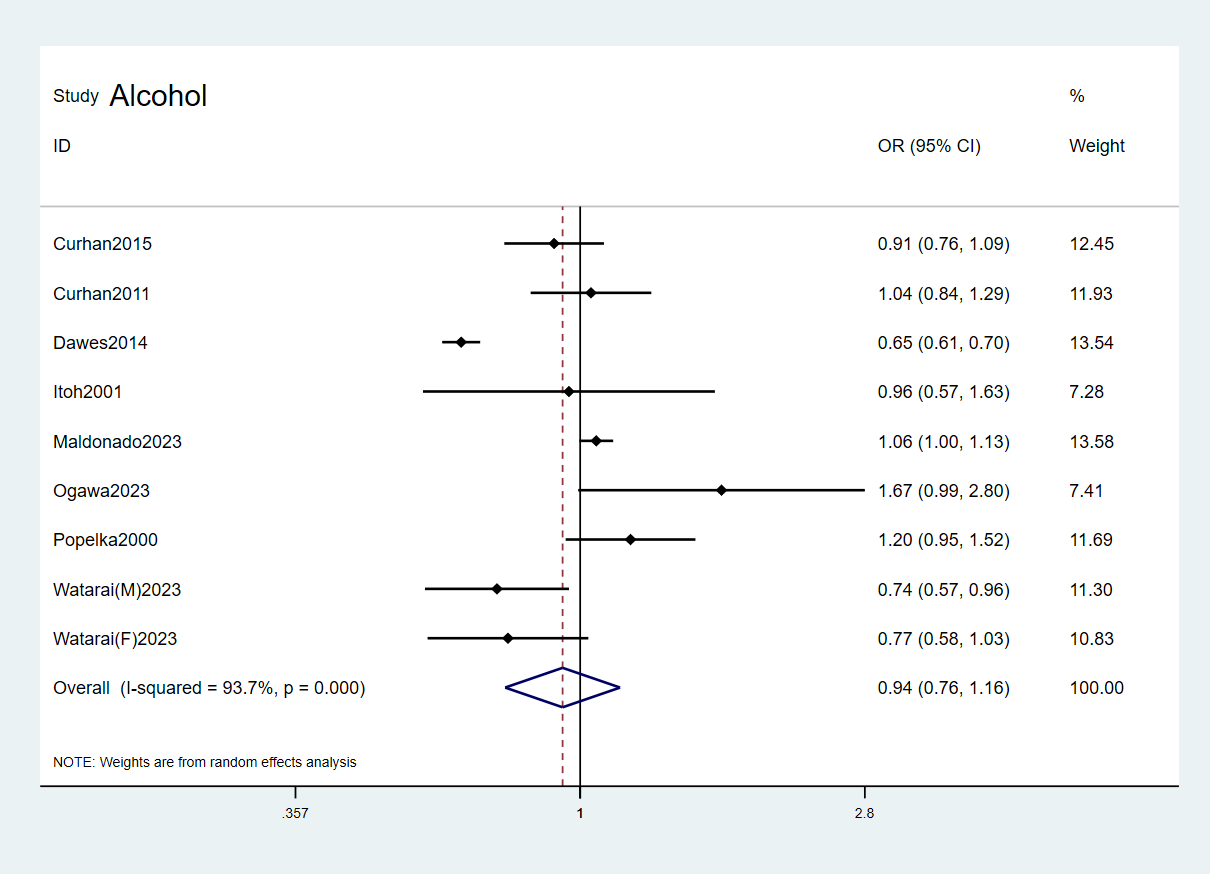

Supplement: Supplementary file 1 [file Data_Sheet_1.zip › 补充文件/Figure S7 Forest maps for Alcohol intake and incidence of hearing loss.tif]

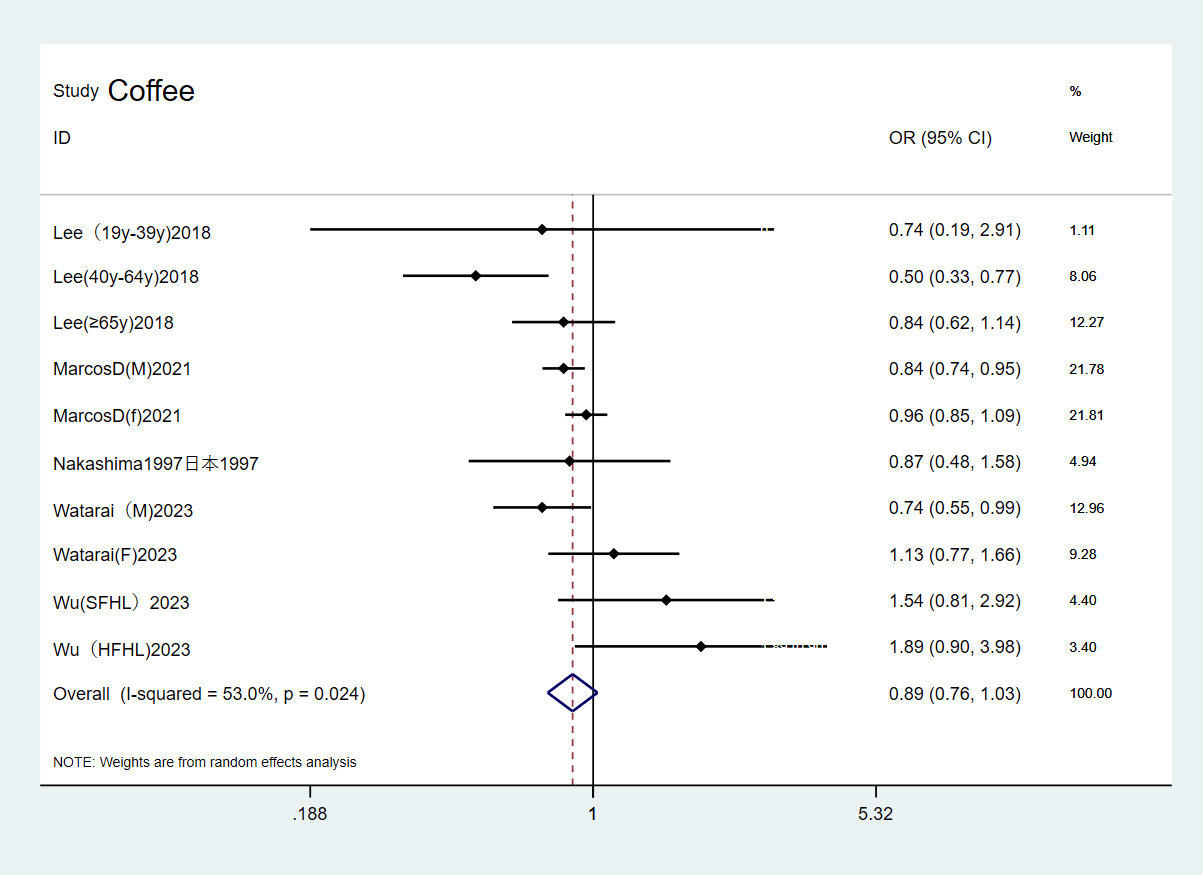

Supplement: Supplementary file 1 [file Data_Sheet_1.zip › 补充文件/Figure S8 Forest maps for Coffee intake and incidence of hearing loss.tif]

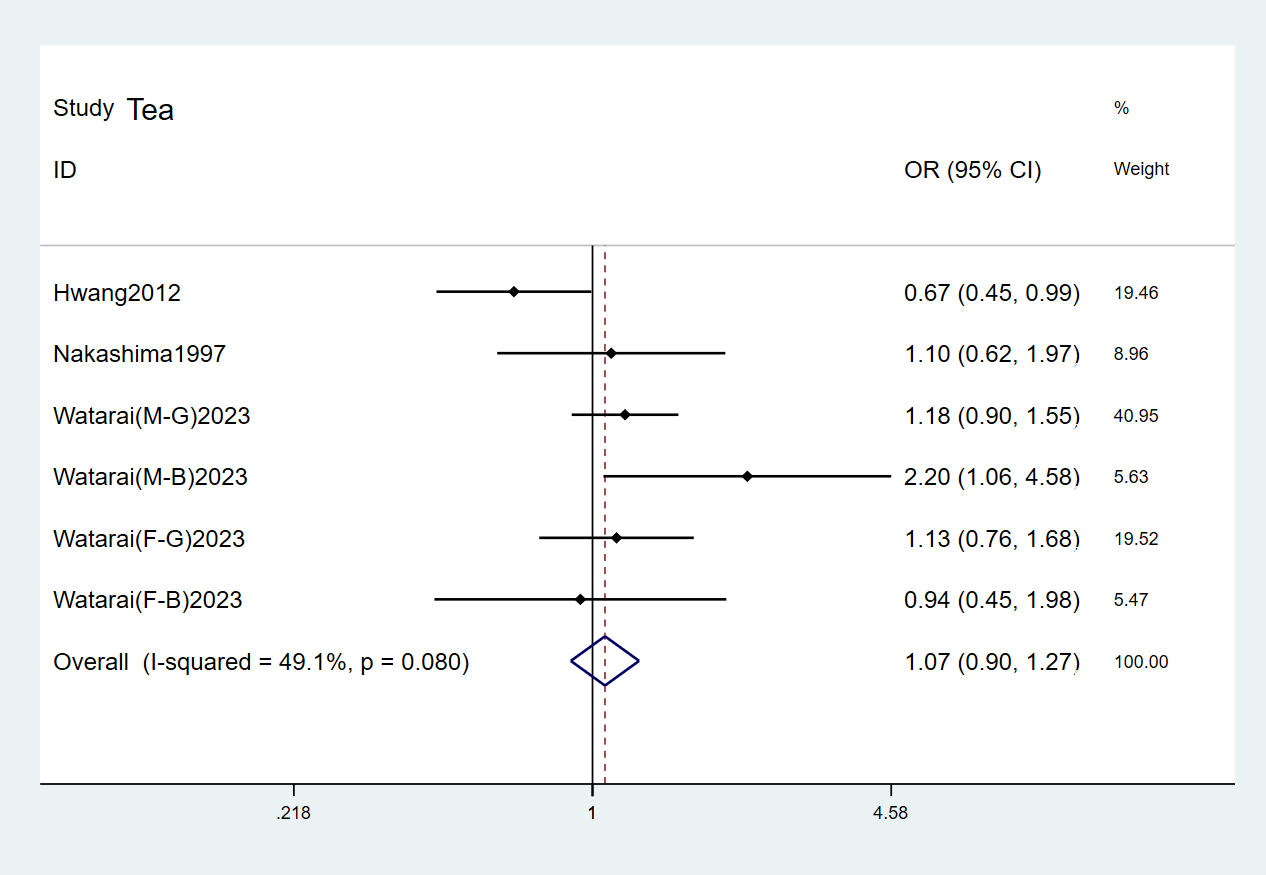

Supplement: Supplementary file 1 [file Data_Sheet_1.zip › 补充文件/Figure S9 Forest maps for Tea intake and incidence of hearing loss.tif]
